# Supplementary material for: Evaluating the feasibility, effectiveness and costs of implementing person-centred follow-up care for childhood cancer survivors in four European countries: the PanCareFollowUp Care prospective cohort study protocol
Source: BMJ Open. 2022 Nov 16;12(11):e063134. doi: 10.1136/bmjopen-2022-063134 (PMC9677022; doi:10.1136/bmjopen-2022-063134)
Supplement: Supplementary data [file bmjopen-2022-063134supp001.pdf]

Appendix A: Recruitment strategy of each study site

Sweden starts with inviting a random sample, prioritising survivors who are lost to follow-up or have not visited the study site in the past five years, and might invite survivors who received care more recently depending on the recruitment rate among the initial population.

Italy starts with inviting survivors who already have a scheduled appointment at their clinic, but who had not already received the Survivorship Passport, and are resident in the Liguria region. They will invite 350 to 400 survivors to be able to include 200 survivors. They will subsequently recruit scheduled survivors resident in other regions, and if the number is still insufficient, they will actively invite other survivors to the clinic.

The Czech Republic starts with selecting a random sample of 250 survivors from the clinic's database whom they will gradually invite over the recruitment period. If more survivors need to be invited to reach the inclusion aim within the recruitment period, they will invite survivors who have a scheduled appointment at their clinic and who meet the study inclusion criteria

Belgium starts to invite, in alphabetical order the survivors of 18 year and older with a primary cancer diagnosis with a date of diagnosis in or before 1990, regardless of whether or not they already received some long-term follow-up. Simultaneously, 20 survivors who were scheduled for a clinic visit in March and April 2021 have also been invited to participate in this study. In the second wave, they will invite the survivors with a diagnosis in 1990-2000 in alphabetic order. And, if needed, the survivors diagnosed in 2001-2020, again in alphabetic order.
