# Supplementary material for: Evaluating the feasibility, effectiveness and costs of implementing person-centred follow-up care for childhood cancer survivors in four European countries: the PanCareFollowUp Care prospective cohort study protocol
Source: BMJ Open. 2022 Nov 16;12(11):e063134. doi: 10.1136/bmjopen-2022-063134 (PMC9677022; doi:10.1136/bmjopen-2022-063134)
Supplement: Author's manuscript [file bmjopen-2022-063134.draft_revisions.pdf]

BMJ Open

BMJ Open is committed to open peer review. As part of this commitment we make the peer review history of every article we publish publicly available.

When an article is published we post the peer reviewers' comments and the authors' responses online. We also post the versions of the paper that were used during peer review. These are the versions that the peer review comments apply to.

The versions of the paper that follow are the versions that were submitted during the peer review process. They are not the versions of record or the final published versions. They should not be cited or distributed as the published version of this manuscript.

BMJ Open is an open access journal and the full, final, typeset and author-corrected version of record of the manuscript is available on our site with no access controls, subscription charges or pay-per-view fees (http://bmjopen.bmj.com).

If you have any questions on BMJ Open's open peer review process please email info.bmjopen@bmj.com

BMJ Open

Evaluating the feasibility and cost-effectiveness of implementing person-centred follow-up care for childhood cancer survivors in four European countries: the PanCareFollowUp Care prospective cohort study protocol

Journal:	*BMJ Open*
Manuscript ID	bmjopen-2022-063134
Article Type:	Protocol
Date Submitted by the Author:	22-Mar-2022
Complete List of Authors:	van Kalsbeek, Rebecca; Princess Maxima Center for Pediatric Oncology<br>Korevaar, J; Netherlands Institute for Health Services Research (NIVEL)<br>Rijken, Mieke; Netherlands Institute for Health Services Research (NIVEL); University of Eastern Finland - Kuopio Campus, Department of Health and Social Care Management<br>Haupt, Riccardo; IRCCS Istituto Giannina Gaslini, Epidemiology and Biostatistics Unit and DOPO clinic<br>Muraca, Monica; IRCCS Istituto Giannina Gaslini, Epidemiology and Biostatistics Unit and DOPO clinic<br>Kepák, Tomáš; Masaryk University Faculty of Medicine, International Clinical Research Centre (FNUSA-ICRC) at St. Anne's University Hospital<br>Kepakova, Katerina; Masaryk University Faculty of Medicine, International Clinical Research Centre (FNUSA-ICRC) at St. Anne's University Hospital<br>Blondeel, Anne; European Society for Pediatric Oncology (SIOP Europe)<br>Boes, Stefan; University of Lucerne, Department of Health Sciences and Medicine<br>Frederiksen, Line Elmerdahl; Danish Cancer Society Research Center, Childhood Cancer Research Group<br>Essiaf, Samira; European Society for Pediatric Oncology (SIOP Europe)<br>Winther, Jeanette; Danish Cancer Society Research Center, Childhood Cancer Research Group; Aarhus Universitet, Department of Clinical Medicine and Faculty of Health<br>Hermens, Rosella; Radboudumc, Scientific Institute for Quality of Healthcare (IQ Healthcare)<br>Kienesberger, Anita; Childhood Cancer International Europe<br>Loonen, Jacqueline; Radboudumc, Department of Hematology<br>Michel, Gisela; University of Lucerne, Department of Health Sciences and Medicine<br>Mulder, Renée; Princess Maxima Center for Pediatric Oncology<br>O'Brien, Kylie; Pintail, Limited<br>van der Pal, Helena; Princess Maxima Center for Pediatric Oncology; PanCare<br>Pluijm, Saskia ; Princess Maxima Center for Pediatric Oncology<br>Roser, Katharina; University of Lucerne<br>Skinner, Roderick; Newcastle University Centre for Cancer, Wolfson Childhood Cancer Research Centre; Great North Children's Hospital, Royal Victoria Infirmary

	Renard, Marleen; KU Leuven University Hospitals Leuven, Department of Paediatric Haematology and Oncology<br>Uyttebroeck, Anne; KU Leuven University Hospitals Leuven, Department of Paediatric Haematology and Oncology<br>Follin, Cecilia; Skane University Hospital, Department of Clinical Sciences Lund, Oncology<br>Hjorth, Lars; Skane University Hospital, Department of Clinical Sciences Lund, Paediatrics<br>Kremer, Leontien; Princess Maxima Center for Pediatric Oncology; Emma Childrens' Hospital UMC, Department of Paediatrics
Keywords:	International health services < HEALTH SERVICES ADMINISTRATION & MANAGEMENT, Health economics < HEALTH SERVICES ADMINISTRATION & MANAGEMENT, Paediatric oncology < ONCOLOGY, Paediatric oncology < PAEDIATRICS, Organisation of health services < HEALTH SERVICES ADMINISTRATION & MANAGEMENT

SCHOLARONE™
Manuscripts

Evaluating the feasibility and cost-effectiveness of implementing person-centred follow-up care for childhood cancer survivors in four European countries: the PanCareFollowUp Care prospective cohort study protocol

Rebecca J. van Kalsbeek[a]*, Joke C. Korevaar[b]*, Mieke Rijken[b,c], Riccardo Haupt[d], Monica Muraca[d], Tomas Kepak[e], Katerina Kepakova[e], Anne Blondeel[f], Stefan Boes[g], Line Elmerdahl Frederiksen[h], Samira Essiaf[f], Jeanette Falck Winther[h,i], Rosella P.M.G. Hermens[j], Anita Kienesberger[k], Jacqueline J. Loonen[l], Gisela Michel[g], Renée L. Mulder[a], Kylie O'Brien[m], Helena J.H. van der Pal[a,n], Saskia M.F. Pluijm[a], Katharina Roser[g], Roderick Skinner[o,p,q], Marleen Renard[r], Anne Uyttebroeck[r], Cecilia Follin[s], Lars Hjorth[t], Leontien C. M. Kremer[a,u,v]

** Shared first authorship*

Affiliations:

[a] Princess Máxima Centre for Paediatric Oncology, Heidelberglaan 25, 3584 CS, Utrecht, the Netherlands

[b] Netherlands Institute for Health Services Research (Nivel), P.O. Box 1568, 3500 BN Utrecht, the Netherlands

[c] University of Eastern Finland, Department of Health and Social Management, P.O. Box 1627, FI-70211 Kuopio, Finland

[d] Epidemiology and Biostatistics Unit and DOPO clinic, IRCCS Istituto Giannina Gaslini, Via G. Gaslini, 5, 16147 Genoa, Italy

[e] International Clinical Research Centre (FNUSA-ICRC) at St. Anne's University Hospital, Masaryk University, Pekařská 53, Brno 656 91, Czech Republic

[f] European Society for Paediatric Oncology, c/o BLSI, Clos Chapelle-aux-Champs 30, Bte 1.30.30, BE-1200 Brussels, Belgium

[g] University of Lucerne, Department of Health Sciences and Medicine, Frohburgstrasse 3, P.O. Box 4466, 6002 Lucerne, Switzerland

[h] Childhood Cancer Research Group, Danish Cancer Society Research Centre, Strandboulevarden 49, 2100 Copenhagen, Denmark

For peer review only - http://bmjopen.bmj.com/site/about/guidelines.xhtml

[i] Department of Clinical Medicine, Faculty of Health, Aarhus University and Aarhus University Hospital, Palle Juul-Jensens Boulevard 82, 8200 Aarhus, Denmark

[j] Radboud University Medical Centre, Radboud Institute for Health Sciences, Scientific Institute for Quality of Healthcare (IQ Healthcare), Geert Grooteplein 21, 6525 EZ, Nijmegen, the Netherlands

[k] Childhood Cancer International Europe, Servitengasse 5/16, 1090 Vienna, Austria

[l] Radboud University Medical Centre, Radboud Institute for Health Sciences, Department of Hematology, Geert Grooteplein Zuid 10, 6525 GA, Nijmegen, the Netherlands

[m] Pintail, Limited, 77 Springhill Avenue, Blackrock, Dublin, Ireland

[n] PanCare, Jacobus Bellamylaan 16, 1401 AZ Bussum, the Netherlands

[o] Newcastle University Centre for Cancer, Wolfson Childhood Cancer Research Centre, Herschel Building, Brewery Lane, Newcastle upon Tyne, NE1 7RU, United Kingdom

[p] Great North Children's Hospital, Royal Victoria Infirmary, Queen Victoria Road, Newcastle upon Tyne, NE1 4 LP, United Kingdom

[q] Translational and Clinical Research Institute, Wolfson Childhood Cancer Research Centre, Herschel Building, Brewery Lane, Newcastle upon Tyne, NE1 7RU, United Kingdom

[r] Department of Oncology, Paediatric Oncology, KU Leuven, Department of Paediatric Haematology and Oncology, University Hospitals Leuven, Herestraat 49, 3000 Leuven, Belgium

[s] Lund University, Skane University Hospital, Department of Clinical Sciences Lund, Oncology, Lasarettsgatan 40, 221 85 Lund, Sweden

[t] Lund University, Skane University Hospital, Department of Clinical Sciences Lund, Paediatrics, Lasarettsgatan 40, 221 85 Lund, Sweden

[u] Department of Paediatrics, Emma Children's Hospital, Amsterdam UMC, Meibergdreef 9, 1105 AZ Amsterdam, the Netherlands

[v] Faculty of Medicine, Utrecht University and Utrecht Medical Centre, Universiteitsweg 98, 3584 CG Utrecht, the Netherlands

Corresponding author: Rebecca J. van Kalsbeek
R.J.vanKalsbeek@prinsesmaximacentrum.nl

For peer review only

Princess Máxima Center for Pediatric Oncology
Heidelberglaan 25
3584 CS Utrecht, The Netherlands
+ 31 88 972 72 72

Public title: Impact of person-centred follow-up care for European childhood cancer survivors: PanCareFollowUp Care

Keywords: Childhood cancer; survivorship; person-centered care; shared decision-making; empowerment; quality of life; clinical trial protocol; cohort study; prospective study; multicenter study, cost effectiveness

Word count: 3935 words (including Box 1)

Abstract word count: 297 words

Tables: 1

Figures: 2

Appendices: 1

For peer review only - http://bmjopen.bmj.com/site/about/guidelines.xhtml

List of abbreviations:

FAIR = Findable, accessible, interoperable and reusable

GCP = Good Clinical Practice

HCP(s) = Health care provider(s)

ICER(s) = Incremental cost-effectiveness ratio(s)

IGHG = International Late Effects of Childhood Cancer Guideline Harmonization Group

PanCare = Pan-European Network for Care of Survivors after Childhood and Adolescent Cancer

PROM = Patient-reported outcome measure

PREM = Patient-reported experience measure

RE-AIM = Reach, Effectiveness, Adoption, Implementation and Maintenance

SD = Standard deviation

SurPass = Survivorship Passport

T1-5 = Time points 1-5

For peer review only

Abstract

Introduction – Long-term survival after childhood cancer often comes at the expense of late, adverse health conditions. However, survivorship care is often not available for adult survivors in Europe. The PanCareFollowUp Consortium therefore developed the PanCareFollowUp Care Intervention, an innovative person-centred survivorship care model based on experiences in the Netherlands. This paper describes the protocol of the prospective cohort study (Care Study) to evaluate the feasibility and the health economic, clinical and patient-reported outcomes of implementing PanCareFollowUp Care as usual care in four European countries.

Methods and analysis – In this prospective, longitudinal cohort study with at least six months of follow-up, 800 childhood cancer survivors will receive the PanCareFollowUp Care Intervention across four study sites in Belgium, Czech Republic, Italy and Sweden, representing different health care systems. The PanCareFollowUp Care Intervention will be evaluated according to the RE-AIM (Reach, Effectiveness, Adoption, Implementation and Maintenance) framework. Clinical and research data are collected through questionnaires, a clinic visit for multiple medical assessments and a follow-up call. The primary outcome is empowerment, assessed with the Health Education Impact Questionnaire (HEIQ). A central data centre will perform quality checks, data cleaning, data validation, and provide support in data analysis. Multilevel models will be used for repeated outcome measures, with subgroup analysis, e.g. by centre, attained age, sex or diagnosis.

Ethics and dissemination - This study will be conducted in accordance with the guidelines of Good Clinical Practice and the Declaration of Helsinki. The study protocol has been reviewed and approved by all relevant ethics committees. The evidence and insights gained by this study will be summarised in a Replication Manual, also including the tools required to implement the PanCareFollowUp Care Intervention in other countries. This Replication Manual will become freely available through PanCare and will be disseminated through policy and press releases.

Trial registration - NL8918, registered at the Netherlands Trial Register at 24 September 2020, https://www.trialregister.nl/trial/8918.

For peer review only - http://bmjopen.bmj.com/site/about/guidelines.xhtml

Article summary

Strengths and limitations of this study

- The PanCareFollowUp Care Study is designed and conducted together with survivor representatives, ensuring the outcome measures are relevant for survivors and that PanCareFollowUp Care meets their needs and expectations.
- We include survivors from four different European countries, representing a variety of health care systems across Europe; and their experiences are used to improve the PanCareFollowUp Care Intervention before free distribution of the materials in a Replication Manual.
- The PanCareFollowUp Care Intervention is evaluated in a real life setting with a minimal number of exclusion criteria.
- Since the Care Study has a limited follow-up time, a model-based economic evaluation will complement the analyses.
- Participants are their own controls and effects are evaluated as changes from baseline within an individual or institution.

For peer review only

For peer review only - http://bmjopen.bmj.com/site/about/guidelines.xhtml

Introduction

Over the last decades, five-year survival rates of childhood cancer in Europe have increased substantially, from 30% in the 1970s to 80% in the early 2000s (1). Today, the European population of childhood cancer survivors, estimated at minimally 300,000, is rising by about 12,000 per year (2). Yet, many survivors not only experience the burden of previous cancer diagnosis, but also face treatment-related late effects (3, 4). These may become apparent years or even decades after finishing therapy (5) and might have a significant adverse impact on quality of life (6, 7). Moreover, the transition from paediatric to adult health care settings often lacks continuity. As a result, many adults who survived childhood cancer have increased health care use and experience problems in participation, which generate a substantial burden for survivors and societies in general (8-10). Early detection of new health conditions is essential as it could prevent further harm (11). This requires lifelong survivorship care with frequent adaptations of the follow-up plan.

Currently, only one third of European paediatric oncology clinics provide survivorship care to adult survivors of childhood cancer (12). In 2006, an international group of paediatric oncologists, psychologists, nurses, epidemiologists, survivors and their parents agreed in the Erice statement that has recently been updated and reconfirmed (13, 14) that follow-up care should be available and accessible for all survivors throughout their lifespan.

In the past decade, international evidence-based clinical practice guidelines have been developed to support early detection and treatment of (a)symptomatic late effects, including those developed by the International Late Effects of Childhood Cancer Guideline Harmonization Group (IGHG), sometimes in collaboration with the PanCareSurFup project (15-23). A European model of care guideline is published and guidelines for the transition from childhood to adult healthcare settings and health promotion are currently being developed (24, 25). Yet, implementation lags behind. Recently, a person-centred approach for survivorship care for adult survivors has been implemented in Nijmegen, the Netherlands (26). All Dutch survivors of childhood cancer are invited for follow-up care by a long-term follow-up care clinic, in which multidisciplinary teams deliver person-centred care based on contemporary surveillance guidelines (27). The first positive effects of this person-centred approach have been reported (24, 26). The next step is to validate this person-centred approach for survivorship care in other countries.

The PanCareFollowUp Consortium, established in 2018, is a unique multidisciplinary European collaboration between 14 project partners from 10 European countries, including survivors (www.pancarefollowup.eu) (28). The aim of the consortium is to improve the quality of life for

For peer review only - http://bmjopen.bmj.com/site/about/guidelines.xhtml

survivors of childhood, adolescent and young adult cancer by bringing evidence-based, person-centred care to clinical practice. The PanCareFollowUp Consortium has developed two interventions: 1) a person-centred and guideline-based model of survivorship care (PanCareFollowUp Care Intervention) (see Box 1) (29) and 2) an eHealth lifestyle coaching model (PanCareFollowUp Lifestyle Intervention). The protocol of the first intervention is described in this paper (version 3, January 21st, 2021), the protocol of the second one will be described separately. Both will be evaluated within the PanCareFollowUp project. The consortium published a Care Intervention Manual that contains instructions and tools required for implementing the PanCareFollowUp Care Intervention. At the project end, Replication Manuals that contain the instructions and tools required for implementation of the PanCareFollowUp Interventions will be freely distributed.

The overall aim of the PanCareFollowUp Care Study is to evaluate the feasibility and (cost-) effectiveness of implementing PanCareFollowUp Care as usual care for adult survivors of childhood cancer in four study sites in four European countries. Four objectives have been formulated: 1) To what extent is implementing PanCareFollowUp Care in the participating study sites feasible?; 2) What are the patient-reported experiences and outcomes, including survivor empowerment, of PanCareFollowUp Care and how do they change?; 3) What is the number and nature of pre-existing and new clinical events detected by PanCareFollowUp Care among participating survivors?; and 4) What is the cost-effectiveness and cost-utility of implementing PanCareFollowUp Care relative to usual care from the perspective of survivors, health care providers (HCPs), and society at large?

Box 1: The PanCareFollowUp Care Intervention

The PanCareFollowUp Care Intervention is based on a person-centred care model (26) that aims to meet the physical, psychological and social needs of (adult) survivors of childhood cancer through shared decision-making about prevention, surveillance and treatment options. The Care Intervention consists of three steps:

a) *Preparation of the clinic visit by both the survivor and the health care provider (HCP).* The survivor provides information about their health, wellbeing, needs and preferences by completing the PanCareFollowUp Survivor Questionnaire. The HCP prepares a Treatment Summary describing the childhood cancer treatment that the survivor has received, reviews the relevant surveillance recommendations and the PanCareFollowUp Survivor Questionnaire provided by the survivor, and thereupon prepares the Standard Survivorship Care Plan.

For peer review only - http://bmjopen.bmj.com/site/about/guidelines.xhtml

b) *Clinic visit including tailored follow-up care.* After obtaining a medical history and performing a physical examination, the survivor and HCP jointly discuss the results of the Survivor Questionnaire, and the Standard Survivorship Care Plan. Together, they agree on a plan for diagnostic tests and potential referral if needed, based on surveillance guidelines or clinical indication. Based on these shared decisions, as well as potential test results, the HCP creates a Draft Individualised Survivorship Care Plan and provides tailored health education.

c) *Follow-up call.* The survivor and HCP discuss the test results and the preferred model of care for future follow-up care. The results of these shared decisions are incorporated in the final Individualised Survivorship Care Plan, that the survivor may share with other HCPs.

The PanCareFollowUp Care Intervention ends after co-creation and delivery of the Individualised Survivorship Care Plan. Survivors will thereafter remain under surveillance either at or under the guidance of their clinic, frequently adjusting their Individualised Survivorship Care Plan when needed.

Methods and analysis

Study population, setting and recruitment

Survivors fulfil the inclusion criteria if they are or have been: diagnosed with cancer before the age of 19 years; treated or registered at one of the four study sites; treated with chemotherapy and/or radiation therapy for childhood cancer with or without surgery; at least five years from primary cancer diagnosis; at least one year off treatment (also applying to treatment of subsequent benign or malignant neoplasms or relapse of the primary cancer); and currently at least 16 years of age.

Exclusion criteria consist of: being unable to complete the study questionnaires because of severe neurocognitive sequelae or insufficient understanding of the language used (even with help from another person); or having previously received complete follow-up care that is similar to the care as described in the PanCareFollowUp Care Intervention Manual (Box 1).

This international prospective cohort study will be conducted at four study sites located in four European countries: Belgium, Czech Republic, Italy, and Sweden. All sites currently provide long-term follow-up care, either within a paediatric (Belgium, Italy) or adult (Czech Republic, Sweden) oncology centre, using a set of (inter)national guidelines and protocols. Each study site aims to include 200 survivors who complete the study. With an estimated non-response and early drop-out (informed consent signed, but no actual participation in the study) of 40 to 50% based on previous

For peer review only - http://bmjopen.bmj.com/site/about/guidelines.xhtml

experience and an estimated late drop-out (at any point after completing the T1 questionnaire) of 5-10% during the study, approximately 350 to 400 survivors will therefore be invited at each site. To assess the feasibility of this recruitment strategy, each centre screened their respective registries and estimated a total of 5,944 eligible survivors.

Each study site developed a recruitment strategy within the prerequisites of this study, that fits best within their own logistics (Appendix A). Selected survivors will be invited by an invitation letter, an invitation e-mail or by phone (depending on the usual procedure at each study site), and receive an information sheet, including contact details for additional information, and an informed consent form. Reasons for non-participation can be provided. One option of the pre-set reasons is 'not participating because the questionnaires are being provided via internet'. In this case, the study site may decide to offer the option for paper questionnaires. Survivors who give informed consent but do not respond to the first questionnaire, even after reminders, are considered early drop-outs and will be excluded from the study, as essential data about these survivors will not be available. The first participant was enrolled in February 2021, and at 1 March 2022 456 participants were enrolled and completed the clinic visit. The estimated last inclusion is on 30 September 2022, with last data collection 31 May 2023.

Participating survivors can withdraw from the study at any time if they wish. They are not obliged to provide a reason for withdrawal, although it will be asked and recorded if available. To assess representativeness of the final study sample, the four centres will provide aggregated data about their total eligible population of survivors including population distributions of gender, current age, age at diagnosis, type of cancer and distance to the late effects clinic. This will be compared to the distributions among the included survivors per clinic.

During recruitment and data collection, careful monitoring of enrolment, (non-)response, reasons for non-response and early and late drop-out will be performed by the four study sites in close collaboration with the central data centre at the Danish Cancer Society Research Centre.

Intervention

Survivors of childhood cancer who receive PanCareFollowUp Care (i.e., care in accordance with the PanCareFollowUp Care Intervention Manual and as outlined in Box 1) will be followed up until six months after the clinic visit. The implementation of person-centred care in this project is facilitated by a narrated Powerpoint and an on-site workshop for all HCPs involved in the study. An add-on study investigating the feasibility of delivering PanCareFollowUp Care using the digital Survivorship

For peer review only - http://bmjopen.bmj.com/site/about/guidelines.xhtml

Passport (SurPass) tool (30) will be conducted at the Italian clinic, where SurPass is already implemented.

Primary and secondary outcomes

This study uses a variety of outcomes to answer the four research objectives (Figure 1).

1) To what extent is implementing PanCareFollowUp Care in the participating study sites *feasible?*

Feasibility of implementation is of major importance to ensure sustainability of the PanCareFollowUp Care Intervention. Therefore, feasibility indicators as well as an evaluation of barriers and facilitators are included to inform about the experiences of implementing PanCareFollowUp Care, both from the survivor's and the HCP's perspective. These include drop-outs at different time-points, use of and experiences with the Survivorship Care Plan, and shared-decision making.

2) What are the experiences and outcomes as reported by participating survivors receiving PanCareFollowUp Care?

The primary outcome for this study is empowerment measured by the Health Education Impact Questionnaire (HEIQ) (31). Empowerment has been defined by the EU Joint Action on Patient Safety and Quality of Care as a 'multidimensional process that helps people gain control over their own lives and increase their capacity to act on issues that they themselves define as important', a definition adapted from Lutrell et al. (32, 33). Empowerment has been selected as the primary outcome because childhood cancer survivors encounter several transition moments starting from diagnosis, after which a greater responsibility for their own health and care is required. It is essential that survivors receive the support they need to manage and advocate for their needs. Moreover, empowerment is important to manage future health problems.

Secondary outcomes consist of a variety of patient-reported experiences and outcomes (PREMs and PROMs), such as satisfaction and quality of life.

3) What is the number and nature of pre-existing and new clinical events detected by PanCareFollowUp Care among participating survivors?

Clinical outcomes are outcomes of symptoms and diseases and have been defined based on published or almost published guidelines of the IGHG and the PanCareFollowUp Recommendations. A total of 116 clinical outcomes were defined, which reflects the wide range of late effects that survivors may encounter affecting both physical health and psychosocial wellbeing (Figure 1). The

For peer review only - http://bmjopen.bmj.com/site/about/guidelines.xhtml

number and range of pre-existing and newly detected health problems (symptomatic and asymptomatic) per survivor will be described, including the results of clinical examinations (e.g. echocardiogram or blood tests).

4) What is the cost-effectiveness and cost-utility of implementing PanCareFollowUp Care relative to usual care from the perspective of survivors, HCPs, and society at large?

The cost-effectiveness and cost-utility of the care model will be determined. Health economic outcomes reflect the time, time off work and monetary investments made by the survivor, accompanying relatives or friends, the HCP and other staff in relation to the clinic visit while receiving or providing PanCareFollowUp Care. We do not take costs outside the clinic visit into account, i.e., costs related to possible (follow-up) primary care physician visits, mental health services, or referrals to other specialists outside the clinical setting. Costs related to the clinic visit, as associated with PanCareFollowUp Care, are compared to potential benefits measured in terms of PREMs and PROMs.

An overall evaluation of implementing the PanCareFollowUp Care Intervention will be performed throughout the project according to the RE-AIM (Reach, Effectiveness, Adoption, Implementation and Maintenance) framework to assess the impact (www.re-aim.org) (34) (Table 1).

Table 1. RE-AIM framework applied to the PanCareFollowUp Care Intervention.

**Components**	**Related outcomes/actions in the Care Study**
Reach	• No. and proportion of participants vs. non-responders<br>• Representativeness of participating survivors[a] (comparison of distribution: gender, current age, age at diagnosis and type of cancer)<br>• Reasons for (non-)participation
Effectiveness/efficacy	• Main outcome empowerment[a]<br>• Patient-reported outcome and experience measures, and clinical, feasibility and health economic outcomes[a]
Adoption[b]	• Multidisciplinarity of HCPs involved<br>• Recruitment rate<br>• Barriers and facilitators for recruitment
Implementation[b]	• Use of SCP and reasons for non-use

For peer review only - http://bmjopen.bmj.com/site/about/guidelines.xhtml

	• Adaptations made to the PanCareFollowUp Care Intervention or implementation strategy<br>• Time and costs of PanCareFollowUp Care for survivors and HCPs<br>• Barriers and facilitators for implementation
Maintenance	• Replication Manual including updated implementation and recruitment strategy, publicly available for current and new centres<br>• Overview of requirements for study sites to make the PanCareFollowUp Care Intervention routine care

Abbreviations: HCPs = health care providers, SCP = Survivorship Care Plan. [a] Comparisons will be made according to subgroups of gender, current age, age at diagnosis and type of cancer. [b] This information will be collected at each study site separately.

Patient and public involvement

Survivor representatives from Childhood Cancer International-Europe are included in the project as members of the PanCareFollowUp Consortium (28). They are involved throughout the project and reach out to their respective national and international networks when needed. Survivors were involved in setting the research agenda by writing the grant application and the study protocol, developing and reviewing the PanCareFollowUp Care Intervention materials, evaluating the study questionnaires, monitoring the progress of the PanCareFollowUp Care Study and creating awareness on social media (29). They helped consider ways to mitigate the burden of completing the study questionnaires or remembering the childhood cancer history for participants. After the end of data collection, survivor representatives will be involved in the interpretation of the study results and dissemination to participants, survivor networks and the general public.

Power calculation

We aim to include 200 participants at each of the four study sites (total n=800). The primary outcome measure is change in empowerment between T1 and T5 as measured by the HEIQ (35). We use six constructs (cancer version including five constructs plus one additional construct, namely self-monitoring and insight) with mean scores ranging from 2.9 (standard deviation (SD): 0.64) to 3.2 (SD: 0.48). Taking the construct with the largest SD (thus needing the highest number of participants to demonstrate a statistically significant change), limiting it to a single study site, with a 2-sided α of 0.05, a power of 80%, we will need 200 participants to identify an effect size of 0.2 given a mean

For peer review only - http://bmjopen.bmj.com/site/about/guidelines.xhtml

score of 2.9 (SD: 0.64). That is enough power to demonstrate a small to medium effect. The actual power is larger since we ignored measuring empowerment repeatedly, having four centres (800 patients instead of 200) and using constructs with smaller SDs.

Data collection

Data will be collected from participating survivors as well as from their HCPs at five time points (T1-T5) during a follow-up period of six to eight months (Figure 2). We will use data collected in the context of care delivery, and combine it with additional data collected specifically for research purposes. For the latter, there are three data collection moments for survivors and four for HCPs. These time points are linked to the structure of the PanCareFollowUp Care Intervention, which consists of three steps: 1) Preparation of the clinic visit by survivor and HCP (corresponding with T1), 2) Clinic visit (corresponding with T2), and 3) Follow-up call (two to four weeks after T2, corresponding with T3). Thereafter, there is data collection at 1 week after the follow-up call (T4) and 6 months after the clinic visit (T5).

The main data collection instruments consist of the PanCareFollowUp Survivor Questionnaire (care), the Treatment Summary (care), medical history, physical examinations and diagnostic tests during and after the clinic visit (care), and additional online study questionnaires for survivors and HCPs (research). The English versions of the study questionnaires for survivors have been pretested by three survivors, whereas the English questionnaires for HCPs have been pretested with at least two HCPs in each centre before the start of the data collection. The questionnaires for survivors have subsequently been translated to the local languages of the study sites, i.e. Czech, Dutch, Italian and Swedish.

Statistical analysis

For analysing outcomes measured multiple times, like the primary outcome, we will analyse multilevel models for repeated measures applying a fixed effect to control for study site. Next, we will perform subgroup analyses for relevant groups by including interaction terms. These subgroups will be identified based on the literature combined with knowledge from professionals. The final selection will be determined during the study, however, possible subgroups may be distinguished according to centre, sex, time since cancer diagnosis, treatment type, or distance to late effects clinic. The models will be adjusted for confounders, which will be identified during the study based on the literature and expert opinion. Clinical findings will be described at each time point, like the number of prevalent conditions as well of new diseases detected, diagnoses of sub-clinical diseases,

For peer review only - http://bmjopen.bmj.com/site/about/guidelines.xhtml

relapse of the original tumour, late effects and diagnostic measurements. The results will be adjusted for multiple testing.

For the health economic evaluation, we will calculate incremental cost-effectiveness ratios (ICERs) for different outcomes. The estimated benefits of the intervention in terms of empowerment (HEIQ), quality of life (SF-36, EQ-5D-5L, ICECAP-A), and other outcomes are compared to the additional costs of implementing the PanCareFollowUp Care Intervention. Costs include resources incurred at the level of the hospital and the survivor. At the hospital level, we measure the time of physicians and other hospital staff for tasks related to the clinic visit and the follow-up call, costs for diagnostic and screening tests and other consumables for the clinic visit. At the survivor level, we measure the time investment and travel costs of survivors and relatives or friends, and loss of productive time at the workplace or in education. These costs are investigated separately on each level, hospital and survivor, as well as on an aggregated level. To account for statistical uncertainty in the cost data, we will apply a bootstrap approach using empirical and/or theoretical distributions on different cost positions. Results are displayed in a cost-effectiveness plane. Since there are no uniform ceiling values on ICERs across countries (and for the different outcomes), we will also show cost-effectiveness acceptability curves, which account for statistical uncertainty in the ICERs and in the ceiling values.

In the calculation of ICERs, we will take into account the follow-up of six months, which implies that longer-run effects of PanCareFollowUp Care on outcomes such as survival cannot be measured within the study, and effects on other outcomes such as quality of life may be small. We therefore complement our analysis with a model-based economic evaluation approach using data from this study as well as information from the literature on longer-term effects of follow-up interventions and patient pathways, which will allow us to gain a more comprehensive picture on the cost-effectiveness of PanCareFollowUp Care.

Handling missing data

Automated reminders and phone calls by the clinics are used to ensure that all patients and HCPs complete all questionnaires to minimise the number of missing data. In case of missing data for certain PROMs and PREMs, we will replace missing values with the mean of the remaining items of the scale as recommended by the manuals. In case of other missing data, we will perform sensitivity analyses, i.e. perform the analyses with the complete cases and repeat the analyses with imputed values.

Data management

For peer review only - http://bmjopen.bmj.com/site/about/guidelines.xhtml

A cloud-based Electronic Data Capture platform has been developed by the Danish Cancer Society using Castor EDC (www.castoredc.com). This platform can be accessed by each of the four study sites for data entry. Castor EDC is compliant with all the important regulations regarding research: GDPR, ISO 27001 & ISO 9001 with servers located in the Netherlands including several measures to ensure security, adequacy and veracity of the collected data: regular back-ups (four times per day); personal accounts with individual user rights; audit, data and edit trail of all entered and changed data; and real-time edit checks to identify discrepancies in entered data.

Participating survivors complete their questionnaires directly in Castor EDC through a personalised link they receive by e-mail. Clinical data will be provided by HCPs or retrieved from survivors' medical records and entered into Castor EDC by local data managers according to a data entry instruction manual. All personal and sensitive data collected in the PanCareFollowUp project will be pseudonymised.

After the end of the data collection period, data will be exported from Castor to servers at the Danish Cancer Society. Experienced data managers will perform quality checks, data cleaning, and validation of data collected at the four sites and will set up data for the respective statistical analyses as subsets of the main database, governed by Data Transfer Agreements. The investigators will properly address all the ethical, legal, and safety aspects of the study and comply fully with EU Regulation 2016/679 on the protection of natural persons with regard to the processing of personal data and on the free movement of such data, and repealing Directive 95/46/EC (General Data Protection Regulation).

Ethics and dissemination

This study will be conducted in accordance with the guidelines of Good Clinical Practice by the International Council for Harmonisation of Technical Requirements for Pharmaceuticals for Human Use and the Declaration of Helsinki, written to protect those involved in clinical studies. The study protocol has been reviewed and approved by all relevant ethics committees: Brno, Ethics Committee of St. Anne's University Hospital (13 August 2019); Leuven, Ethics Committee Research University Hospitals Leuven (16 December 2020); Stockholm, Ethics Review Authority Stockholm (26 October 2020); Genoa, N. Liguria Regional Ethics Committee (13 July 2020).

Written informed consent will be obtained from all study participants before enrolment and data collection. An independent ethics advisor from Denmark is available to provide feedback and advice on ethics issues that may arise. An external study steering committee has been appointed to act as

For peer review only

For peer review only - http://bmjopen.bmj.com/site/about/guidelines.xhtml

an advisory capacity with study oversight and external advice. The committee includes a survivor representative, a clinical oncologist, a late effects specialist, an ethicist and a statistician.

Incidental findings based on participants' completion of the questionnaires are unlikely given the nature of the questions, except for one question of the Brief Symptom Inventory-18 on suicidal thoughts. The central data centre and the four study sites will regularly check for any positive answers on this specific question, and inform the health care provider as soon as possible, but within a maximum of two weeks. Worrisome answers at the pre-visit questionnaire will be discussed at the clinic visit. In the post-visit questionnaires, the survivor is informed that he or she can contact their general physician or late effects clinic in case of worrisome symptoms or complaints.

After the project, a Replication Manual will be developed for anyone interested in implementing the PanCareFollowUp Care for adult survivors of childhood cancer. It will include an updated Intervention Manual based on the Care Study results and additional focus groups with project stakeholders after the study closes. The Replication Manual will include all materials required for implementation in different languages and will become freely available through PanCare. PanCareFollowUp is aligned with EC Open Science Initiative, providing open access to all publications, and participates in the H2020 Open Research Data Pilot. The PanCareFollowUp Consortium will ensure that the collected data is findable, accessible, interoperable and reusable (FAIR). A dissemination plan including policy and press releases has been created warranting publications and lay language summaries on the different outcomes collected, to be distributed through the networks of PanCare and several (inter)national childhood cancer organisations. In addition, results will be published in peer-reviewed journals and presented on the project website.

Disclaimer

The material presented and views expressed here are the responsibility of the author(s) only. The EU Commission takes no responsibility for any use made of the information set out.

Declarations

Protocol date and identifier

March 9th 2020, first version.

For peer review only - http://bmjopen.bmj.com/site/about/guidelines.xhtml

For peer review only

May 19th 2020, second version (adjustment in the paragraph about local data storage and transfer to central database).

January 21st 2021, third version (adjustment in the paragraph about data controllership and data processorship).

Protocol amendments

Protocol amendments, if any, will need to be approved by all investigators and are available upon request.

Consent for publication

Not applicable.

Availability of data and materials

Not applicable.

Competing interests

The authors declare that they have no competing interests.

Funding

This work was supported by the European Union's Horizon 2020 research and innovation programme [grant number 824982], and the Swedish Childhood Cancer Fund [grant number EU 2018-0002], and the Italian Ministry of Health [grant number not applicable]. The funding bodies and primary sponsor had no role in the design of the study; in the collection, management, analysis and interpretation of data; in writing of the report; or in the decision to submit the report for publication.

Primary sponsor

Princess Máxima Center for Pediatric Oncology

Heidelberglaan 25

3584 CS Utrecht, the Netherlands

Coordinator and contact for public and scientific queries

Prof Leontien C.M. Kremer (L.C.M.Kremer@prinsesmaximacentrum.nl)

Princess Máxima Center for Pediatric Oncology

For peer review only - http://bmjopen.bmj.com/site/about/guidelines.xhtml

Heidelberglaan 25

3584 CS Utrecht, the Netherlands

+31 88 9 72 72 72

Data monitoring committee

Not applicable, since this intervention is care as usual.

Auditing

Not applicable, since this intervention is care as usual.

Access to data

During the conduct of the Care Study, the study sponsor (Princess Máxima Center for Pediatric Oncology) will act as data controller, whereas the study sites are each joint controllers of the data collected at their own study site, and the Danish Cancer Society will act as data processor. Access to the data is regulated by a Data Processing Agreement between the Princess Máxima Center for Pediatric Oncology and the Danish Cancer Society, and by Study Site Agreements between the Princess Máxima Center for Pediatric Oncology and each of the four study sites. A Data Transfer Agreement between the Princess Máxima Center and specific project partners will govern the transfer of data for purposes of analysis after data collection has been completed.

Individual participant-level data (IPD) sharing

Public access to the full protocol, participant-level dataset and statistical code will be granted upon request, provided that their use is in agreement with the individual informed consent forms and contractual project agreements.

Author contributions

RK, JK, MR and LK contributed to the conception and design of the work and drafted and substantially revised the manuscript. RH, MM, TK, KK, AB, SB, LEF, SE, JFW, RH, AK, JL, GM, RM, KO, HP, SP, KR, RS, MR, AU, CF and LH contributed to the conception and design of the work and critically revised the manuscript. All authors read and approved of the final manuscript.

Acknowledgements

The authors gratefully acknowledge Carina Schneider and Patricia McColgan for organizing pilot tests of the study questionnaires with survivors, and Aoife Moggan, Rory McGrath and Daniel Owens for

For peer review only - http://bmjopen.bmj.com/site/about/guidelines.xhtml

their participation in the pilot test. We acknowledge Anja Krøyer, Thomas Tjørnelund Nielsen and Agnethe Kirstine Møldrup Poulsen from the Childhood Cancer Research Group at the Danish Cancer Society Research Centre, Copenhagen, Denmark for setting up the PanCareFollowUp Care Study Castor database.

For peer review only

For peer review only - http://bmjopen.bmj.com/site/about/guidelines.xhtml

References

1. Gatta G, Botta L, Rossi S, Aareleid T, Bielska-Lasota M, Clavel J, et al. Childhood cancer survival in Europe 1999-2007: results of EUROCARE-5--a population-based study. Lancet Oncol. 2014;15(1):35-47.

2. Vassal G, Schrappe M, Pritchard-Jones K, Arnold F, Bassete L, Biondi A, et al. The SIOPE strategic plan: A European cancer plan for children and adolescents. J Cancer Policy. 2016;8:17-32.

3. Geenen MM, Cardous-Ubbink MC, Kremer LC, van den Bos C, van der Pal HJ, Heinen RC, et al. Medical assessment of adverse health outcomes in long-term survivors of childhood cancer. JAMA. 2007;297(24):2705-15.

4. Bhakta N, Liu Q, Ness KK, Baassiri M, Eissa H, Yeo F, et al. The cumulative burden of surviving childhood cancer: an initial report from the St Jude Lifetime Cohort Study (SJLIFE). Lancet. 2017;390(10112):2569-82.

5. Oeffinger KC, Mertens AC, Sklar CA, Kawashima T, Hudson MM, Meadows AT, et al. Chronic health conditions in adult survivors of childhood cancer. N Engl J Med. 2006;355(15):1572-82.

6. Daniel LC, Wang M, Mulrooney DA, Srivastava DK, Schwartz LA, Edelstein K, et al. Sleep, emotional distress, and physical health in survivors of childhood cancer: A report from the Childhood Cancer Survivor Study. Psychooncology. 2019;28(4):903-12.

7. Zeltzer LK, Lu Q, Leisenring W, Tsao JC, Recklitis C, Armstrong G, et al. Psychosocial outcomes and health-related quality of life in adult childhood cancer survivors: a report from the childhood cancer survivor study. Cancer Epidemiol Biomarkers Prev. 2008;17(2):435-46.

8. Font-Gonzalez A, Feijen E, Geskus RB, Dijkgraaf MGW, van der Pal HJH, Heinen RC, et al. Risk and associated risk factors of hospitalization for specific health problems over time in childhood cancer survivors: a medical record linkage study. Cancer Med. 2017;6(5):1123-34.

9. Steingart RM, Liu JE, Oeffinger KC. Cost-effectiveness of screening for asymptomatic left ventricular dysfunction in childhood cancer survivors. Ann Intern Med. 2014;160(10):731-2.

10. Wong FL, Bhatia S, Landier W, Francisco L, Leisenring W, Hudson MM, et al. Cost-effectiveness of the children's oncology group long-term follow-up screening guidelines for childhood cancer survivors at risk for treatment-related heart failure. Ann Intern Med. 2014;160(10):672-83.

11. Skinner R, Wallace WH, Levitt GA, Group UKCsCSGLE. Long-term follow-up of people who have survived cancer during childhood. Lancet Oncol. 2006;7(6):489-98.

12. Essig S, Skinner R, von der Weid NX, Kuehni CE, Michel G. Follow-up programs for childhood cancer survivors in Europe: a questionnaire survey. PLoS One. 2012;7(12):e53201.

13. Haupt R, Spinetta JJ, Ban I, Barr RD, Beck JD, Byrne J, et al. Long term survivors of childhood cancer: cure and care. The Erice statement. Eur J Cancer. 2007;43(12):1778-80.

For peer review only - http://bmjopen.bmj.com/site/about/guidelines.xhtml

14. Jankovic M, Haupt R, Spinetta JJ, Beck JD, Byrne J, Calaminus G, et al. Long-term survivors of childhood cancer: cure and care-the Erice Statement (2006) revised after 10 years (2016). J Cancer Surviv. 2018;12(5):647-50.

15. Kremer LC, Mulder RL, Oeffinger KC, Bhatia S, Landier W, Levitt G, et al. A worldwide collaboration to harmonize guidelines for the long-term follow-up of childhood and young adult cancer survivors: a report from the International Late Effects of Childhood Cancer Guideline Harmonization Group. Pediatr Blood Cancer. 2013;60(4):543-9.

16. Armenian SH, Hudson MM, Mulder RL, Chen MH, Constine LS, Dwyer M, et al. Recommendations for cardiomyopathy surveillance for survivors of childhood cancer: a report from the International Late Effects of Childhood Cancer Guideline Harmonization Group. Lancet Oncol. 2015;16(3):e123-36.

17. Clemens E, van den Heuvel-Eibrink MM, Mulder RL, Kremer LCM, Hudson MM, Skinner R, et al. Recommendations for ototoxicity surveillance for childhood, adolescent, and young adult cancer survivors: a report from the International Late Effects of Childhood Cancer Guideline Harmonization Group in collaboration with the PanCare Consortium. Lancet Oncol. 2019;20(1):e29-e41.

18. Clement SC, Kremer LCM, Verburg FA, Simmons JH, Goldfarb M, Peeters RP, et al. Balancing the benefits and harms of thyroid cancer surveillance in survivors of Childhood, adolescent and young adult cancer: Recommendations from the international Late Effects of Childhood Cancer Guideline Harmonization Group in collaboration with the PanCareSurFup Consortium. Cancer Treat Rev. 2018;63:28-39.

19. Skinner R, Mulder RL, Kremer LC, Hudson MM, Constine LS, Bardi E, et al. Recommendations for gonadotoxicity surveillance in male childhood, adolescent, and young adult cancer survivors: a report from the International Late Effects of Childhood Cancer Guideline Harmonization Group in collaboration with the PanCareSurFup Consortium. Lancet Oncol. 2017;18(2):e75-e90.

20. van Dorp W, Mulder RL, Kremer LC, Hudson MM, van den Heuvel-Eibrink MM, van den Berg MH, et al. Recommendations for Premature Ovarian Insufficiency Surveillance for Female Survivors of Childhood, Adolescent, and Young Adult Cancer: A Report From the International Late Effects of Childhood Cancer Guideline Harmonization Group in Collaboration With the PanCareSurFup Consortium. J Clin Oncol. 2016;34(28):3440-50.

21. van der Kooi ALF, Mulder RL, Hudson MM, Kremer LCM, Skinner R, Constine LS, et al. Counseling and surveillance of obstetrical risks for female childhood, adolescent, and young adult cancer survivors: recommendations from the International Late Effects of Childhood Cancer Guideline Harmonization Group. Am J Obstet Gynecol. 2020.

For peer review only

For peer review only - http://bmjopen.bmj.com/site/about/guidelines.xhtml

22. Christen S, Roser K, Mulder RL, Ilic A, Lie HC, Loonen JJ, et al. Recommendations for the surveillance of cancer-related fatigue in childhood, adolescent, and young adult cancer survivors: a report from the International Late Effects of Childhood Cancer Guideline Harmonization Group. J Cancer Surviv. 2020.

23. Mulder RL, Hudson MM, Bhatia S, Landier W, Levitt G, Constine LS, et al. Updated Breast Cancer Surveillance Recommendations for Female Survivors of Childhood, Adolescent, and Young Adult Cancer From the International Guideline Harmonization Group. J Clin Oncol. 2020:JCO2000562.

24. Michel G, Mulder RL, van der Pal HJH, Skinner R, Bardi E, Brown MC, et al. Evidence-based recommendations for the organization of long-term follow-up care for childhood and adolescent cancer survivors: a report from the PanCareSurFup Guidelines Working Group. J Cancer Surviv. 2019;13(5):759-72.

25. Mulder RL, van der Pal HJH, Levitt GA, Skinner R, Kremer LCM, Brown MC, et al. Transition guidelines: An important step in the future care for childhood cancer survivors. A comprehensive definition as groundwork. Eur J Cancer. 2016;54:64-8.

26. Loonen JJ, Blijlevens NM, Prins J, Dona DJ, Den Hartogh J, Senden T, et al. Cancer Survivorship Care: Person Centered Care in a Multidisciplinary Shared Care Model. Int J Integr Care. 2018;18(1):4.

27. Dutch Childhood Oncology Group. Guidelines for follow-up in survivors of childhood cancer 5 years after diagnosis. 2010. Available at: https://www.skion.nl/workspace/uploads/richtlijn_follow-up_na_kinderkanker_deel_1_1.pdf. Accessed 17 Dec 2021.

28. van Kalsbeek RJ, van der Pal HJH, Hjorth L, Winther JF, Michel G, Haupt R, et al. The European multistakeholder PanCareFollowUp project: novel, person-centred survivorship care to improve care quality, effectiveness, cost-effectiveness and accessibility for cancer survivors and caregivers. Eur J Cancer. 2021;153:74-85.

29. van Kalsbeek RJ, Mulder RL, Haupt R, Muraca M, Hjorth L, Follin C, et al. The PanCareFollowUp Care Intervention: A European harmonised approach to person-centred guideline-based survivorship care after childhood, adolescent and young adult cancer. Eur J Cancer. 2022;162:34-44.

30. Haupt R, Essiaf S, Dellacasa C, Ronckers CM, Caruso S, Sugden E, et al. The 'Survivorship Passport' for childhood cancer survivors. Eur J Cancer. 2018;102:69-81.

31. Osborne RH, Elsworth GR, Whitfield K. The Health Education Impact Questionnaire (heiQ): an outcomes and evaluation measure for patient education and self-management interventions for people with chronic conditions. Patient Educ Couns. 2007;66(2):192-201.

32. Luttrell C, Quiroz S, Scrutton C, Bird K. Working Paper 308: Understanding and operationalising empowerment. 2009. Available at: https://cdn.odi.org/media/documents/5500.pdf. Accessed 17 Dec 2021.

For peer review only - http://bmjopen.bmj.com/site/about/guidelines.xhtml

33. European Patients' Froum. Background Brief: Patient Empowerment. 2015. Available at: https://www.eu-patient.eu/globalassets/campaign-patient-empowerment/epf_briefing_patientempowerment_2015.pdf. Accessed 17 Dec 2021.

34. Glasgow RE, Harden SM, Gaglio B, Rabin B, Smith ML, Porter GC, et al. RE-AIM Planning and Evaluation Framework: Adapting to New Science and Practice With a 20-Year Review. Front Public Health. 2019;7:64.

35. Maunsell E, Lauzier S, Brunet J, Pelletier S, Osborne RH, Campbell HS. Health-related empowerment in cancer: validity of scales from the Health Education Impact Questionnaire. Cancer. 2014;120(20):3228-36.

For peer review only

For peer review only - http://bmjopen.bmj.com/site/about/guidelines.xhtml

Figure legends

Figure 1. Overview of all patient-reported outcome measures (PROMs) and experience measures (PREMs), clinical outcomes, feasibility outcomes and health economic outcomes used in the Care Study. Outcomes that are specific for males or females are indicated as such between brackets. For the clinical outcomes, it is indicated whether they are assessed through a diagnostic test according to the guidelines (d), Survivor Questionnaire (q), or both (d+q). Other clinical outcomes are assessed through medical history and/or physical examination. Abbreviations: BPI = Brief Pain Inventory, BSI-18 = Brief Symptom Inventory-18, CD-RISC 25 = Connor-Davidson Resilience Scale (25 items), ET = Emotion Thermometer, HCP = health care provider, HEIQ = health education impact questionnaire, HRQoL = health-related quality of life, ICECAP-A = ICEpop CAPability measure for Adults, LH/FSH = luteinising hormone/follicle-stimulating hormone, PROMIS = Patient-Reported Outcomes Measurement Information System, PCL-5 = PTSD Checklist for DSM-5, QoL = quality of life, Satisfaction Qx = Satisfaction questionnaire by Blaauwbroek et al, SCP = Survivorship Care Plan, SDM-Q-9 = 9-item shared decision-making questionnaire (patient perspective), SF-36 = Short Form-36 (36 items, version 1), SQx = Survivor Questionnaire (part of the PanCareFollowUp Care Intervention), TSH = thyroid-stimulating hormone, SDM-Q-Doc = 9-item Shared Decision-Making Questionnaire (HCP perspective).

References: [a] Brunet J., Lauzier S., Campbell H.S., Fillion L., Osborne R.H., Maunsell E., Measurement invariance of English and French Health Education Impact Questionnaire (heiQ) empowerment scales validated for cancer. Qual Life Res, 2015. 24(10): p. 2375-84.; Osborne R.H., Elsworth G.R., Whitfield K., The Health Education Impact Questionnaire (heiQ): an outcomes and evaluation measure for patient education and self-management interventions for people with chronic conditions. Patient Educ Couns, 2007. 66(2): p. 192-201.

[b] Blaauwbroek, R., Tuinier, W., Jong, B. M., Kamps, W. A., & Postma, A. (2008). Shared care by paediatric oncologists and family doctors for long-term follow-up of adult childhood cancer survivors: a pilot study. Lancet Oncology, 9(3), 232-238. DOI: 10.1016/S1470-2045(08)70034-2.

[c] Kriston L., Scholl I., Holzel L., Simon D., Loh A., Harter M., The 9-item Shared Decision Making Questionnaire (SDM-Q-9). Development and psychometric properties in a primary care sample. Patient Educ Couns, 2010. 80(1): p. 94-9.; Rodenburg-Vandenbussche S., Pieterse A.H., Kroonenberg P.M., Scholl I., van der Weijden T., Luyten G.P., et al., Dutch Translation and Psychometric Testing of the 9-Item Shared Decision Making Questionnaire (SDM-Q-9) and Shared Decision Making Questionnaire-Physician Version (SDM-Q-Doc) in Primary and Secondary Care. PLoS One, 2015. 10(7): p. e0132158.

For peer review only - http://bmjopen.bmj.com/site/about/guidelines.xhtml

[d] Connor K.M., Davidson J.R., Development of a new resilience scale: the Connor-Davidson Resilience Scale (CD-RISC). Depress Anxiety, 2003. 18(2): p. 76-82.

[e] EQ-5D-5L: Herdman, M., et al., Development and preliminary testing of the new five-level version of EQ-5D (EQ-5D-5L). Qual Life Res, 2011. 20(10): p. 1727-36.; SF-36: Ware J.E., Jr., Gandek B., Overview of the SF-36 Health Survey and the International Quality of Life Assessment (IQOLA) Project. J Clin Epidemiol, 1998. 51(11): p. 903-12.; ICECAP-A: Al-Janabi, H., T.N. Flynn, and J. Coast, Development of a self-report measure of capability wellbeing for adults: the ICECAP-A. Qual Life Res, 2012. 21(1): p. 167-76.

[f] Derogatis, L.R., BSI 18 - Brief Symptom Inventory 18 - Administration, Scoring, and Procedures Manual. 2000: NCS Pearson Inc.

[g] Blevins, C.A., et al., The Posttraumatic Stress Disorder Checklist for DSM-5 (PCL-5): Development and Initial Psychometric Evaluation. J Trauma Stress, 2015. 28(6): p. 489-98.

[h] Mitchell, A.J., et al., Can the Distress Thermometer be improved by additional mood domains? Part I. Initial validation of the Emotion Thermometers tool. Psychooncology, 2010. 19(2): p. 125-33., Mitchell, A.J., et al., Can the Distress Thermometer be improved by additional mood domains? Part II. What is the optimal combination of Emotion Thermometers? Psychooncology, 2010. 19(2): p. 134-40.

[i] Christen, S. et al., Recommendations for the Surveillance of Cancer-Related Fatigue in Childhood, Adolescent and Young Adult Cancer Survivors: A Report from the International Late Effects of Childhood Cancer Guideline Harmonization Group and the PanCare Guidelines Group. Journal of Cancer Survivorship. 2020;14(6):923-938.

[j] Bingham Iii, C.O., et al., PROMIS Fatigue short forms are reliable and valid in adults with rheumatoid arthritis. J Patient Rep Outcomes, 2019. 3(1): p. 14.

[k] Cleeland, C.S. and K.M. Ryan, Pain assessment: global use of the Brief Pain Inventory. Ann Acad Med Singapore, 1994. 23(2): p. 129-38.

Figure 2. Flowchart of data collection after inclusion of an eligible survivor. Abbreviations: HCP = health care provider, PREMS = patient-reported experience measures, PROMS = patient-reported outcome measures, T1 = time point 1, T2 = time point 2, T3 = time point 3, T4 = time point 4, T5 = time point 5. The boxes describe for each time point the timing of data collection, the person providing data (survivor, HCP or both), the data collection instruments (Survivor Questionnaire, Treatment Summary or T1-T5 study questionnaire) and the types of outcomes collected. Depicted in blue is data collected for care, and in purple for research purposes.

For peer review only

For peer review only - http://bmjopen.bmj.com/site/about/guidelines.xhtml

**PROMs or PREMs: survivors**	Premature ovarian insufficiency (females) (d)	Neurocognitive problems: language	Telangiectasias of the eye
Empowerment (HEIQ)[a] (primary outcome)	Testosterone deficiency (males) (d)	Neurocognitive problems: memory	Xerophthalmia
Patient satisfaction (Satisfaction Qx)[b]	TSH deficiency (d)	Neurocognitive problems: motor integration	**Feasibility outcomes: survivor**
Shared decision-making (SDM-Q-9)[c]	*Gastro-intestinal*	Neurocognitive problems: processing speed	Received care according to SCP
Resilience (CD-RISC 25)[d]	Bowel obstruction	Psychological distress (q)	Success of communication
HRQoL (EQ-5D-5L, SF-36, ICECAP-A)[e]	Chronic enterocolitis	Stress-related mental disorder	Missing information
Psychological distress (BSI-18)[f]	Gastro-intestinal strictures or fistula	Suicidal ideation (q)	*Italian study site only:* Use of and satisfaction with SurPass
Post-traumatic stress symptoms (PCL-5)[g]	*Hepato-biliary*	Unemployment (q)	
Distress (ET)[h]	Cholelithiasis	*Renal and urinary tract*	**Feasibility outcomes: HCP (per clinic)**
Fatigue (SQx + PROMIS Fatigue – Short Form 8a +)[i,j]	Hepatobiliary dysfunction (d)	Bladder fibrosis	No. of eligible survivors invited
Pain (BPI)[k]	Hepatocellular liver injury (stage 1) (d)	Dysfunctional voiding (q)	No. of participating survivors per time point
Lifestyle (SQx)	Iron overload (d)	Glomerular kidney dysfunction (d)	No. of non-responders
Social functioning (SQx)	Liver cirrhosis	Haemorrhagic cystitis	Reasons for non-response
**Clinical outcomes**	Liver fibrosis	Hydronephrosis	No. of drop-outs per time point
Auditory	Liver synthetic dysfunction (d)	Tubular kidney dysfunction (d)	Reasons for drop-outs per time point
Hearing loss (d + q)	*Immunological*	Vesicoureteral reflux	Composition of multidisciplinary team
Tinnitus (q)	Spleen problems (overwhelming infections)	*Reproductive*	Use of the SCP
Cardiac	*Musculoskeletal*	Impaired fertility (q)	Reasons for non-use of SCP, if applicable
Arrhythmia (d + q)	Craniofacial growth problems	Impaired spermatogenesis (males) (d + q)	Shared decision making (HCP perspective; SDM-Q-Doc)[c]
Cardiomyopathy (d)	Osteonecrosis	Low birth weight of offspring (females) (q)	Extent to which SCP of participating survivors has been implemented and reasons for deviating
Pericardial disease (d)	Reduced bone mineral density (d)	Miscarriage (females) (q)	
Valvular heart disease (d)	Spine kyphosis	Physical sexual dysfunction (males) (q)	*Italian study site only:* no. of SurPasses delivered, recommendation brochures given and SurPasses shared with physicians, SurPass user statistics
Dental	Spine scoliosis	Premature birth of offspring (females) (q)	
Dental caries	*Neurological*	*Respiratory*	
Dental developmental problems	Cavernomas	Pulmonary dysfunction (d + q)	**Health economic outcomes: survivor**
Xerostomia (q)	Cerebrovascular accidents	*Subsequent neoplasm*	Time investment of survivor (preparation for clinic visit, travel, total time in clinic, follow-up appointments)
Dermatologic	Neurogenic bladder	Subsequent neoplasm (benign or malignant) (d + q)	
Alopecia	Neurogenic bowel	*Vascular*	Time investment of relatives (travel, total time in clinic, follow-up appointments)
Endocrine	Optic chiasm neuropathy	Aneurysms	
ACTH deficiency (d)	Pain (q)	Asymptomatic coronary artery disease	Travel costs of survivor and relatives
Amenorrhea (females) (q)	Peripheral motor neuropathy (q)	Carotid artery disease	Other extra costs for survivor and relatives
Central precocious puberty (d)	Peripheral sensory neuropathy (q)	Dyslipidaemia (d)	Loss of time for survivor and relatives at paid work or in education
Diabetes mellitus (d)	*Psychosocial and neurocognitive*	Hypertension	
Failure in pubertal progression	Adjustment difficulties	*Visual*	**Health economic outcomes: HCP**
Growth hormone deficiency (d)	Anxiety (q)	Cataract	Time investment of HCP and other staff tasks related to clinic visit (preparation, clinic visit, tasks following clinic visit, follow-up call)
Hyperthyroidism (d)	Behavioural problems	Chronic painful eye	
Hypothyroidism (peripheral) (d)	Fatigue (q)	Glaucoma	
Impaired glucose metabolism (d)	Low educational status (q)	Keratitis	Costs for diagnostic and screening tests
LH/FSH deficiency (d)	Neurocognitive problems: academics	Lacrimal duct atrophy	Costs for other consumables for clinic visit
Obesity	Neurocognitive problems: attention	Maculopathy	
Overweight	Neurocognitive problems: executive function	Papillopathy	
Premature menopause (females) (d)	Neurocognitive problems: intelligence	Retinopathy	

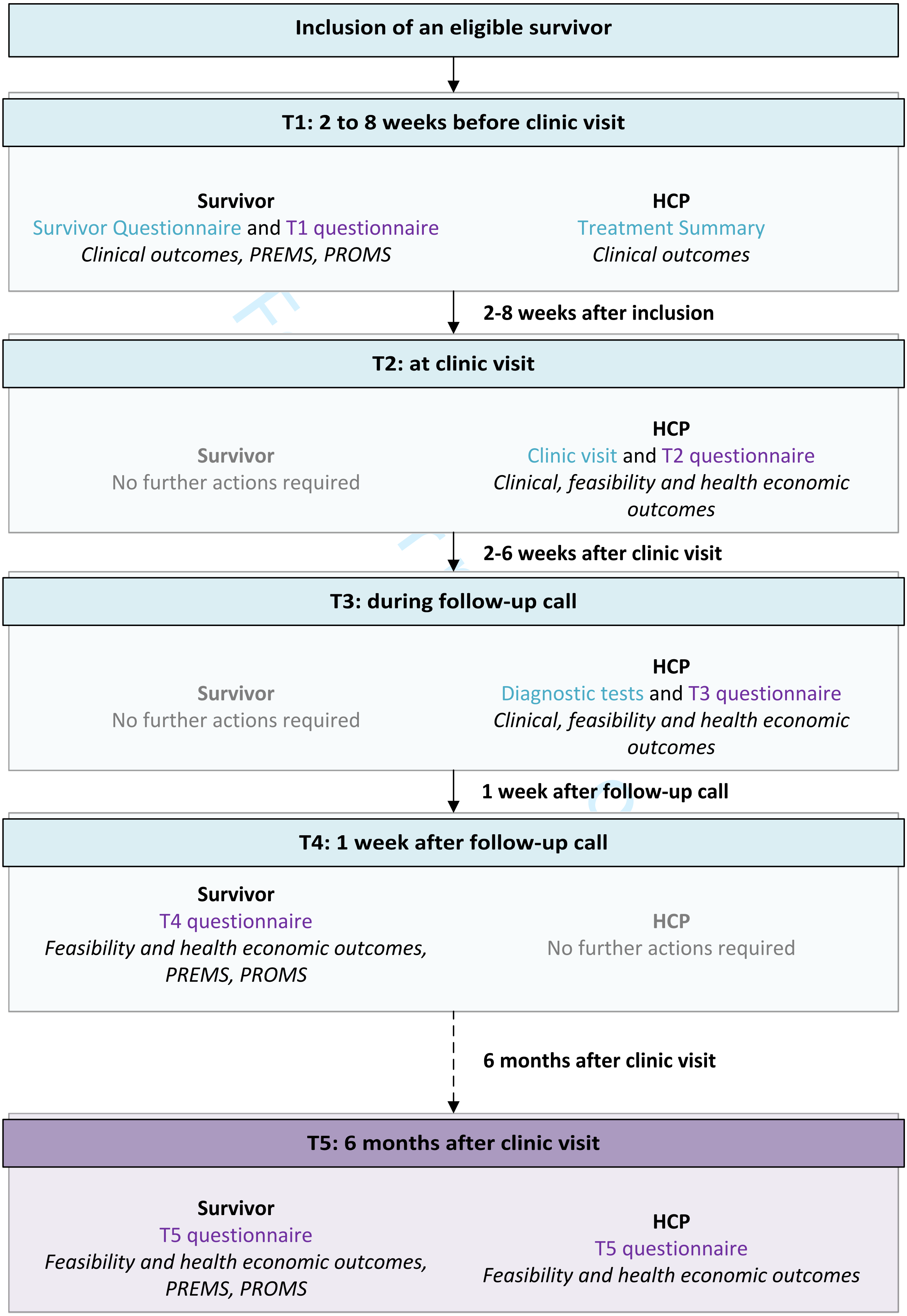
Inclusion of an eligible survivor
T1: 2 to 8 weeks before clinic visit
Survivor
Survivor Questionnaire and T1 questionnaire
Clinical outcomes, PREMS, PROMS
HCP
Treatment Summary
Clinical outcomes
2-8 weeks after inclusion
T2: at clinic visit
Survivor
No further actions required
HCP
Clinic visit and T2 questionnaire
Clinical, feasibility and health economic outcomes
2-6 weeks after clinic visit
T3: during follow-up call
Survivor
No further actions required
HCP
Diagnostic tests and T3 questionnaire
Clinical, feasibility and health economic outcomes
1 week after follow-up call
T4: 1 week after follow-up call
Survivor
T4 questionnaire
Feasibility and health economic outcomes, PREMS, PROMS
HCP
No further actions required
6 months after clinic visit
T5: 6 months after clinic visit
Survivor
T5 questionnaire
Feasibility and health economic outcomes, PREMS, PROMS
HCP
T5 questionnaire
Feasibility and health economic outcomes

Appendix A: Recruitment strategy of each study site

Sweden starts with inviting a random sample, prioritising survivors who are lost to follow-up or have not visited the study site in the past five years, and might invite survivors who received care more recently depending on the recruitment rate among the initial population.

Italy starts with inviting survivors who already have a scheduled appointment at their clinic, but who had not already received the Survivorship Passport, and are resident in the Liguria region. They will invite 350 to 400 survivors to be able to include 200 survivors. They will subsequently recruit scheduled survivors resident in other regions, and if the number is still insufficient, they will actively invite other survivors to the clinic.

The Czech Republic starts with selecting a random sample of 250 survivors from the clinic's database whom they will gradually invite over the recruitment period. If more survivors need to be invited to reach the inclusion aim within the recruitment period, they will invite survivors who have a scheduled appointment at their clinic and who meet the study inclusion criteria

Belgium starts to invite, in alphabetical order the survivors of 18 year and older with a primary cancer diagnosis with a date of diagnosis in or before 1990, regardless of whether or not they already received some long-term follow-up. Simultaneously, 20 survivors who were scheduled for a clinic visit in March and April 2021 have also been invited to participate in this study. In the second wave, they will invite the survivors with a diagnosis in 1990-2000 in alphabetic order. And, if needed, the survivors diagnosed in 2001-2020, again in alphabetic order.

For peer review only

STANDARD PROTOCOL ITEMS: RECOMMENDATIONS FOR INTERVENTIONAL TRIALS

SPIRIT 2013 Checklist: Recommended items to address in a clinical trial protocol and related documents*

Section/item	Item No	Description	Addressed on page number
**Administrative information**			
Title	1	Descriptive title identifying the study design, population, interventions, and, if applicable, trial acronym	1
Trial registration	2a	Trial identifier and registry name. If not yet registered, name of intended registry	6
	2b	All items from the World Health Organization Trial Registration Data Set	1-25
Protocol version	3	Date and version identifier	8, 17, 18
Funding	4	Sources and types of financial, material, and other support	18
Roles and responsibilities	5a	Names, affiliations, and roles of protocol contributors	1,2
	5b	Name and contact information for the trial sponsor	18
	5c	Role of study sponsor and funders, if any, in study design; collection, management, analysis, and interpretation of data; writing of the report; and the decision to submit the report for publication, including whether they will have ultimate authority over any of these activities	18
	5d	Composition, roles, and responsibilities of the coordinating centre, steering committee, endpoint adjudication committee, data management team, and other individuals or groups overseeing the trial, if applicable (see Item 21a for data monitoring committee)	18, 19

For peer review only

**Introduction**			
Background and rationale	6a	Description of research question and justification for undertaking the trial, including summary of relevant studies (published and unpublished) examining benefits and harms for each intervention	7, 8
	6b	Explanation for choice of comparators	8, 11, 12
Objectives	7	Specific objectives or hypotheses	8
Trial design	8	Description of trial design including type of trial (eg, parallel group, crossover, factorial, single group), allocation ratio, and framework (eg, superiority, equivalence, noninferiority, exploratory)	9
**Methods: Participants, interventions, and outcomes**			
Study setting	9	Description of study settings (eg, community clinic, academic hospital) and list of countries where data will be collected. Reference to where list of study sites can be obtained	9
Eligibility criteria	10	Inclusion and exclusion criteria for participants. If applicable, eligibility criteria for study centres and individuals who will perform the interventions (eg, surgeons, psychotherapists)	9
Interventions	11a	Interventions for each group with sufficient detail to allow replication, including how and when they will be administered	8, 9, 10, ref 29
	11b	Criteria for discontinuing or modifying allocated interventions for a given trial participant (eg, drug dose change in response to harms, participant request, or improving/worsening disease)	10
	11c	Strategies to improve adherence to intervention protocols, and any procedures for monitoring adherence (eg, drug tablet return, laboratory tests)	N/A
	11d	Relevant concomitant care and interventions that are permitted or prohibited during the trial	N/A
Outcomes	12	Primary, secondary, and other outcomes, including the specific measurement variable (eg, systolic blood pressure), analysis metric (eg, change from baseline, final value, time to event), method of aggregation (eg, median, proportion), and time point for each outcome. Explanation of the clinical relevance of chosen efficacy and harm outcomes is strongly recommended	11, 12
Participant timeline	13	Time schedule of enrolment, interventions (including any run-ins and washouts), assessments, and visits for participants. A schematic diagram is highly recommended (see Figure)	10, Fig 2

For peer review only - http://bmjopen.bmj.com/site/about/guidelines.xhtml

Sample size	14	Estimated number of participants needed to achieve study objectives and how it was determined, including clinical and statistical assumptions supporting any sample size calculations	9, 10, 13, 14
Recruitment	15	Strategies for achieving adequate participant enrolment to reach target sample size	10, 15, App A
**Methods: Assignment of interventions (for controlled trials)**			
Allocation:			
Sequence generation	16a	Method of generating the allocation sequence (eg, computer-generated random numbers), and list of any factors for stratification. To reduce predictability of a random sequence, details of any planned restriction (eg, blocking) should be provided in a separate document that is unavailable to those who enrol participants or assign interventions	N/A
Allocation concealment mechanism	16b	Mechanism of implementing the allocation sequence (eg, central telephone; sequentially numbered, opaque, sealed envelopes), describing any steps to conceal the sequence until interventions are assigned	N/A
Implementation	16c	Who will generate the allocation sequence, who will enrol participants, and who will assign participants to interventions	N/A
Blinding (masking)	17a	Who will be blinded after assignment to interventions (eg, trial participants, care providers, outcome assessors, data analysts), and how	N/A
	17b	If blinded, circumstances under which unblinding is permissible, and procedure for revealing a participant's allocated intervention during the trial	N/A
**Methods: Data collection, management, and analysis**			
Data collection methods	18a	Plans for assessment and collection of outcome, baseline, and other trial data, including any related processes to promote data quality (eg, duplicate measurements, training of assessors) and a description of study instruments (eg, questionnaires, laboratory tests) along with their reliability and validity, if known. Reference to where data collection forms can be found, if not in the protocol	14
	18b	Plans to promote participant retention and complete follow-up, including list of any outcome data to be collected for participants who discontinue or deviate from intervention protocols	10, 15, App A

For peer review only - http://bmjopen.bmj.com/site/about/guidelines.xhtml

Data management	19	Plans for data entry, coding, security, and storage, including any related processes to promote data quality (eg, double data entry; range checks for data values). Reference to where details of data management procedures can be found, if not in the protocol	15, 16
Statistical methods	20a	Statistical methods for analysing primary and secondary outcomes. Reference to where other details of the statistical analysis plan can be found, if not in the protocol	14, 15
	20b	Methods for any additional analyses (eg, subgroup and adjusted analyses)	14
	20c	Definition of analysis population relating to protocol non-adherence (eg, as randomised analysis), and any statistical methods to handle missing data (eg, multiple imputation)	14
**Methods: Monitoring**			
Data monitoring	21a	Composition of data monitoring committee (DMC); summary of its role and reporting structure; statement of whether it is independent from the sponsor and competing interests; and reference to where further details about its charter can be found, if not in the protocol. Alternatively, an explanation of why a DMC is not needed	19
	21b	Description of any interim analyses and stopping guidelines, including who will have access to these interim results and make the final decision to terminate the trial	N/A
Harms	22	Plans for collecting, assessing, reporting, and managing solicited and spontaneously reported adverse events and other unintended effects of trial interventions or trial conduct	17
Auditing	23	Frequency and procedures for auditing trial conduct, if any, and whether the process will be independent from investigators and the sponsor	19
**Ethics and dissemination**			
Research ethics approval	24	Plans for seeking research ethics committee/institutional review board (REC/IRB) approval	16
Protocol amendments	25	Plans for communicating important protocol modifications (eg, changes to eligibility criteria, outcomes, analyses) to relevant parties (eg, investigators, REC/IRBs, trial participants, trial registries, journals, regulators)	18

For peer review only - http://bmjopen.bmj.com/site/about/guidelines.xhtml

Consent or assent	26a	Who will obtain informed consent or assent from potential trial participants or authorised surrogates, and how (see Item 32)	10, 16
	26b	Additional consent provisions for collection and use of participant data and biological specimens in ancillary studies, if applicable	N/A
Confidentiality	27	How personal information about potential and enrolled participants will be collected, shared, and maintained in order to protect confidentiality before, during, and after the trial	15, 16, 19
Declaration of interests	28	Financial and other competing interests for principal investigators for the overall trial and each study site	18
Access to data	29	Statement of who will have access to the final trial dataset, and disclosure of contractual agreements that limit such access for investigators	19
Ancillary and post-trial care	30	Provisions, if any, for ancillary and post-trial care, and for compensation to those who suffer harm from trial participation	17
Dissemination policy	31a	Plans for investigators and sponsor to communicate trial results to participants, healthcare professionals, the public, and other relevant groups (eg, via publication, reporting in results databases, or other data sharing arrangements), including any publication restrictions	17
	31b	Authorship eligibility guidelines and any intended use of professional writers	19
	31c	Plans, if any, for granting public access to the full protocol, participant-level dataset, and statistical code	19
**Appendices**			
Informed consent materials	32	Model consent form and other related documentation given to participants and authorised surrogates	Submitted separately
Biological specimens	33	Plans for collection, laboratory evaluation, and storage of biological specimens for genetic or molecular analysis in the current trial and for future use in ancillary studies, if applicable	N/A

*It is strongly recommended that this checklist be read in conjunction with the SPIRIT 2013 Explanation & Elaboration for important clarification on the items. Amendments to the protocol should be tracked and dated. The SPIRIT checklist is copyrighted by the SPIRIT Group under the Creative Commons "Attribution-NonCommercial-NoDerivs 3.0 Unported" license.

For peer review only

BMJ Open

Evaluating the feasibility and cost-effectiveness of implementing person-centred follow-up care for childhood cancer survivors in four European countries: the PanCareFollowUp Care prospective cohort study protocol

Journal:	*BMJ Open*
Manuscript ID	bmjopen-2022-063134.R1
Article Type:	Protocol
Date Submitted by the Author:	27-Sep-2022
Complete List of Authors:	van Kalsbeek, Rebecca; Princess Maxima Center for Pediatric Oncology<br>Korevaar, J; Netherlands Institute for Health Services Research (NIVEL)<br>Rijken, Mieke; Netherlands Institute for Health Services Research (NIVEL); University of Eastern Finland - Kuopio Campus, Department of Health and Social Care Management<br>Haupt, Riccardo; IRCCS Istituto Giannina Gaslini, Epidemiology and Biostatistics Unit and DOPO clinic<br>Muraca, Monica; IRCCS Istituto Giannina Gaslini, Epidemiology and Biostatistics Unit and DOPO clinic<br>Kepák, Tomáš; Masaryk University Faculty of Medicine, International Clinical Research Centre (FNUSA-ICRC) at St. Anne's University Hospital<br>Kepakova, Katerina; Masaryk University Faculty of Medicine, International Clinical Research Centre (FNUSA-ICRC) at St. Anne's University Hospital<br>Blondeel, Anne; European Society for Pediatric Oncology (SIOP Europe)<br>Boes, Stefan; University of Lucerne, Department of Health Sciences and Medicine<br>Frederiksen, Line Elmerdahl; Danish Cancer Society Research Center, Childhood Cancer Research Group<br>Essiaf, Samira; European Society for Pediatric Oncology (SIOP Europe)<br>Winther, Jeanette; Danish Cancer Society Research Center, Childhood Cancer Research Group; Aarhus Universitet, Department of Clinical Medicine and Faculty of Health<br>Hermens, Rosella; Radboudumc, Scientific Institute for Quality of Healthcare (IQ Healthcare)<br>Kienesberger, Anita; Childhood Cancer International Europe<br>Loonen, Jacqueline; Radboudumc, Department of Hematology<br>Michel, Gisela; University of Lucerne, Department of Health Sciences and Medicine<br>Mulder, Renée; Princess Maxima Center for Pediatric Oncology<br>O'Brien, Kylie; Pintail, Limited<br>van der Pal, Helena; Princess Maxima Center for Pediatric Oncology; PanCare<br>Pluijm, Saskia ; Princess Maxima Center for Pediatric Oncology<br>Roser, Katharina; University of Lucerne, Department of Health Sciences and Medicine<br>Skinner, Roderick; Newcastle University Centre for Cancer, Wolfson Childhood Cancer Research Centre; Great North Children's Hospital,

	Royal Victoria Infirmary<br>Renard, Marleen; KU Leuven University Hospitals Leuven, Department of Paediatric Haematology and Oncology<br>Uyttebroeck, Anne; KU Leuven University Hospitals Leuven, Department of Paediatric Haematology and Oncology<br>Follin, Cecilia; Skane University Hospital, Department of Clinical Sciences Lund, Oncology<br>Hjorth, Lars; Skane University Hospital, Department of Clinical Sciences Lund, Paediatrics<br>Kremer, Leontien; Princess Maxima Center for Pediatric Oncology; Emma Childrens' Hospital UMC, Department of Paediatrics
**Primary Subject Heading**:	Oncology
Secondary Subject Heading:	Health policy, Oncology, Paediatrics, Patient-centred medicine, Research methods
Keywords:	International health services < HEALTH SERVICES ADMINISTRATION & MANAGEMENT, Health economics < HEALTH SERVICES ADMINISTRATION & MANAGEMENT, Paediatric oncology < ONCOLOGY, Paediatric oncology < PAEDIATRICS, Organisation of health services < HEALTH SERVICES ADMINISTRATION & MANAGEMENT

SCHOLARONE™ Manuscripts

Evaluating the feasibility and cost-effectiveness of implementing person-centred follow-up care for childhood cancer survivors in four European countries: the PanCareFollowUp Care prospective cohort study protocol

Rebecca J. van Kalsbeek[a], Joke C. Korevaar[b], Mieke Rijken[b,c], Riccardo Haupt[d], Monica Muraca[d], Tomas Kepak[e], Katerina Kepakova[e], Anne Blondeel[f], Stefan Boes[g], Line Elmerdahl Frederiksen[h], Samira Essiaf[f], Jeanette Falck Winther[h,i], Rosella P.M.G. Hermens[j], Anita Kienesberger[k], Jacqueline J. Loonen[l], Gisela Michel[g], Renée L. Mulder[a], Kylie O'Brien[m], Helena J.H. van der Pal[a,n], Saskia M.F. Pluijm[a], Katharina Roser[g], Roderick Skinner[o,p,q], Marleen Renard[r], Anne Uyttebroeck[r], Cecilia Follin[s], Lars Hjorth[t], Leontien C. M. Kremer[a,u,v]

Affiliations:

[a] Princess Máxima Centre for Paediatric Oncology, Heidelberglaan 25, 3584 CS, Utrecht, the Netherlands

[b] Netherlands Institute for Health Services Research (Nivel), P.O. Box 1568, 3500 BN Utrecht, the Netherlands

[c] University of Eastern Finland, Department of Health and Social Management, P.O. Box 1627, FI-70211 Kuopio, Finland

[d] Epidemiology and Biostatistics Unit and DOPO clinic, IRCCS Istituto Giannina Gaslini, Via G. Gaslini, 5, 16147 Genoa, Italy

[e] International Clinical Research Centre (FNUSA-ICRC) at St. Anne's University Hospital, Masaryk University, Pekařská 53, Brno 656 91, Czech Republic

[f] European Society for Paediatric Oncology, c/o BLSI, Clos Chapelle-aux-Champs 30, Bte 1.30.30, BE-1200 Brussels, Belgium

[g] University of Lucerne, Department of Health Sciences and Medicine, Frohburgstrasse 3, P.O. Box 4466, 6002 Lucerne, Switzerland

[h] Childhood Cancer Research Group, Danish Cancer Society Research Centre, Strandboulevarden 49, 2100 Copenhagen, Denmark

[i] Department of Clinical Medicine, Faculty of Health, Aarhus University and Aarhus University Hospital, Palle Juul-Jensens Boulevard 82, 8200 Aarhus, Denmark

For peer review only - http://bmjopen.bmj.com/site/about/guidelines.xhtml

For peer review only

[j] Radboud University Medical Centre, Radboud Institute for Health Sciences, Scientific Institute for Quality of Healthcare (IQ Healthcare), Geert Grooteplein 21, 6525 EZ, Nijmegen, the Netherlands

[k] Childhood Cancer International Europe, Servitengasse 5/16, 1090 Vienna, Austria

[l] Radboud University Medical Centre, Radboud Institute for Health Sciences, Department of Hematology, Geert Grooteplein Zuid 10, 6525 GA, Nijmegen, the Netherlands

[m] Pintail, Limited, 77 Springhill Avenue, Blackrock, Dublin, Ireland

[n] PanCare, Jacobus Bellamylaan 16, 1401 AZ Bussum, the Netherlands

[o] Newcastle University Centre for Cancer, Wolfson Childhood Cancer Research Centre, Herschel Building, Brewery Lane, Newcastle upon Tyne, NE1 7RU, United Kingdom

[p] Great North Children's Hospital, Royal Victoria Infirmary, Queen Victoria Road, Newcastle upon Tyne, NE1 4 LP, United Kingdom

[q] Translational and Clinical Research Institute, Wolfson Childhood Cancer Research Centre, Herschel Building, Brewery Lane, Newcastle upon Tyne, NE1 7RU, United Kingdom

[r] Department of Oncology, Paediatric Oncology, KU Leuven, Department of Paediatric Haematology and Oncology, University Hospitals Leuven, Herestraat 49, 3000 Leuven, Belgium

[s] Lund University, Skane University Hospital, Department of Clinical Sciences Lund, Oncology, Lasarettsgatan 40, 221 85 Lund, Sweden

[t] Lund University, Skane University Hospital, Department of Clinical Sciences Lund, Paediatrics, Lasarettsgatan 40, 221 85 Lund, Sweden

[u] Department of Paediatrics, Emma Children's Hospital, Amsterdam UMC, Meibergdreef 9, 1105 AZ Amsterdam, the Netherlands

[v] Faculty of Medicine, Utrecht University and Utrecht Medical Centre, Universiteitsweg 98, 3584 CG Utrecht, the Netherlands

Corresponding author: Rebecca J. van Kalsbeek
R.J.vanKalsbeek@prinsesmaximacentrum.nl
Princess Máxima Center for Pediatric Oncology
Heidelberglaan 25
3584 CS Utrecht, The Netherlands
+ 31 88 972 72 72

For peer review only

For peer review only - http://bmjopen.bmj.com/site/about/guidelines.xhtml

**Public title:**	Impact of person-centred follow-up care for European childhood cancer survivors: PanCareFollowUp Care

**Keywords:**	Childhood cancer; survivorship; person-centered care; shared decision-making; empowerment; quality of life; clinical trial protocol; cohort study; prospective study; multicenter study, cost effectiveness

**Word count:**	4161 words (including Box 1)
**Abstract word count:**	297 words
**Tables:**	1
**Figures:**	2
**Appendices:**	1

For peer review only

List of abbreviations:

FAIR = Findable, accessible, interoperable and reusable

GCP = Good Clinical Practice

HCP(s) = Health care provider(s)

ICER(s) = Incremental cost-effectiveness ratio(s)

IGHG = International Late Effects of Childhood Cancer Guideline Harmonization Group

PanCare = Pan-European Network for Care of Survivors after Childhood and Adolescent Cancer

PROM = Patient-reported outcome measure

PREM = Patient-reported experience measure

RE-AIM = Reach, Effectiveness, Adoption, Implementation and Maintenance

SD = Standard deviation

SurPass = Survivorship Passport

T1-5 = Time points 1-5

For peer review only

Abstract

Introduction – Long-term survival after childhood cancer often comes at the expense of late, adverse health conditions. However, survivorship care is often not available for adult survivors in Europe. The PanCareFollowUp Consortium therefore developed the PanCareFollowUp Care Intervention, an innovative person-centred survivorship care model based on experiences in the Netherlands. This paper describes the protocol of the prospective cohort study (Care Study) to evaluate the feasibility and the health economic, clinical and patient-reported outcomes of implementing PanCareFollowUp Care as usual care in four European countries.

Methods and analysis – In this prospective, longitudinal cohort study with at least six months of follow-up, 800 childhood cancer survivors will receive the PanCareFollowUp Care Intervention across four study sites in Belgium, Czech Republic, Italy and Sweden, representing different health care systems. The PanCareFollowUp Care Intervention will be evaluated according to the RE-AIM (Reach, Effectiveness, Adoption, Implementation and Maintenance) framework. Clinical and research data are collected through questionnaires, a clinic visit for multiple medical assessments and a follow-up call. The primary outcome is empowerment, assessed with the Health Education Impact Questionnaire (HEIQ). A central data centre will perform quality checks, data cleaning, data validation, and provide support in data analysis. Multilevel models will be used for repeated outcome measures, with subgroup analysis, e.g. by centre, attained age, sex or diagnosis.

Ethics and dissemination - This study will be conducted in accordance with the guidelines of Good Clinical Practice and the Declaration of Helsinki. The study protocol has been reviewed and approved by all relevant ethics committees. The evidence and insights gained by this study will be summarised in a Replication Manual, also including the tools required to implement the PanCareFollowUp Care Intervention in other countries. This Replication Manual will become freely available through PanCare and will be disseminated through policy and press releases.

Trial registration - NL8918, registered at the Netherlands Trial Register at 24 September 2020, https://www.trialregister.nl/trial/8918.

For peer review only - http://bmjopen.bmj.com/site/about/guidelines.xhtml

Article summary

Strengths and limitations of this study

- The PanCareFollowUp Care Study is designed and conducted together with survivor representatives, ensuring the outcome measures are relevant for survivors and that PanCareFollowUp Care meets their needs and expectations.
- We include survivors from four different European countries, representing a variety of health care systems across Europe; and their experiences are used to improve the PanCareFollowUp Care Intervention before free distribution of the materials in a Replication Manual.
- The PanCareFollowUp Care Intervention is evaluated in a real life setting with a minimal number of exclusion criteria.
- Since the Care Study has a limited follow-up time, a model-based economic evaluation will complement the analyses.
- Participants are their own controls and effects are evaluated as changes from baseline within an individual or institution.

For peer review only

For peer review only - http://bmjopen.bmj.com/site/about/guidelines.xhtml

Introduction

Over the last decades, five-year survival rates of childhood cancer in Europe have increased substantially, from 30% in the 1970s to 80% in the early 2000s (1). Today, the European population of childhood cancer survivors, estimated at minimally 300,000, is rising by about 12,000 per year (2). Yet, many survivors not only experience the burden of previous cancer diagnosis, but also face treatment-related late effects (3, 4). These may become apparent years or even decades after finishing therapy (5) and might have a significant adverse impact on quality of life (6, 7). Moreover, the transition from paediatric to adult health care settings often lacks continuity. As a result, many adults who survived childhood cancer have increased health care use and experience problems in participation, which generate a substantial burden for survivors and societies in general (8-10). Early detection of new health conditions is essential as it could prevent further harm (11). This requires lifelong survivorship care with frequent adaptations of the follow-up plan.

Currently, only one third of European paediatric oncology clinics provide survivorship care to adult survivors of childhood cancer (12). In 2006, an international group of paediatric oncologists, psychologists, nurses, epidemiologists, survivors and their parents agreed in the Erice statement that has recently been updated and reconfirmed (13, 14) that follow-up care should be available and accessible for all survivors throughout their lifespan.

In the past decade, international evidence-based clinical practice guidelines have been developed to support early detection and treatment of (a)symptomatic late effects, including those developed by the International Late Effects of Childhood Cancer Guideline Harmonization Group (IGHG), sometimes in collaboration with the PanCareSurFup project (15-23). A European model of care guideline is published and guidelines for the transition from childhood to adult health care settings and health promotion are currently being developed (24, 25). Yet, implementation lags behind. Recently, a person-centred approach for survivorship care for adult survivors has been implemented in Nijmegen, the Netherlands (26). All Dutch survivors of childhood cancer are invited for follow-up care by a long-term follow-up care clinic, in which multidisciplinary teams deliver person-centred care based on contemporary surveillance guidelines (27). The first positive effects of this person-centred approach have been reported (24, 26). The next step is to validate this person-centred approach for survivorship care in other countries.

The PanCareFollowUp Consortium, established in 2018, is a unique multidisciplinary European collaboration between 14 project partners from 10 European countries, including survivors (www.pancarefollowup.eu) (28). The aim of the consortium is to improve the quality of life for

For peer review only - http://bmjopen.bmj.com/site/about/guidelines.xhtml

survivors of childhood, adolescent and young adult cancer by bringing evidence-based, person-centred care to clinical practice. The PanCareFollowUp Consortium has developed two interventions: 1) a person-centred and guideline-based model of survivorship care (PanCareFollowUp Care Intervention) (see Box 1) (29) and 2) an eHealth lifestyle coaching model (PanCareFollowUp Lifestyle Intervention). The protocol of the first intervention is described in this paper (version 3, January 21st, 2021), the protocol of the second one will be described separately. Both will be evaluated within the PanCareFollowUp project. The consortium published a Care Intervention Manual that contains instructions and tools required for implementing the PanCareFollowUp Care Intervention. At the project end, Replication Manuals that contain the instructions and tools required for implementation of the PanCareFollowUp Interventions will be freely distributed.

The overall aim of the PanCareFollowUp Care Study is to evaluate the feasibility and (cost-) effectiveness of implementing PanCareFollowUp Care as usual care for adult survivors of childhood cancer in four study sites in four European countries. Four objectives have been formulated: 1) To what extent is implementing PanCareFollowUp Care in the participating study sites feasible?; 2) What are the patient-reported experiences and outcomes, including survivor empowerment, of PanCareFollowUp Care and how do they change?; 3) What is the number and nature of pre-existing and new clinical events detected by PanCareFollowUp Care among participating survivors?; and 4) What is the cost-effectiveness and cost-utility of implementing PanCareFollowUp Care relative to usual care from the perspective of survivors, health care providers (HCPs), and society at large?

Box 1: The PanCareFollowUp Care Intervention

The PanCareFollowUp Care Intervention is based on a person-centred care model (26) that aims to meet the physical, psychological and social needs of (adult) survivors of childhood cancer through shared decision-making about prevention, surveillance and treatment options. The Care Intervention consists of three steps:

a) *Preparation of the clinic visit by both the survivor and the health care provider (HCP).* The survivor provides information about their health, wellbeing, needs and preferences by completing the PanCareFollowUp Survivor Questionnaire. The HCP prepares a Treatment Summary describing the childhood cancer treatment that the survivor has received, reviews the relevant surveillance recommendations and the PanCareFollowUp Survivor Questionnaire provided by the survivor, and thereupon prepares the Standard Survivorship Care Plan.

For peer review only - http://bmjopen.bmj.com/site/about/guidelines.xhtml

b) *Clinic visit including tailored follow-up care.* After obtaining a medical history and performing a physical examination, the survivor and HCP jointly discuss the results of the Survivor Questionnaire, and the Standard Survivorship Care Plan. Together, they agree on a plan for diagnostic tests and potential referral if needed, based on surveillance guidelines or clinical indication. Based on these shared decisions, as well as potential test results, the HCP creates a Draft Individualised Survivorship Care Plan and provides tailored health education.

c) *Follow-up call.* The survivor and HCP discuss the test results and the preferred model of care for future follow-up care. The results of these shared decisions are incorporated in the final Individualised Survivorship Care Plan, that the survivor may share with other HCPs.

The PanCareFollowUp Care Intervention ends after co-creation and delivery of the Individualised Survivorship Care Plan. Survivors will thereafter remain under surveillance either at or under the guidance of their clinic, frequently adjusting their Individualised Survivorship Care Plan when needed.

Methods and analysis

Study population, setting and recruitment

Survivors fulfil the inclusion criteria if they are or have been: diagnosed with cancer before the age of 19 years; treated or registered at one of the four study sites; treated with chemotherapy and/or radiation therapy for childhood cancer with or without surgery; at least five years from primary cancer diagnosis; at least one year off treatment (also applying to treatment of subsequent benign or malignant neoplasms or relapse of the primary cancer); and currently at least 16 years of age.

Exclusion criteria consist of: being unable to complete the study questionnaires because of severe neurocognitive sequelae or insufficient understanding of the language used (even with help from another person); or having previously received complete follow-up care that is similar to the care as described in the PanCareFollowUp Care Intervention Manual (Box 1).

This international prospective cohort study will be conducted at four study sites located in four European countries: Belgium, Czech Republic, Italy, and Sweden. All sites currently provide long-term follow-up care, either within a paediatric (Belgium, Italy) or adult (Czech Republic, Sweden) oncology centre, using a set of (inter)national guidelines and protocols. Each study site aims to include 200 survivors who complete the study. With an estimated non-response and early drop-out (informed consent signed, but no actual participation in the study) of 40 to 50% based on previous

For peer review only - http://bmjopen.bmj.com/site/about/guidelines.xhtml

experience and an estimated late drop-out (at any point after completing the T1 questionnaire) of 5-10% during the study, approximately 350 to 400 survivors will therefore be invited at each site. To assess the feasibility of this recruitment strategy, each centre screened their respective registries and estimated a total of 5,944 eligible survivors.

Each study site developed a recruitment strategy within the prerequisites of this study, that fits best within their own logistics (Appendix A). Selected survivors will be invited by an invitation letter, an invitation e-mail or by phone (depending on the usual procedure at each study site), and receive an information sheet, including contact details for additional information, and an informed consent form. Reasons for non-participation can be provided. One option of the pre-set reasons is 'not participating because the questionnaires are being provided via internet'. In this case, the study site may decide to offer the option for paper questionnaires. Survivors who give informed consent but do not respond to the first questionnaire, even after reminders, are considered early drop-outs and will be excluded from the study, as essential data about these survivors will not be available. The first participant was enrolled in February 2021, and at 1 March 2022 456 participants were enrolled and completed the clinic visit. The estimated last inclusion is on 30 September 2022, with last data collection 31 May 2023.

Participating survivors can withdraw from the study at any time if they wish. They are not obliged to provide a reason for withdrawal, although it will be asked and recorded if available. To assess representativeness of the final study sample, the four centres will provide aggregated data about their total eligible population of survivors including population distributions of gender, current age, age at diagnosis, type of cancer and distance to the late effects clinic. This will be compared to the distributions among the included survivors per clinic.

During recruitment and data collection, careful monitoring of enrolment, (non-)response, reasons for non-response and early and late drop-out will be performed by the four study sites in close collaboration with the central data centre at the Danish Cancer Society Research Centre.

Intervention

Survivors of childhood cancer who receive PanCareFollowUp Care (i.e., care in accordance with the PanCareFollowUp Care Intervention Manual and as outlined in Box 1) will be followed up until six months after the clinic visit. The implementation of person-centred care in this project is facilitated by a narrated Powerpoint and an on-site workshop for all HCPs involved in the study. An add-on study investigating the feasibility of delivering PanCareFollowUp Care using the digital Survivorship

For peer review only - http://bmjopen.bmj.com/site/about/guidelines.xhtml

Passport (SurPass) tool (30) will be conducted at the Italian clinic, where SurPass is already implemented.

Primary and secondary outcomes

This study uses a variety of outcomes to answer the four research objectives (Figure 1). These are measured from time point 1 (T1) before the clinic visit until T5 at six months after the clinic visit (Figure 2). Outcomes are provided by survivors and HCPs through questionnaires, a clinic visit and diagnostic tests.

1) To what extent is implementing PanCareFollowUp Care in the participating study sites feasible?

Feasibility of implementation is of major importance to ensure sustainability of the PanCareFollowUp Care Intervention. Therefore, feasibility indicators measured by questionnaires among survivors and HCPs as well as an evaluation of barriers and facilitators are included to inform about the experiences of implementing PanCareFollowUp Care (Figure 2). Items include, among others, drop-outs at different time-points, use of and experiences with the Survivorship Care Plan, and shared-decision making (Figure 1).

2) What are the experiences and outcomes as reported by participating survivors receiving PanCareFollowUp Care?

The primary outcome for this study is empowerment measured by the Health Education Impact Questionnaire (HEIQ) (31). Empowerment has been defined by the EU Joint Action on Patient Safety and Quality of Care as a ‘multidimensional process that helps people gain control over their own lives and increase their capacity to act on issues that they themselves define as important’, a definition adapted from Lutrell et al. (32, 33). Empowerment has been selected as the primary outcome because childhood cancer survivors encounter several transition moments starting from diagnosis, after which a greater responsibility for their own health and care is required. It is essential that survivors receive the support they need to manage and advocate for their needs. Moreover, empowerment is important to manage future health problems. We have included six of the eight scales of the HEIQ relevant to cancer survivors in our study (Social integration and support, Health service navigation, Constructive attitudes and approaches, Skill and technique acquisition, Emotional distress, Self-Monitoring and insight). The HEIQ has previously been used in cancer patient and survivor populations (34-36). It allows to calculate a mean for each scale indicating higher or lower empowerment in the respective domain within a participant compared to the baseline assessment.

For peer review only - http://bmjopen.bmj.com/site/about/guidelines.xhtml

Secondary outcomes consist of a variety of patient-reported experiences and outcomes (PREMs and PROMs), such as satisfaction and quality of life (Figure 1).

3) What is the number and nature of pre-existing and new clinical events detected by PanCareFollowUp Care among participating survivors?

Clinical outcomes are outcomes of symptoms and diseases and have been defined based on published or almost published guidelines of the IGHG and the PanCareFollowUp Recommendations. A total of 116 clinical outcomes were defined, which reflects the wide range of late effects that survivors may encounter affecting both physical health and psychosocial wellbeing (Figure 1). Clinical outcomes include past and current medical history, are collected through survivor self-report in the Survivor Questionnaire (with verification at the clinic visit), and physician-report in the Treatment Summary, after the clinic visit and after potential diagnostic tests (Figure 2). The number and range of pre-existing and newly detected health problems (symptomatic and asymptomatic) per survivor will be described, including the results of clinical examinations (e.g. echocardiogram or blood tests).

4) What is the cost-effectiveness and cost-utility of implementing PanCareFollowUp Care relative to usual care from the perspective of survivors, HCPs, and society at large?

The cost-effectiveness and cost-utility of the care model will be determined by using health economic outcomes (Figure 1). These reflect the time, time off work and monetary investments made by the survivor, accompanying relatives or friends, the HCP and other staff in relation to the clinic visit while receiving or providing PanCareFollowUp Care, and are collected using questionnaires (Figure 2). We do not take costs outside the clinic visit into account, i.e., costs related to possible (follow-up) primary care physician visits, mental health services, or referrals to other specialists outside the clinical setting. Costs related to the clinic visit, as associated with PanCareFollowUp Care, are compared to potential benefits measured in terms of PREMs and PROMs.

An overall evaluation of implementing the PanCareFollowUp Care Intervention will be performed throughout the project according to the RE-AIM (Reach, Effectiveness, Adoption, Implementation and Maintenance) framework to assess the impact (www.re-aim.org) (37) (Table 1).

Table 1. RE-AIM framework applied to the PanCareFollowUp Care Intervention.

Components	Related outcomes/actions in the Care Study
Reach	• No. and proportion of participants vs. non-responders

For peer review only - http://bmjopen.bmj.com/site/about/guidelines.xhtml

	• Representativeness of participating survivors[a] (comparison of distribution: gender, current age, age at diagnosis and type of cancer)<br>• Reasons for (non-)participation
Effectiveness/efficacy	• Main outcome empowerment[a]<br>• Patient-reported outcome and experience measures, and clinical, feasibility and health economic outcomes[a]
Adoption[b]	• Multidisciplinarity of HCPs involved<br>• Recruitment rate<br>• Barriers and facilitators for recruitment
Implementation[b]	• Use of SCP and reasons for non-use<br>• Adaptations made to the PanCareFollowUp Care Intervention or implementation strategy<br>• Time and costs of PanCareFollowUp Care for survivors and HCPs<br>• Barriers and facilitators for implementation
Maintenance	• Replication Manual including updated implementation and recruitment strategy, publicly available for current and new centres<br>• Overview of requirements for study sites to make the PanCareFollowUp Care Intervention routine care

Abbreviations: HCPs = health care providers, SCP = Survivorship Care Plan. [a] Comparisons will be made according to subgroups of gender, current age, age at diagnosis and type of cancer. [b] This information will be collected at each study site separately.

For peer review only

Patient and public involvement

Survivor representatives from Childhood Cancer International-Europe are included in the project as members of the PanCareFollowUp Consortium (28). They are involved throughout the project and reach out to their respective national and international networks when needed. Survivors were involved in setting the research agenda by writing the grant application and the study protocol, developing and reviewing the PanCareFollowUp Care Intervention materials, evaluating the study questionnaires, monitoring the progress of the PanCareFollowUp Care Study and creating awareness on social media (29). They helped consider ways to mitigate the burden of completing the study questionnaires or remembering the childhood cancer history for participants. After the end of data

For peer review only - http://bmjopen.bmj.com/site/about/guidelines.xhtml

collection, survivor representatives will be involved in the interpretation of the study results and dissemination to participants, survivor networks and the general public.

Power calculation

We aim to include 200 participants at each of the four study sites (total n=800). The primary outcome measure is change in empowerment between T1 and T5 as measured by the HEIQ (34). We use six constructs (cancer version including five constructs plus one additional construct, namely self-monitoring and insight) with mean scores ranging from 2.9 (standard deviation (SD): 0.64) to 3.2 (SD: 0.48). Taking the construct with the largest SD (thus needing the highest number of participants to demonstrate a statistically significant change), limiting it to a single study site, with a 2-sided α of 0.05, a power of 80%, we will need 200 participants to identify an effect size of 0.2 given a mean score of 2.9 (SD: 0.64). That is enough power to demonstrate a small to medium effect. The actual power is larger since we ignored measuring empowerment repeatedly, having four centres (800 patients instead of 200) and using constructs with smaller SDs.

Data collection

Data will be collected from participating survivors as well as from their HCPs at five time points (T1-T5) during a follow-up period of six to eight months (Figure 2). We will use data collected in the context of care delivery, and combine it with additional data collected specifically for research purposes. For the latter, there are three data collection moments for survivors and four for HCPs. These time points are linked to the structure of the PanCareFollowUp Care Intervention, which consists of three steps: 1) Preparation of the clinic visit by survivor and HCP (corresponding with T1), 2) Clinic visit (corresponding with T2), and 3) Follow-up call (two to four weeks after T2, corresponding with T3). Thereafter, there is data collection at 1 week after the follow-up call (T4) and 6 months after the clinic visit (T5).

The main data collection instruments consist of the PanCareFollowUp Survivor Questionnaire (care), the Treatment Summary (care), medical history, physical examinations and diagnostic tests during and after the clinic visit (care), and additional online study questionnaires for survivors and HCPs (research). The Survivor Questionnaire and Treatment Summary are available through open access (29). The English versions of the study questionnaires for survivors have been pretested by three survivors, whereas the English questionnaires for HCPs have been pretested with at least two HCPs in each centre before the start of the data collection. The questionnaires for survivors have subsequently been translated to the local languages of the study sites, i.e. Czech, Dutch, Italian and Swedish.

For peer review only - http://bmjopen.bmj.com/site/about/guidelines.xhtml

Statistical analysis

For analysing outcomes measured multiple times, like the primary outcome, we will analyse multilevel models for repeated measures applying a fixed effect to control for study site. Next, we will perform subgroup analyses for relevant groups by including interaction terms. These subgroups will be identified based on the literature combined with knowledge from professionals. The final selection will be determined during the study, however, possible subgroups may be distinguished according to centre, sex, time since cancer diagnosis, treatment type, or distance to late effects clinic. The models will be adjusted for confounders, which will be identified during the study based on the literature and expert opinion. Clinical findings will be described at each time point, like the number of prevalent conditions as well as new diseases detected, diagnoses of sub-clinical diseases, relapse of the original tumour, late effects and diagnostic measurements. The results will be adjusted for multiple testing.

For the health economic evaluation, we will calculate incremental cost-effectiveness ratios (ICERs) for different outcomes. The estimated benefits of the intervention in terms of empowerment (HEIQ), quality of life (Short-Form 36 (SF-36), EQ-5D-5L, ICEpop CAPability measure for Adults (ICECAP-A)), and other outcomes are compared to the additional costs of implementing the PanCareFollowUp Care Intervention. Costs include resources incurred at the level of the hospital and the survivor. At the hospital level, we measure the time of physicians and other hospital staff for tasks related to the clinic visit and the follow-up call, costs for diagnostic and screening tests and other consumables for the clinic visit. At the survivor level, we measure the time investment and travel costs of survivors and relatives or friends, and loss of productive time at the workplace or in education. These costs are investigated separately on each level, hospital and survivor, as well as on an aggregated level. To account for statistical uncertainty in the cost data, we will apply a bootstrap approach using empirical and/or theoretical distributions on different cost positions. Results are displayed in a cost-effectiveness plane. Since there are no uniform ceiling values on ICERs across countries (and for the different outcomes), we will also show cost-effectiveness acceptability curves, which account for statistical uncertainty in the ICERs and in the ceiling values.

The calculation of ICERs needs to be interpreted in light of the relatively short follow-up period of six months within the study. This implies that the cost-effectiveness analysis mainly focuses on short-run effects, while longer-run effects of PanCareFollowUp Care on outcomes such as survival cannot be measured within the study. Moreover, effects on other outcomes such as quality of life may be small. In order to provide information about the potential medium- to lon-run effects, we will complement our analysis with a model-based economic evaluation approach using data from this

For peer review only - http://bmjopen.bmj.com/site/about/guidelines.xhtml

study as well as information from the literature on longer-term effects of follow-up interventions and patient pathways, as well as related cost estimations. This will allow us to gain a more comprehensive picture on the cost-effectiveness of PanCareFollowUp Care.

Handling missing data

Automated reminders and phone calls by the clinics are used to ensure that all patients and HCPs complete all questionnaires to minimise the number of missing data. In case of missing data for certain PROMs and PREMs, we will replace missing values with the mean of the remaining items of the scale as recommended by the manuals. In case of other missing data, we will perform sensitivity analyses, i.e. perform the analyses with the complete cases and repeat the analyses with imputed values.

Data management

A cloud-based Electronic Data Capture platform has been developed by the Danish Cancer Society using Castor EDC (www.castoredc.com). This platform can be accessed by each of the four study sites for data entry. Castor EDC is compliant with all the important regulations regarding research: GDPR, ISO 27001 & ISO 9001 with servers located in the Netherlands including several measures to ensure security, adequacy and veracity of the collected data: regular back-ups (four times per day); personal accounts with individual user rights; audit, data and edit trail of all entered and changed data; and real-time edit checks to identify discrepancies in entered data.

Participating survivors complete their questionnaires directly in Castor EDC through a personalised link they receive by e-mail. Clinical data will be provided by HCPs or retrieved from survivors' medical records and entered into Castor EDC by local data managers according to a data entry instruction manual. All personal and sensitive data collected in the PanCareFollowUp project will be pseudonymised.

After the end of the data collection period, data will be exported from Castor to servers at the Danish Cancer Society. Experienced data managers will perform quality checks, data cleaning, and validation of data collected at the four sites and will set up data for the respective statistical analyses as subsets of the main database, governed by Data Transfer Agreements. The investigators will properly address all the ethical, legal, and safety aspects of the study and comply fully with EU Regulation 2016/679 on the protection of natural persons with regard to the processing of personal data and on the free movement of such data, and repealing Directive 95/46/EC (General Data Protection Regulation).

For peer review only - http://bmjopen.bmj.com/site/about/guidelines.xhtml

Ethics and dissemination

This study will be conducted in accordance with the guidelines of Good Clinical Practice by the International Council for Harmonisation of Technical Requirements for Pharmaceuticals for Human Use and the Declaration of Helsinki, written to protect those involved in clinical studies. The study protocol has been reviewed and approved by all relevant ethics committees: Brno, Ethics Committee of St. Anne's University Hospital (13 August 2019); Leuven, Ethics Committee Research University Hospitals Leuven (16 December 2020); Stockholm, Ethics Review Authority Stockholm (26 October 2020); Genoa, N. Liguria Regional Ethics Committee (13 July 2020).

Written informed consent will be obtained from all study participants before enrolment and data collection. An independent ethics advisor from Denmark is available to provide feedback and advice on ethics issues that may arise. An external study steering committee has been appointed to act as an advisory capacity with study oversight and external advice. The committee includes a survivor representative, a clinical oncologist, a late effects specialist, an ethicist and a statistician.

Incidental findings based on participants' completion of the questionnaires are unlikely given the nature of the questions, except for one question of the Brief Symptom Inventory-18 on suicidal thoughts. The central data centre and the four study sites will regularly check for any positive answers on this specific question, and inform the HCP as soon as possible, but within a maximum of two weeks. Worrisome answers at the pre-visit questionnaire will be discussed at the clinic visit. In the post-visit questionnaires, the survivor is informed that he or she can contact their general physician or late effects clinic in case of worrisome symptoms or complaints.

After the project, a Replication Manual will be developed for anyone interested in implementing the PanCareFollowUp Care for adult survivors of childhood cancer. It will include an updated Intervention Manual based on the Care Study results and additional focus groups with project stakeholders after the study closes. The Replication Manual will include all materials required for implementation in different languages and will become freely available through PanCare. PanCareFollowUp is aligned with EC Open Science Initiative, providing open access to all publications, and participates in the H2020 Open Research Data Pilot. The PanCareFollowUp Consortium will ensure that the collected data is findable, accessible, interoperable and reusable (FAIR). A dissemination plan including policy and press releases has been created warranting publications and lay language summaries on the different outcomes collected, to be distributed through the networks of PanCare and several (inter)national childhood cancer organisations. In addition, results will be published in peer-reviewed journals and presented on the project website.

For peer review only - http://bmjopen.bmj.com/site/about/guidelines.xhtml

Disclaimer

The material presented and views expressed here are the responsibility of the author(s) only. The EU Commission takes no responsibility for any use made of the information set out.

Declarations

Protocol date and identifier

March 9th 2020, first version.

May 19th 2020, second version (adjustment in the paragraph about local data storage and transfer to central database).

January 21st 2021, third version (adjustment in the paragraph about data controllership and data processorship).

Protocol amendments

Protocol amendments, if any, will need to be approved by all investigators and are available upon request.

Consent for publication

Not applicable.

Availability of data and materials

Not applicable.

Competing interests

The authors declare that they have no competing interests.

Funding

This work was supported by the European Union's Horizon 2020 research and innovation programme [grant number 824982], and the Swedish Childhood Cancer Fund [grant number EU 2018-0002], and

For peer review only

For peer review only - http://bmjopen.bmj.com/site/about/guidelines.xhtml

the Italian Ministry of Health [grant number not applicable]. The funding bodies and primary sponsor had no role in the design of the study; in the collection, management, analysis and interpretation of data; in writing of the report; or in the decision to submit the report for publication.

Primary sponsor

Princess Máxima Center for Pediatric Oncology
Heidelberglaan 25
3584 CS Utrecht, the Netherlands

Coordinator and contact for public and scientific queries

Prof Leontien C.M. Kremer (L.C.M.Kremer@prinsesmaximacentrum.nl)
Princess Máxima Center for Pediatric Oncology
Heidelberglaan 25
3584 CS Utrecht, the Netherlands
+31 88 9 72 72 72

Data monitoring committee

Not applicable, since this intervention is care as usual.

Auditing

Not applicable, since this intervention is care as usual.

Access to data

During the conduct of the Care Study, the study sponsor (Princess Máxima Center for Pediatric Oncology) will act as data controller, whereas the study sites are each joint controllers of the data collected at their own study site, and the Danish Cancer Society will act as data processor. Access to the data is regulated by a Data Processing Agreement between the Princess Máxima Center for Pediatric Oncology and the Danish Cancer Society, and by Study Site Agreements between the Princess Máxima Center for Pediatric Oncology and each of the four study sites. A Data Transfer Agreement between the Princess Máxima Center and specific project partners will govern the transfer of data for purposes of analysis after data collection has been completed.

Individual participant-level data (IPD) sharing

For peer review only - http://bmjopen.bmj.com/site/about/guidelines.xhtml

Public access to the full protocol, participant-level dataset and statistical code will be granted upon request, provided that their use is in agreement with the individual informed consent forms and contractual project agreements.

Author contributions

RK, JK, MR and LK contributed to the conception and design of the work and drafted and substantially revised the manuscript. RH, MM, TK, KK, AB, SB, LEF, SE, JFW, RH, AK, JL, GM, RM, KO, HP, SP, KR, RS, MR, AU, CF and LH contributed to the conception and design of the work and critically revised the manuscript. All authors read and approved of the final manuscript.

Acknowledgements

The authors gratefully acknowledge Carina Schneider and Patricia McColgan for organizing pilot tests of the study questionnaires with survivors, and Aoife Moggan, Rory McGrath and Daniel Owens for their participation in the pilot test. We acknowledge Anja Krøyer, Thomas Tjørnelund Nielsen and Agnethe Kirstine Møldrup Poulsen from the Childhood Cancer Research Group at the Danish Cancer Society Research Centre, Copenhagen, Denmark for setting up the PanCareFollowUp Care Study Castor database.

For peer review only

References

1. Gatta G, Botta L, Rossi S, Aareleid T, Bielska-Lasota M, Clavel J, et al. Childhood cancer survival in Europe 1999-2007: results of EUROCARE-5--a population-based study. Lancet Oncol. 2014;15(1):35-47.

2. Vassal G, Schrappe M, Pritchard-Jones K, Arnold F, Bassete L, Biondi A, et al. The SIOPE strategic plan: A European cancer plan for children and adolescents. J Cancer Policy. 2016;8:17-32.

3. Geenen MM, Cardous-Ubbink MC, Kremer LC, van den Bos C, van der Pal HJ, Heinen RC, et al. Medical assessment of adverse health outcomes in long-term survivors of childhood cancer. JAMA. 2007;297(24):2705-15.

4. Bhakta N, Liu Q, Ness KK, Baassiri M, Eissa H, Yeo F, et al. The cumulative burden of surviving childhood cancer: an initial report from the St Jude Lifetime Cohort Study (SJLIFE). Lancet. 2017;390(10112):2569-82.

5. Oeffinger KC, Mertens AC, Sklar CA, Kawashima T, Hudson MM, Meadows AT, et al. Chronic health conditions in adult survivors of childhood cancer. N Engl J Med. 2006;355(15):1572-82.

6. Daniel LC, Wang M, Mulrooney DA, Srivastava DK, Schwartz LA, Edelstein K, et al. Sleep, emotional distress, and physical health in survivors of childhood cancer: A report from the Childhood Cancer Survivor Study. Psychooncology. 2019;28(4):903-12.

7. Zeltzer LK, Lu Q, Leisenring W, Tsao JC, Recklitis C, Armstrong G, et al. Psychosocial outcomes and health-related quality of life in adult childhood cancer survivors: a report from the childhood cancer survivor study. Cancer Epidemiol Biomarkers Prev. 2008;17(2):435-46.

8. Font-Gonzalez A, Feijen E, Geskus RB, Dijkgraaf MGW, van der Pal HJH, Heinen RC, et al. Risk and associated risk factors of hospitalization for specific health problems over time in childhood cancer survivors: a medical record linkage study. Cancer Med. 2017;6(5):1123-34.

9. Steingart RM, Liu JE, Oeffinger KC. Cost-effectiveness of screening for asymptomatic left ventricular dysfunction in childhood cancer survivors. Ann Intern Med. 2014;160(10):731-2.

10. Wong FL, Bhatia S, Landier W, Francisco L, Leisenring W, Hudson MM, et al. Cost-effectiveness of the children's oncology group long-term follow-up screening guidelines for childhood cancer survivors at risk for treatment-related heart failure. Ann Intern Med. 2014;160(10):672-83.

11. Skinner R, Wallace WH, Levitt GA, Group UKCsCSGLE. Long-term follow-up of people who have survived cancer during childhood. Lancet Oncol. 2006;7(6):489-98.

12. Essig S, Skinner R, von der Weid NX, Kuehni CE, Michel G. Follow-up programs for childhood cancer survivors in Europe: a questionnaire survey. PLoS One. 2012;7(12):e53201.

13. Haupt R, Spinetta JJ, Ban I, Barr RD, Beck JD, Byrne J, et al. Long term survivors of childhood cancer: cure and care. The Erice statement. Eur J Cancer. 2007;43(12):1778-80.

For peer review only - http://bmjopen.bmj.com/site/about/guidelines.xhtml

14. Jankovic M, Haupt R, Spinetta JJ, Beck JD, Byrne J, Calaminus G, et al. Long-term survivors of childhood cancer: cure and care-the Erice Statement (2006) revised after 10 years (2016). J Cancer Surviv. 2018;12(5):647-50.

15. Kremer LC, Mulder RL, Oeffinger KC, Bhatia S, Landier W, Levitt G, et al. A worldwide collaboration to harmonize guidelines for the long-term follow-up of childhood and young adult cancer survivors: a report from the International Late Effects of Childhood Cancer Guideline Harmonization Group. Pediatr Blood Cancer. 2013;60(4):543-9.

16. Armenian SH, Hudson MM, Mulder RL, Chen MH, Constine LS, Dwyer M, et al. Recommendations for cardiomyopathy surveillance for survivors of childhood cancer: a report from the International Late Effects of Childhood Cancer Guideline Harmonization Group. Lancet Oncol. 2015;16(3):e123-36.

17. Clemens E, van den Heuvel-Eibrink MM, Mulder RL, Kremer LCM, Hudson MM, Skinner R, et al. Recommendations for ototoxicity surveillance for childhood, adolescent, and young adult cancer survivors: a report from the International Late Effects of Childhood Cancer Guideline Harmonization Group in collaboration with the PanCare Consortium. Lancet Oncol. 2019;20(1):e29-e41.

18. Clement SC, Kremer LCM, Verburg FA, Simmons JH, Goldfarb M, Peeters RP, et al. Balancing the benefits and harms of thyroid cancer surveillance in survivors of Childhood, adolescent and young adult cancer: Recommendations from the international Late Effects of Childhood Cancer Guideline Harmonization Group in collaboration with the PanCareSurFup Consortium. Cancer Treat Rev. 2018;63:28-39.

19. Skinner R, Mulder RL, Kremer LC, Hudson MM, Constine LS, Bardi E, et al. Recommendations for gonadotoxicity surveillance in male childhood, adolescent, and young adult cancer survivors: a report from the International Late Effects of Childhood Cancer Guideline Harmonization Group in collaboration with the PanCareSurFup Consortium. Lancet Oncol. 2017;18(2):e75-e90.

20. van Dorp W, Mulder RL, Kremer LC, Hudson MM, van den Heuvel-Eibrink MM, van den Berg MH, et al. Recommendations for Premature Ovarian Insufficiency Surveillance for Female Survivors of Childhood, Adolescent, and Young Adult Cancer: A Report From the International Late Effects of Childhood Cancer Guideline Harmonization Group in Collaboration With the PanCareSurFup Consortium. J Clin Oncol. 2016;34(28):3440-50.

21. van der Kooi ALF, Mulder RL, Hudson MM, Kremer LCM, Skinner R, Constine LS, et al. Counseling and surveillance of obstetrical risks for female childhood, adolescent, and young adult cancer survivors: recommendations from the International Late Effects of Childhood Cancer Guideline Harmonization Group. Am J Obstet Gynecol. 2020.

For peer review only - http://bmjopen.bmj.com/site/about/guidelines.xhtml

For peer review only

22. Christen S, Roser K, Mulder RL, Ilic A, Lie HC, Loonen JJ, et al. Recommendations for the surveillance of cancer-related fatigue in childhood, adolescent, and young adult cancer survivors: a report from the International Late Effects of Childhood Cancer Guideline Harmonization Group. J Cancer Surviv. 2020.

23. Mulder RL, Hudson MM, Bhatia S, Landier W, Levitt G, Constine LS, et al. Updated Breast Cancer Surveillance Recommendations for Female Survivors of Childhood, Adolescent, and Young Adult Cancer From the International Guideline Harmonization Group. J Clin Oncol. 2020:JCO2000562.

24. Michel G, Mulder RL, van der Pal HJH, Skinner R, Bardi E, Brown MC, et al. Evidence-based recommendations for the organization of long-term follow-up care for childhood and adolescent cancer survivors: a report from the PanCareSurFup Guidelines Working Group. J Cancer Surviv. 2019;13(5):759-72.

25. Mulder RL, van der Pal HJH, Levitt GA, Skinner R, Kremer LCM, Brown MC, et al. Transition guidelines: An important step in the future care for childhood cancer survivors. A comprehensive definition as groundwork. Eur J Cancer. 2016;54:64-8.

26. Loonen JJ, Blijlevens NM, Prins J, Dona DJ, Den Hartogh J, Senden T, et al. Cancer Survivorship Care: Person Centered Care in a Multidisciplinary Shared Care Model. Int J Integr Care. 2018;18(1):4.

27. Dutch Childhood Oncology Group. Guidelines for follow-up in survivors of childhood cancer 5 years after diagnosis. 2010. Available at: https://www.skion.nl/workspace/uploads/richtlijn_follow-up_na_kinderkanker_deel_1_1.pdf. Accessed 17 Dec 2021.

28. van Kalsbeek RJ, van der Pal HJH, Hjorth L, Winther JF, Michel G, Haupt R, et al. The European multistakeholder PanCareFollowUp project: novel, person-centred survivorship care to improve care quality, effectiveness, cost-effectiveness and accessibility for cancer survivors and caregivers. Eur J Cancer. 2021;153:74-85.

29. van Kalsbeek RJ, Mulder RL, Haupt R, Muraca M, Hjorth L, Follin C, et al. The PanCareFollowUp Care Intervention: A European harmonised approach to person-centred guideline-based survivorship care after childhood, adolescent and young adult cancer. Eur J Cancer. 2022;162:34-44.

30. Haupt R, Essiaf S, Dellacasa C, Ronckers CM, Caruso S, Sugden E, et al. The 'Survivorship Passport' for childhood cancer survivors. Eur J Cancer. 2018;102:69-81.

31. Osborne RH, Elsworth GR, Whitfield K. The Health Education Impact Questionnaire (heiQ): an outcomes and evaluation measure for patient education and self-management interventions for people with chronic conditions. Patient Educ Couns. 2007;66(2):192-201.

32. Luttrell C, Quiroz S, Scrutton C, Bird K. Working Paper 308: Understanding and operationalising empowerment. 2009. Available at: https://cdn.odi.org/media/documents/5500.pdf. Accessed 17 Dec 2021.

For peer review only - http://bmjopen.bmj.com/site/about/guidelines.xhtml

33. European Patients' Froum. Background Brief: Patient Empowerment. 2015. Available at: https://www.eu-patient.eu/globalassets/campaign-patient-empowerment/epf_briefing_patientempowerment_2015.pdf. Accessed 17 Dec 2021.

34. Maunsell E, Lauzier S, Brunet J, Pelletier S, Osborne RH, Campbell HS. Health-related empowerment in cancer: validity of scales from the Health Education Impact Questionnaire. Cancer. 2014;120(20):3228-36.

35. Brunet J, Lauzier S, Campbell HS, Fillion L, Osborne RH, Maunsell E. Measurement invariance of English and French Health Education Impact Questionnaire (heiQ) empowerment scales validated for cancer. Qual Life Res. 2015;24(10):2375-84.

36. Osborne RH, Batterham R, Livingston J. The evaluation of chronic disease self-management support across settings: the international experience of the health education impact questionnaire quality monitoring system. Nurs Clin North Am. 2011;46(3):255-70.

37. Glasgow RE, Harden SM, Gaglio B, Rabin B, Smith ML, Porter GC, et al. RE-AIM Planning and Evaluation Framework: Adapting to New Science and Practice With a 20-Year Review. Front Public Health. 2019;7:64.

For peer review only

For peer review only - http://bmjopen.bmj.com/site/about/guidelines.xhtml

Figure legends

Figure 1. Overview of all patient-reported outcome measures (PROMs) and experience measures (PREMs), clinical outcomes, feasibility outcomes and health economic outcomes used in the Care Study. Outcomes that are specific for males or females are indicated as such between brackets. For the clinical outcomes, it is indicated whether they are assessed through a diagnostic test according to the guidelines (d), Survivor Questionnaire (q), or both (d+q). Other clinical outcomes are assessed through medical history and/or physical examination. Abbreviations: BPI = Brief Pain Inventory, BSI-18 = Brief Symptom Inventory-18, CD-RISC 25 = Connor-Davidson Resilience Scale (25 items), ET = Emotion Thermometer, HCP = health care provider, HEIQ = health education impact questionnaire, HRQoL = health-related quality of life, ICECAP-A = ICEpop CAPability measure for Adults, LH/FSH = luteinising hormone/follicle-stimulating hormone, PROMIS = Patient-Reported Outcomes Measurement Information System, PCL-5 = PTSD Checklist for DSM-5, QoL = quality of life, Satisfaction Qx = Satisfaction questionnaire by Blaauwbroek et al, SCP = Survivorship Care Plan, SDM-Q-9 = 9-item shared decision-making questionnaire (patient perspective), SF-36 = Short Form-36 (36 items, version 1), SQx = Survivor Questionnaire (part of the PanCareFollowUp Care Intervention), TSH = thyroid-stimulating hormone, SDM-Q-Doc = 9-item Shared Decision-Making Questionnaire (HCP perspective).

References: [a] Brunet J., Lauzier S., Campbell H.S., Fillion L., Osborne R.H., Maunsell E., Measurement invariance of English and French Health Education Impact Questionnaire (heiQ) empowerment scales validated for cancer. Qual Life Res, 2015. 24(10): p. 2375-84.; Osborne R.H., Elsworth G.R., Whitfield K., The Health Education Impact Questionnaire (heiQ): an outcomes and evaluation measure for patient education and self-management interventions for people with chronic conditions. Patient Educ Couns, 2007. 66(2): p. 192-201.

[b] Blaauwbroek, R., Tuinier, W., Jong, B. M., Kamps, W. A., & Postma, A. (2008). Shared care by paediatric oncologists and family doctors for long-term follow-up of adult childhood cancer survivors: a pilot study. Lancet Oncology, 9(3), 232-238. DOI: 10.1016/S1470-2045(08)70034-2.

[c] Kriston L., Scholl I., Holzel L., Simon D., Loh A., Harter M., The 9-item Shared Decision Making Questionnaire (SDM-Q-9). Development and psychometric properties in a primary care sample. Patient Educ Couns, 2010. 80(1): p. 94-9.; Rodenburg-Vandenbussche S., Pieterse A.H., Kroonenberg P.M., Scholl I., van der Weijden T., Luyten G.P., et al., Dutch Translation and Psychometric Testing of the 9-Item Shared Decision Making Questionnaire (SDM-Q-9) and Shared Decision Making Questionnaire-Physician Version (SDM-Q-Doc) in Primary and Secondary Care. PLoS One, 2015. 10(7): p. e0132158.

For peer review only - http://bmjopen.bmj.com/site/about/guidelines.xhtml

[d] Connor K.M., Davidson J.R., Development of a new resilience scale: the Connor-Davidson Resilience Scale (CD-RISC). Depress Anxiety, 2003. 18(2): p. 76-82.

[e] EQ-5D-5L: Herdman, M., et al., Development and preliminary testing of the new five-level version of EQ-5D (EQ-5D-5L). Qual Life Res, 2011. 20(10): p. 1727-36.; SF-36: Ware J.E., Jr., Gandek B., Overview of the SF-36 Health Survey and the International Quality of Life Assessment (IQOLA) Project. J Clin Epidemiol, 1998. 51(11): p. 903-12.; ICECAP-A: Al-Janabi, H., T.N. Flynn, and J. Coast, Development of a self-report measure of capability wellbeing for adults: the ICECAP-A. Qual Life Res, 2012. 21(1): p. 167-76.

[f] Derogatis, L.R., BSI 18 - Brief Symptom Inventory 18 - Administration, Scoring, and Procedures Manual. 2000: NCS Pearson Inc.

[g] Blevins, C.A., et al., The Posttraumatic Stress Disorder Checklist for DSM-5 (PCL-5): Development and Initial Psychometric Evaluation. J Trauma Stress, 2015. 28(6): p. 489-98.

[h] Mitchell, A.J., et al., Can the Distress Thermometer be improved by additional mood domains? Part I. Initial validation of the Emotion Thermometers tool. Psychooncology, 2010. 19(2): p. 125-33., Mitchell, A.J., et al., Can the Distress Thermometer be improved by additional mood domains? Part II. What is the optimal combination of Emotion Thermometers? Psychooncology, 2010. 19(2): p. 134-40.

[i] Christen, S. et al., Recommendations for the Surveillance of Cancer-Related Fatigue in Childhood, Adolescent and Young Adult Cancer Survivors: A Report from the International Late Effects of Childhood Cancer Guideline Harmonization Group and the PanCare Guidelines Group. Journal of Cancer Survivorship. 2020;14(6):923-938.

[j] Bingham Iii, C.O., et al., PROMIS Fatigue short forms are reliable and valid in adults with rheumatoid arthritis. J Patient Rep Outcomes, 2019. 3(1): p. 14.

[k] Cleeland, C.S. and K.M. Ryan, Pain assessment: global use of the Brief Pain Inventory. Ann Acad Med Singapore, 1994. 23(2): p. 129-38.

Figure 2. Flowchart of data collection after inclusion of an eligible survivor. Abbreviations: HCP = health care provider, PREMS = patient-reported experience measures, PROMS = patient-reported outcome measures, T1 = time point 1, T2 = time point 2, T3 = time point 3, T4 = time point 4, T5 = time point 5. The boxes describe for each time point the timing of data collection, the person providing data (survivor, HCP or both), the data collection instruments (Survivor Questionnaire, Treatment Summary or T1-T5 study questionnaire) and the types of outcomes collected. Depicted in blue is data collected for care, and in purple for research purposes.

For peer review only

For peer review only - http://bmjopen.bmj.com/site/about/guidelines.xhtml

**PROMs or PREMs: survivors**	Premature ovarian insufficiency (females) (d)	Neurocognitive problems: language	Telangiectasias of the eye
Empowerment (HEIQ)[a] (primary outcome)	Testosterone deficiency (males) (d)	Neurocognitive problems: memory	Xerophthalmia
Patient satisfaction (Satisfaction Qx)[b]	TSH deficiency (d)	Neurocognitive problems: motor integration	**Feasibility outcomes: survivor**
Shared decision-making (SDM-Q-9)[c]	*Gastro-intestinal*	Neurocognitive problems: processing speed	Received care according to SCP
Resilience (CD-RISC 25)[d]	Bowel obstruction	Psychological distress (q)	Success of communication
HRQoL (EQ-5D-5L, SF-36, ICECAP-A)[e]	Chronic enterocolitis	Stress-related mental disorder	Missing information
Psychological distress (BSI-18)[f]	Gastro-intestinal strictures or fistula	Suicidal ideation (q)	*Italian study site only:* Use of and satisfaction with SurPass
Post-traumatic stress symptoms (PCL-5)[g]	*Hepato-biliary*	Unemployment (q)	
Distress (ET)[h]	Cholelithiasis	*Renal and urinary tract*	**Feasibility outcomes: HCP (per clinic)**
Fatigue (SQx + PROMIS Fatigue – Short Form 8a +)[i,j]	Hepatobiliary dysfunction (d)	Bladder fibrosis	No. of eligible survivors invited
Pain (BPI)[k]	Hepatocellular liver injury (stage 1) (d)	Dysfunctional voiding (q)	No. of participating survivors per time point
Lifestyle (SQx)	Iron overload (d)	Glomerular kidney dysfunction (d)	No. of non-responders
Social functioning (SQx)	Liver cirrhosis	Haemorrhagic cystitis	Reasons for non-response
**Clinical outcomes**	Liver fibrosis	Hydronephrosis	No. of drop-outs per time point
Auditory	Liver synthetic dysfunction (d)	Tubular kidney dysfunction (d)	Reasons for drop-outs per time point
Hearing loss (d + q)	*Immunological*	Vesicoureteral reflux	Composition of multidisciplinary team
Tinnitus (q)	Spleen problems (overwhelming infections)	*Reproductive*	Use of the SCP
Cardiac	*Musculoskeletal*	Impaired fertility (q)	Reasons for non-use of SCP, if applicable
Arrhythmia (d + q)	Craniofacial growth problems	Impaired spermatogenesis (males) (d + q)	Shared decision making (HCP perspective; SDM-Q-Doc)[c]
Cardiomyopathy (d)	Osteonecrosis	Low birth weight of offspring (females) (q)	Extent to which SCP of participating survivors has been implemented and reasons for deviating
Pericardial disease (d)	Reduced bone mineral density (d)	Miscarriage (females) (q)	
Valvular heart disease (d)	Spine kyphosis	Physical sexual dysfunction (males) (q)	*Italian study site only:* no. of SurPasses delivered, recommendation brochures given and SurPasses shared with physicians, SurPass user statistics
Dental	Spine scoliosis	Premature birth of offspring (females) (q)	
Dental caries	*Neurological*	*Respiratory*	
Dental developmental problems	Cavernomas	Pulmonary dysfunction (d + q)	**Health economic outcomes: survivor**
Xerostomia (q)	Cerebrovascular accidents	*Subsequent neoplasm*	Time investment of survivor (preparation for clinic visit, travel, total time in clinic, follow-up appointments)
Dermatologic	Neurogenic bladder	Subsequent neoplasm (benign or malignant) (d + q)	
Alopecia	Neurogenic bowel	*Vascular*	Time investment of relatives (travel, total time in clinic, follow-up appointments)
Endocrine	Optic chiasm neuropathy	Aneurysms	
ACTH deficiency (d)	Pain (q)	Asymptomatic coronary artery disease	Travel costs of survivor and relatives
Amenorrhea (females) (q)	Peripheral motor neuropathy (q)	Carotid artery disease	Other extra costs for survivor and relatives
Central precocious puberty (d)	Peripheral sensory neuropathy (q)	Dyslipidaemia (d)	Loss of time for survivor and relatives at paid work or in education
Diabetes mellitus (d)	*Psychosocial and neurocognitive*	Hypertension	
Failure in pubertal progression	Adjustment difficulties	*Visual*	**Health economic outcomes: HCP**
Growth hormone deficiency (d)	Anxiety (q)	Cataract	Time investment of HCP and other staff tasks related to clinic visit (preparation, clinic visit, tasks following clinic visit, follow-up call)
Hyperthyroidism (d)	Behavioural problems	Chronic painful eye	
Hypothyroidism (peripheral) (d)	Fatigue (q)	Glaucoma	
Impaired glucose metabolism (d)	Low educational status (q)	Keratitis	Costs for diagnostic and screening tests
LH/FSH deficiency (d)	Neurocognitive problems: academics	Lacrimal duct atrophy	Costs for other consumables for clinic visit
Obesity	Neurocognitive problems: attention	Maculopathy	
Overweight	Neurocognitive problems: executive function	Papillopathy	
Premature menopause (females) (d)	Neurocognitive problems: intelligence	Retinopathy	

For peer review only

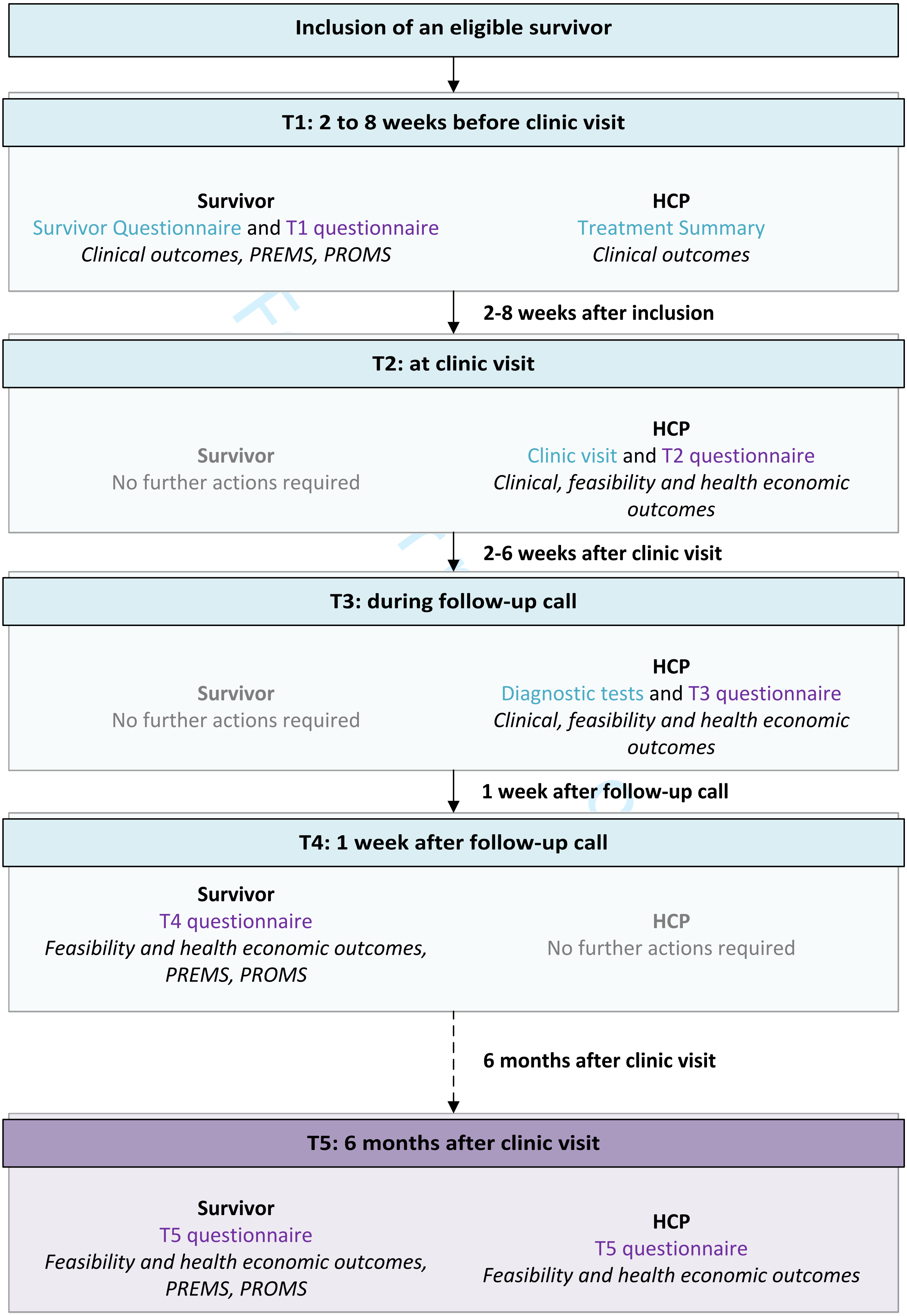
Inclusion of an eligible survivor
T1: 2 to 8 weeks before clinic visit
Survivor
Survivor Questionnaire and T1 questionnaire
Clinical outcomes, PREMS, PROMS
HCP
Treatment Summary
Clinical outcomes
2-8 weeks after inclusion
T2: at clinic visit
Survivor
No further actions required
HCP
Clinic visit and T2 questionnaire
Clinical, feasibility and health economic outcomes
2-6 weeks after clinic visit
T3: during follow-up call
Survivor
No further actions required
HCP
Diagnostic tests and T3 questionnaire
Clinical, feasibility and health economic outcomes
1 week after follow-up call
T4: 1 week after follow-up call
Survivor
T4 questionnaire
Feasibility and health economic outcomes, PREMS, PROMS
HCP
No further actions required
6 months after clinic visit
T5: 6 months after clinic visit
Survivor
T5 questionnaire
Feasibility and health economic outcomes, PREMS, PROMS
HCP
T5 questionnaire
Feasibility and health economic outcomes

Appendix A: Recruitment strategy of each study site

Sweden starts with inviting a random sample, prioritising survivors who are lost to follow-up or have not visited the study site in the past five years, and might invite survivors who received care more recently depending on the recruitment rate among the initial population.

Italy starts with inviting survivors who already have a scheduled appointment at their clinic, but who had not already received the Survivorship Passport, and are resident in the Liguria region. They will invite 350 to 400 survivors to be able to include 200 survivors. They will subsequently recruit scheduled survivors resident in other regions, and if the number is still insufficient, they will actively invite other survivors to the clinic.

The Czech Republic starts with selecting a random sample of 250 survivors from the clinic's database whom they will gradually invite over the recruitment period. If more survivors need to be invited to reach the inclusion aim within the recruitment period, they will invite survivors who have a scheduled appointment at their clinic and who meet the study inclusion criteria

Belgium starts to invite, in alphabetical order the survivors of 18 year and older with a primary cancer diagnosis with a date of diagnosis in or before 1990, regardless of whether or not they already received some long-term follow-up. Simultaneously, 20 survivors who were scheduled for a clinic visit in March and April 2021 have also been invited to participate in this study. In the second wave, they will invite the survivors with a diagnosis in 1990-2000 in alphabetic order. And, if needed, the survivors diagnosed in 2001-2020, again in alphabetic order.

For peer review only

SPIRIT

STANDARD PROTOCOL ITEMS: RECOMMENDATIONS FOR INTERVENTIONAL TRIALS

SPIRIT 2013 Checklist: Recommended items to address in a clinical trial protocol and related documents*

Section/item	Item No	Description	Addressed on page number
**Administrative information**			
Title	1	Descriptive title identifying the study design, population, interventions, and, if applicable, trial acronym	1
Trial registration	2a	Trial identifier and registry name. If not yet registered, name of intended registry	6
	2b	All items from the World Health Organization Trial Registration Data Set	1-25
Protocol version	3	Date and version identifier	8, 17, 18
Funding	4	Sources and types of financial, material, and other support	18
Roles and responsibilities	5a	Names, affiliations, and roles of protocol contributors	1,2
	5b	Name and contact information for the trial sponsor	18
	5c	Role of study sponsor and funders, if any, in study design; collection, management, analysis, and interpretation of data; writing of the report; and the decision to submit the report for publication, including whether they will have ultimate authority over any of these activities	18
	5d	Composition, roles, and responsibilities of the coordinating centre, steering committee, endpoint adjudication committee, data management team, and other individuals or groups overseeing the trial, if applicable (see Item 21a for data monitoring committee)	18, 19

For peer review only

For peer review only - http://bmjopen.bmj.com/site/about/guidelines.xhtml

**Introduction**			
Background and rationale	6a	Description of research question and justification for undertaking the trial, including summary of relevant studies (published and unpublished) examining benefits and harms for each intervention	7, 8
	6b	Explanation for choice of comparators	8, 11, 12
Objectives	7	Specific objectives or hypotheses	8
Trial design	8	Description of trial design including type of trial (eg, parallel group, crossover, factorial, single group), allocation ratio, and framework (eg, superiority, equivalence, noninferiority, exploratory)	9
**Methods: Participants, interventions, and outcomes**			
Study setting	9	Description of study settings (eg, community clinic, academic hospital) and list of countries where data will be collected. Reference to where list of study sites can be obtained	9
Eligibility criteria	10	Inclusion and exclusion criteria for participants. If applicable, eligibility criteria for study centres and individuals who will perform the interventions (eg, surgeons, psychotherapists)	9
Interventions	11a	Interventions for each group with sufficient detail to allow replication, including how and when they will be administered	8, 9, 10, ref 29
	11b	Criteria for discontinuing or modifying allocated interventions for a given trial participant (eg, drug dose change in response to harms, participant request, or improving/worsening disease)	10
	11c	Strategies to improve adherence to intervention protocols, and any procedures for monitoring adherence (eg, drug tablet return, laboratory tests)	N/A
	11d	Relevant concomitant care and interventions that are permitted or prohibited during the trial	N/A
Outcomes	12	Primary, secondary, and other outcomes, including the specific measurement variable (eg, systolic blood pressure), analysis metric (eg, change from baseline, final value, time to event), method of aggregation (eg, median, proportion), and time point for each outcome. Explanation of the clinical relevance of chosen efficacy and harm outcomes is strongly recommended	11, 12
Participant timeline	13	Time schedule of enrolment, interventions (including any run-ins and washouts), assessments, and visits for participants. A schematic diagram is highly recommended (see Figure)	10, Fig 2

For peer review only - http://bmjopen.bmj.com/site/about/guidelines.xhtml

Sample size	14	Estimated number of participants needed to achieve study objectives and how it was determined, including clinical and statistical assumptions supporting any sample size calculations	9, 10, 13, 14
Recruitment	15	Strategies for achieving adequate participant enrolment to reach target sample size	10, 15, App A
**Methods: Assignment of interventions (for controlled trials)**			
Allocation:			
Sequence generation	16a	Method of generating the allocation sequence (eg, computer-generated random numbers), and list of any factors for stratification. To reduce predictability of a random sequence, details of any planned restriction (eg, blocking) should be provided in a separate document that is unavailable to those who enrol participants or assign interventions	N/A
Allocation concealment mechanism	16b	Mechanism of implementing the allocation sequence (eg, central telephone; sequentially numbered, opaque, sealed envelopes), describing any steps to conceal the sequence until interventions are assigned	N/A
Implementation	16c	Who will generate the allocation sequence, who will enrol participants, and who will assign participants to interventions	N/A
Blinding (masking)	17a	Who will be blinded after assignment to interventions (eg, trial participants, care providers, outcome assessors, data analysts), and how	N/A
	17b	If blinded, circumstances under which unblinding is permissible, and procedure for revealing a participant's allocated intervention during the trial	N/A
**Methods: Data collection, management, and analysis**			
Data collection methods	18a	Plans for assessment and collection of outcome, baseline, and other trial data, including any related processes to promote data quality (eg, duplicate measurements, training of assessors) and a description of study instruments (eg, questionnaires, laboratory tests) along with their reliability and validity, if known. Reference to where data collection forms can be found, if not in the protocol	14
	18b	Plans to promote participant retention and complete follow-up, including list of any outcome data to be collected for participants who discontinue or deviate from intervention protocols	10, 15, App A

For peer review only - http://bmjopen.bmj.com/site/about/guidelines.xhtml

Data management	19	Plans for data entry, coding, security, and storage, including any related processes to promote data quality (eg, double data entry; range checks for data values). Reference to where details of data management procedures can be found, if not in the protocol	15, 16
Statistical methods	20a	Statistical methods for analysing primary and secondary outcomes. Reference to where other details of the statistical analysis plan can be found, if not in the protocol	14, 15
	20b	Methods for any additional analyses (eg, subgroup and adjusted analyses)	14
	20c	Definition of analysis population relating to protocol non-adherence (eg, as randomised analysis), and any statistical methods to handle missing data (eg, multiple imputation)	14
**Methods: Monitoring**			
Data monitoring	21a	Composition of data monitoring committee (DMC); summary of its role and reporting structure; statement of whether it is independent from the sponsor and competing interests; and reference to where further details about its charter can be found, if not in the protocol. Alternatively, an explanation of why a DMC is not needed	19
	21b	Description of any interim analyses and stopping guidelines, including who will have access to these interim results and make the final decision to terminate the trial	N/A
Harms	22	Plans for collecting, assessing, reporting, and managing solicited and spontaneously reported adverse events and other unintended effects of trial interventions or trial conduct	17
Auditing	23	Frequency and procedures for auditing trial conduct, if any, and whether the process will be independent from investigators and the sponsor	19
**Ethics and dissemination**			
Research ethics approval	24	Plans for seeking research ethics committee/institutional review board (REC/IRB) approval	16
Protocol amendments	25	Plans for communicating important protocol modifications (eg, changes to eligibility criteria, outcomes, analyses) to relevant parties (eg, investigators, REC/IRBs, trial participants, trial registries, journals, regulators)	18

For peer review only - http://bmjopen.bmj.com/site/about/guidelines.xhtml

Consent or assent	26a	Who will obtain informed consent or assent from potential trial participants or authorised surrogates, and how (see Item 32)	10, 16
	26b	Additional consent provisions for collection and use of participant data and biological specimens in ancillary studies, if applicable	N/A
Confidentiality	27	How personal information about potential and enrolled participants will be collected, shared, and maintained in order to protect confidentiality before, during, and after the trial	15, 16, 19
Declaration of interests	28	Financial and other competing interests for principal investigators for the overall trial and each study site	18
Access to data	29	Statement of who will have access to the final trial dataset, and disclosure of contractual agreements that limit such access for investigators	19
Ancillary and post-trial care	30	Provisions, if any, for ancillary and post-trial care, and for compensation to those who suffer harm from trial participation	17
Dissemination policy	31a	Plans for investigators and sponsor to communicate trial results to participants, healthcare professionals, the public, and other relevant groups (eg, via publication, reporting in results databases, or other data sharing arrangements), including any publication restrictions	17
	31b	Authorship eligibility guidelines and any intended use of professional writers	19
	31c	Plans, if any, for granting public access to the full protocol, participant-level dataset, and statistical code	19
**Appendices**			
Informed consent materials	32	Model consent form and other related documentation given to participants and authorised surrogates	Submitted separately
Biological specimens	33	Plans for collection, laboratory evaluation, and storage of biological specimens for genetic or molecular analysis in the current trial and for future use in ancillary studies, if applicable	N/A

*It is strongly recommended that this checklist be read in conjunction with the SPIRIT 2013 Explanation & Elaboration for important clarification on the items. Amendments to the protocol should be tracked and dated. The SPIRIT checklist is copyrighted by the SPIRIT Group under the Creative Commons "Attribution-NonCommercial-NoDerivs 3.0 Unported" license.

For peer review only - http://bmjopen.bmj.com/site/about/guidelines.xhtml

BMJ Open

Evaluating the feasibility, effectiveness and costs of implementing person-centred follow-up care for childhood cancer survivors in four European countries: the PanCareFollowUp Care prospective cohort study protocol

Journal:	*BMJ Open*
Manuscript ID	bmjopen-2022-063134.R2
Article Type:	Protocol
Date Submitted by the Author:	28-Oct-2022
Complete List of Authors:	van Kalsbeek, Rebecca; Princess Maxima Center for Pediatric Oncology<br>Korevaar, J; Netherlands Institute for Health Services Research (NIVEL)<br>Rijken, Mieke; Netherlands Institute for Health Services Research (NIVEL); University of Eastern Finland - Kuopio Campus, Department of Health and Social Care Management<br>Haupt, Riccardo; IRCCS Istituto Giannina Gaslini, Epidemiology and Biostatistics Unit and DOPO clinic<br>Muraca, Monica; IRCCS Istituto Giannina Gaslini, Epidemiology and Biostatistics Unit and DOPO clinic<br>Kepák, Tomáš; Masaryk University Faculty of Medicine, International Clinical Research Centre (FNUSA-ICRC) at St. Anne's University Hospital<br>Kepakova, Katerina; Masaryk University Faculty of Medicine, International Clinical Research Centre (FNUSA-ICRC) at St. Anne's University Hospital<br>Blondeel, Anne; European Society for Pediatric Oncology (SIOP Europe)<br>Boes, Stefan; University of Lucerne, Department of Health Sciences and Medicine<br>Frederiksen, Line Elmerdahl; Danish Cancer Society Research Center, Childhood Cancer Research Group<br>Essiaf, Samira; European Society for Pediatric Oncology (SIOP Europe)<br>Winther, Jeanette; Danish Cancer Society Research Center, Childhood Cancer Research Group; Aarhus Universitet, Department of Clinical Medicine and Faculty of Health<br>Hermens, Rosella; Radboudumc, Scientific Institute for Quality of Healthcare (IQ Healthcare)<br>Kienesberger, Anita; Childhood Cancer International Europe<br>Loonen, Jacqueline; Radboudumc, Department of Hematology<br>Michel, Gisela; University of Lucerne, Department of Health Sciences and Medicine<br>Mulder, Renée; Princess Maxima Center for Pediatric Oncology<br>O'Brien, Kylie; Pintail, Limited<br>van der Pal, Helena; Princess Maxima Center for Pediatric Oncology; PanCare<br>Pluijm, Saskia ; Princess Maxima Center for Pediatric Oncology<br>Roser, Katharina; University of Lucerne, Department of Health Sciences and Medicine<br>Skinner, Roderick; Newcastle University Centre for Cancer, Wolfson Childhood Cancer Research Centre; Great North Children's Hospital,

BMJ Open

	Royal Victoria Infirmary<br>Renard, Marleen; KU Leuven University Hospitals Leuven, Department of Paediatric Haematology and Oncology<br>Uyttebroeck, Anne; KU Leuven University Hospitals Leuven, Department of Paediatric Haematology and Oncology<br>Follin, Cecilia; Skane University Hospital, Department of Clinical Sciences Lund, Oncology<br>Hjorth, Lars; Skane University Hospital, Department of Clinical Sciences Lund, Paediatrics<br>Kremer, Leontien; Princess Maxima Center for Pediatric Oncology; Emma Childrens' Hospital UMC, Department of Paediatrics
**Primary Subject Heading**:	Oncology
Secondary Subject Heading:	Health policy, Oncology, Paediatrics, Patient-centred medicine, Research methods
Keywords:	International health services < HEALTH SERVICES ADMINISTRATION & MANAGEMENT, Health economics < HEALTH SERVICES ADMINISTRATION & MANAGEMENT, Paediatric oncology < ONCOLOGY, Paediatric oncology < PAEDIATRICS, Organisation of health services < HEALTH SERVICES ADMINISTRATION & MANAGEMENT

Evaluating the feasibility, effectiveness and costs of implementing person-centred follow-up care for childhood cancer survivors in four European countries: the PanCareFollowUp Care prospective cohort study protocol

Rebecca J. van Kalsbeek[a], Joke C. Korevaar[b], Mieke Rijken[b,c], Riccardo Haupt[d], Monica Muraca[d], Tomas Kepak[e], Katerina Kepakova[e], Anne Blondeel[f], Stefan Boes[g], Line Elmerdahl Frederiksen[h], Samira Essiaf[f], Jeanette Falck Winther[h,i], Rosella P.M.G. Hermens[j], Anita Kienesberger[k], Jacqueline J. Loonen[l], Gisela Michel[g], Renée L. Mulder[a], Kylie O'Brien[m], Helena J.H. van der Pal[a,n], Saskia M.F. Pluijm[a], Katharina Roser[g], Roderick Skinner[o,p,q], Marleen Renard[r], Anne Uyttebroeck[r], Cecilia Follin[s], Lars Hjorth[t], Leontien C. M. Kremer[a,u,v]

Affiliations:

[a] Princess Máxima Centre for Paediatric Oncology, Heidelberglaan 25, 3584 CS, Utrecht, the Netherlands

[b] Netherlands Institute for Health Services Research (Nivel), P.O. Box 1568, 3500 BN Utrecht, the Netherlands

[c] University of Eastern Finland, Department of Health and Social Management, P.O. Box 1627, FI-70211 Kuopio, Finland

[d] Epidemiology and Biostatistics Unit and DOPO clinic, IRCCS Istituto Giannina Gaslini, Via G. Gaslini, 5, 16147 Genoa, Italy

[e] International Clinical Research Centre (FNUSA-ICRC) at St. Anne's University Hospital, Masaryk University, Pekařská 53, Brno 656 91, Czech Republic

[f] European Society for Paediatric Oncology, c/o BLSI, Clos Chapelle-aux-Champs 30, Bte 1.30.30, BE-1200 Brussels, Belgium

[g] University of Lucerne, Department of Health Sciences and Medicine, Frohburgstrasse 3, P.O. Box 4466, 6002 Lucerne, Switzerland

[h] Childhood Cancer Research Group, Danish Cancer Society Research Centre, Strandboulevarden 49, 2100 Copenhagen, Denmark

[i] Department of Clinical Medicine, Faculty of Health, Aarhus University and Aarhus University Hospital, Palle Juul-Jensens Boulevard 82, 8200 Aarhus, Denmark

For peer review only

For peer review only - http://bmjopen.bmj.com/site/about/guidelines.xhtml

[j] Radboud University Medical Centre, Radboud Institute for Health Sciences, Scientific Institute for Quality of Healthcare (IQ Healthcare), Geert Grooteplein 21, 6525 EZ, Nijmegen, the Netherlands

[k] Childhood Cancer International Europe, Servitengasse 5/16, 1090 Vienna, Austria

[l] Radboud University Medical Centre, Radboud Institute for Health Sciences, Department of Hematology, Geert Grooteplein Zuid 10, 6525 GA, Nijmegen, the Netherlands

[m] Pintail, Limited, 77 Springhill Avenue, Blackrock, Dublin, Ireland

[n] PanCare, Jacobus Bellamylaan 16, 1401 AZ Bussum, the Netherlands

[o] Newcastle University Centre for Cancer, Wolfson Childhood Cancer Research Centre, Herschel Building, Brewery Lane, Newcastle upon Tyne, NE1 7RU, United Kingdom

[p] Great North Children's Hospital, Royal Victoria Infirmary, Queen Victoria Road, Newcastle upon Tyne, NE1 4 LP, United Kingdom

[q] Translational and Clinical Research Institute, Wolfson Childhood Cancer Research Centre, Herschel Building, Brewery Lane, Newcastle upon Tyne, NE1 7RU, United Kingdom

[r] Department of Oncology, Paediatric Oncology, KU Leuven, Department of Paediatric Haematology and Oncology, University Hospitals Leuven, Herestraat 49, 3000 Leuven, Belgium

[s] Lund University, Skane University Hospital, Department of Clinical Sciences Lund, Oncology, Lasarettsgatan 40, 221 85 Lund, Sweden

[t] Lund University, Skane University Hospital, Department of Clinical Sciences Lund, Paediatrics, Lasarettsgatan 40, 221 85 Lund, Sweden

[u] Department of Paediatrics, Emma Children's Hospital, Amsterdam UMC, Meibergdreef 9, 1105 AZ Amsterdam, the Netherlands

[v] Faculty of Medicine, Utrecht University and Utrecht Medical Centre, Universiteitsweg 98, 3584 CG Utrecht, the Netherlands

Corresponding author: Rebecca J. van Kalsbeek
R.J.vanKalsbeek@prinsesmaximacentrum.nl
Princess Máxima Center for Pediatric Oncology
Heidelberglaan 25
3584 CS Utrecht, The Netherlands
+ 31 88 972 72 72

For peer review only - http://bmjopen.bmj.com/site/about/guidelines.xhtml

**Public title:**	Impact of person-centred follow-up care for European childhood cancer survivors: PanCareFollowUp Care

Keywords: Childhood cancer; survivorship; person-centered care; shared decision-making; empowerment; quality of life; clinical trial protocol; cohort study; prospective study; multicenter study, cost evaluation

**Word count:**	4137 words (including Box 1)
**Abstract word count:**	297 words
**Tables:**	1
**Figures:**	3
**Appendices:**	1

For peer review only - http://bmjopen.bmj.com/site/about/guidelines.xhtml

List of abbreviations:

FAIR = Findable, accessible, interoperable and reusable

GCP = Good Clinical Practice

HCP(s) = Health care provider(s)IGHG = International Late Effects of Childhood Cancer Guideline Harmonization Group

PanCare = Pan-European Network for Care of Survivors after Childhood and Adolescent Cancer

PROM = Patient-reported outcome measure

PREM = Patient-reported experience measure

RE-AIM = Reach, Effectiveness, Adoption, Implementation and Maintenance

SD = Standard deviation

SurPass = Survivorship Passport

T1-5 = Time points 1-5

For peer review only

For peer review only - http://bmjopen.bmj.com/site/about/guidelines.xhtml

Abstract

Introduction – Long-term survival after childhood cancer often comes at the expense of late, adverse health conditions. However, survivorship care is often not available for adult survivors in Europe. The PanCareFollowUp Consortium therefore developed the PanCareFollowUp Care Intervention, an innovative person-centred survivorship care model based on experiences in the Netherlands. This paper describes the protocol of the prospective cohort study (Care Study) to evaluate the feasibility and the health economic, clinical and patient-reported outcomes of implementing PanCareFollowUp Care as usual care in four European countries.

Methods and analysis – In this prospective, longitudinal cohort study with at least six months of follow-up, 800 childhood cancer survivors will receive the PanCareFollowUp Care Intervention across four study sites in Belgium, Czech Republic, Italy and Sweden, representing different health care systems. The PanCareFollowUp Care Intervention will be evaluated according to the RE-AIM (Reach, Effectiveness, Adoption, Implementation and Maintenance) framework. Clinical and research data are collected through questionnaires, a clinic visit for multiple medical assessments and a follow-up call. The primary outcome is empowerment, assessed with the Health Education Impact Questionnaire (HEIQ). A central data centre will perform quality checks, data cleaning, data validation, and provide support in data analysis. Multilevel models will be used for repeated outcome measures, with subgroup analysis, e.g. by centre, attained age, sex or diagnosis.

Ethics and dissemination - This study will be conducted in accordance with the guidelines of Good Clinical Practice and the Declaration of Helsinki. The study protocol has been reviewed and approved by all relevant ethics committees. The evidence and insights gained by this study will be summarised in a Replication Manual, also including the tools required to implement the PanCareFollowUp Care Intervention in other countries. This Replication Manual will become freely available through PanCare and will be disseminated through policy and press releases.

Trial registration - NL8918, registered at the Netherlands Trial Register at 24 September 2020, https://www.trialregister.nl/trial/8918.

For peer review only - http://bmjopen.bmj.com/site/about/guidelines.xhtml

Article summary

Strengths and limitations of this study

- The PanCareFollowUp Care Study is designed and conducted together with survivor representatives, ensuring the outcome measures are relevant for survivors and that PanCareFollowUp Care meets their needs and expectations.
- We include survivors from four different European countries, representing a variety of health care systems across Europe; and their experiences are used to improve the PanCareFollowUp Care Intervention before free distribution of the materials in a Replication Manual.
- The PanCareFollowUp Care Intervention is evaluated in a real life setting with a minimal number of exclusion criteria.
- Since the Care Study has a limited follow-up time, a model-based economic evaluation will complement the analyses.
- Participants are their own controls and effects are evaluated as changes from baseline within an individual or institution.

For peer review only

For peer review only - http://bmjopen.bmj.com/site/about/guidelines.xhtml

Introduction

Over the last decades, five-year survival rates of childhood cancer in Europe have increased substantially, from 30% in the 1970s to 80% in the early 2000s (1). Today, the European population of childhood cancer survivors, estimated at minimally 300,000, is rising by about 12,000 per year (2). Yet, many survivors not only experience the burden of previous cancer diagnosis, but also face treatment-related late effects (3, 4). These may become apparent years or even decades after finishing therapy (5) and might have a significant adverse impact on quality of life (6, 7). Moreover, the transition from paediatric to adult health care settings often lacks continuity. As a result, many adults who survived childhood cancer have increased health care use and experience problems in participation, which generate a substantial burden for survivors and societies in general (8-10). Early detection of new health conditions is essential as it could prevent further harm (11). This requires lifelong survivorship care with frequent adaptations of the follow-up plan.

Currently, only one third of European paediatric oncology clinics provide survivorship care to adult survivors of childhood cancer (12). In 2006, an international group of paediatric oncologists, psychologists, nurses, epidemiologists, survivors and their parents agreed in the Erice statement that has recently been updated and reconfirmed (13, 14) that follow-up care should be available and accessible for all survivors throughout their lifespan.

In the past decade, international evidence-based clinical practice guidelines have been developed to support early detection and treatment of (a)symptomatic late effects, including those developed by the International Late Effects of Childhood Cancer Guideline Harmonization Group (IGHG), sometimes in collaboration with the PanCareSurFup project (15-23). A European model of care guideline is published and guidelines for the transition from childhood to adult health care settings and health promotion are currently being developed (24, 25). Yet, implementation lags behind. Recently, a person-centred approach for survivorship care for adult survivors has been implemented in Nijmegen, the Netherlands (26). All Dutch survivors of childhood cancer are invited for follow-up care by a long-term follow-up care clinic, in which multidisciplinary teams deliver person-centred care based on contemporary surveillance guidelines (27). The first positive effects of this person-centred approach have been reported (24, 26). The next step is to validate this person-centred approach for survivorship care in other countries.

The PanCareFollowUp Consortium, established in 2018, is a unique multidisciplinary European collaboration between 14 project partners from 10 European countries, including survivors (www.pancarefollowup.eu) (28). The aim of the consortium is to improve the quality of life for

For peer review only - http://bmjopen.bmj.com/site/about/guidelines.xhtml

survivors of childhood, adolescent and young adult cancer by bringing evidence-based, person-centred care to clinical practice. The PanCareFollowUp Consortium has developed two interventions: 1) a person-centred and guideline-based model of survivorship care (PanCareFollowUp Care Intervention) (see Box 1) (29) and 2) an eHealth lifestyle coaching model (PanCareFollowUp Lifestyle Intervention). The protocol of the first intervention is described in this paper (version 3, January 21st, 2021), the protocol of the second one will be described separately. Both will be evaluated within the PanCareFollowUp project. The consortium published a Care Intervention Manual that contains instructions and tools required for implementing the PanCareFollowUp Care Intervention. At the project end, Replication Manuals that contain the instructions and tools required for implementation of the PanCareFollowUp Interventions will be freely distributed.

The overall aim of the PanCareFollowUp Care Study is to evaluate the feasibility, effectiveness and costs of implementing PanCareFollowUp Care as usual care for adult survivors of childhood cancer in four study sites in four European countries. Four objectives have been formulated: 1) To what extent is implementing PanCareFollowUp Care in the participating study sites feasible?; 2) What are the patient-reported experiences and outcomes, including survivor empowerment, of PanCareFollowUp Care and how do they change?; 3) What is the number and nature of pre-existing and new clinical events detected by PanCareFollowUp Care among participating survivors?; and 4) What are the short-term (six months) and projected long-term costs per unit change of empowerment and other outcomes after implementing PanCareFollowUp Care from the perspective of survivors and health care providers (HCPs)?

Box 1: The PanCareFollowUp Care Intervention

The PanCareFollowUp Care Intervention is based on a person-centred care model (26) that aims to meet the physical, psychological and social needs of (adult) survivors of childhood cancer through shared decision-making about prevention, surveillance and treatment options. The Care Intervention consists of three steps:

a) *Preparation of the clinic visit by both the survivor and the health care provider (HCP).* The survivor provides information about their health, wellbeing, needs and preferences by completing the PanCareFollowUp Survivor Questionnaire. The HCP prepares a Treatment Summary describing the childhood cancer treatment that the survivor has received, reviews the relevant surveillance recommendations and the PanCareFollowUp Survivor

For peer review only - http://bmjopen.bmj.com/site/about/guidelines.xhtml

Questionnaire provided by the survivor, and thereupon prepares the Standard Survivorship Care Plan.

b) *Clinic visit including tailored follow-up care.* After obtaining a medical history and performing a physical examination, the survivor and HCP jointly discuss the results of the Survivor Questionnaire, and the Standard Survivorship Care Plan. Together, they agree on a plan for diagnostic tests and potential referral if needed, based on surveillance guidelines or clinical indication. Based on these shared decisions, as well as potential test results, the HCP creates a Draft Individualised Survivorship Care Plan and provides tailored health education.

c) *Follow-up call.* The survivor and HCP discuss the test results and the preferred model of care for future follow-up care. The results of these shared decisions are incorporated in the final Individualised Survivorship Care Plan, that the survivor may share with other HCPs.

The PanCareFollowUp Care Intervention ends after co-creation and delivery of the Individualised Survivorship Care Plan. Survivors will thereafter remain under surveillance either at or under the guidance of their clinic, frequently adjusting their Individualised Survivorship Care Plan when needed.

Methods and analysis

Study population, setting and recruitment

Survivors fulfil the inclusion criteria if they are or have been: diagnosed with cancer before the age of 19 years; treated or registered at one of the four study sites; treated with chemotherapy and/or radiation therapy for childhood cancer with or without surgery; at least five years from primary cancer diagnosis; at least one year off treatment (also applying to treatment of subsequent benign or malignant neoplasms or relapse of the primary cancer); and currently at least 16 years of age.

Exclusion criteria consist of: being unable to complete the study questionnaires because of severe neurocognitive sequelae or insufficient understanding of the language used (even with help from another person); or having previously received complete follow-up care that is similar to the care as described in the PanCareFollowUp Care Intervention Manual (Box 1).

This international prospective cohort study will be conducted at four study sites located in four European countries: Belgium, Czech Republic, Italy, and Sweden. All sites currently provide long-term follow-up care, either within a paediatric (Belgium, Italy) or adult (Czech Republic, Sweden) oncology centre, using a set of (inter)national guidelines and protocols. Each study site aims to

For peer review only - http://bmjopen.bmj.com/site/about/guidelines.xhtml

include 200 survivors who complete the study. With an estimated non-response and early drop-out (informed consent signed, but no actual participation in the study) of 40 to 50% based on previous experience and an estimated late drop-out (at any point after completing the T1 questionnaire) of 5-10% during the study, approximately 350 to 400 survivors will therefore be invited at each site. To assess the feasibility of this recruitment strategy, each centre screened their respective registries and estimated a total of 5,944 eligible survivors.

Each study site developed a recruitment strategy within the prerequisites of this study, that fits best within their own logistics (Appendix A). Selected survivors will be invited by an invitation letter, an invitation e-mail or by phone (depending on the usual procedure at each study site), and receive an information sheet, including contact details for additional information, and an informed consent form. Reasons for non-participation can be provided. One option of the pre-set reasons is 'not participating because the questionnaires are being provided via internet'. In this case, the study site may decide to offer the option for paper questionnaires. Survivors who give informed consent but do not respond to the first questionnaire, even after reminders, are considered early drop-outs and will be excluded from the study, as essential data about these survivors will not be available. The first participant was enrolled in February 2021, and at 1 March 2022 456 participants were enrolled and completed the clinic visit. The estimated last inclusion is on 30 September 2022, with last data collection 31 May 2023.

Participating survivors can withdraw from the study at any time if they wish. They are not obliged to provide a reason for withdrawal, although it will be asked and recorded if available. To assess representativeness of the final study sample, the four centres will provide aggregated data about their total eligible population of survivors including population distributions of gender, current age, age at diagnosis, type of cancer and distance to the late effects clinic. This will be compared to the distributions among the included survivors per clinic.

During recruitment and data collection, careful monitoring of enrolment, (non-)response, reasons for non-response and early and late drop-out will be performed by the four study sites in close collaboration with the central data centre at the Danish Cancer Society Research Centre.

Intervention

Survivors of childhood cancer who receive PanCareFollowUp Care (i.e., care in accordance with the PanCareFollowUp Care Intervention Manual and as outlined in Box 1) will be followed up until six months after the clinic visit. The implementation of person-centred care in this project is facilitated by a narrated Powerpoint and an on-site workshop for all HCPs involved in the study. An add-on

For peer review only - http://bmjopen.bmj.com/site/about/guidelines.xhtml

study investigating the feasibility of delivering PanCareFollowUp Care using the digital Survivorship Passport (SurPass) tool (30) will be conducted at the Italian clinic, where SurPass is already implemented.

Primary and secondary outcomes

This study uses a variety of outcomes to answer the four research objectives (Figure 1). These are measured from time point 1 (T1) before the clinic visit until T5 at six months after the clinic visit (Figure 2). Outcomes are provided by survivors and HCPs through questionnaires, a clinic visit and diagnostic tests.

1) To what extent is implementing PanCareFollowUp Care in the participating study sites feasible?

Feasibility of implementation is of major importance to ensure sustainability of the PanCareFollowUp Care Intervention. Therefore, feasibility indicators measured by questionnaires among survivors and HCPs as well as an evaluation of barriers and facilitators are included to inform about the experiences of implementing PanCareFollowUp Care (Figure 2). Items include, among others, drop-outs at different time-points, use of and experiences with the Survivorship Care Plan, and shared-decision making (Figure 1).

2) What are the experiences and outcomes as reported by participating survivors receiving PanCareFollowUp Care?

The primary outcome for this study is empowerment measured by the Health Education Impact Questionnaire (HEIQ) (31). Empowerment has been defined by the EU Joint Action on Patient Safety and Quality of Care as a 'multidimensional process that helps people gain control over their own lives and increase their capacity to act on issues that they themselves define as important', a definition adapted from Lutrell et al. (32, 33). Empowerment has been selected as the primary outcome because childhood cancer survivors encounter several transition moments starting from diagnosis, after which a greater responsibility for their own health and care is required. It is essential that survivors receive the support they need to manage and advocate for their needs. Moreover, empowerment is important to manage future health problems. We have included six of the eight scales of the HEIQ relevant to cancer survivors in our study (Social integration and support, Health service navigation, Constructive attitudes and approaches, Skill and technique acquisition, Emotional distress, Self-Monitoring and insight). The HEIQ has previously been used in cancer patient and survivor populations (34-36). It allows to calculate a mean for each scale indicating higher or lower empowerment in the respective domain within a participant compared to the baseline assessment.

For peer review only - http://bmjopen.bmj.com/site/about/guidelines.xhtml

Secondary outcomes consist of a variety of patient-reported experiences and outcomes (PREMs and PROMs), such as satisfaction and quality of life (Figure 1).

3) What is the number and nature of pre-existing and new clinical events detected by PanCareFollowUp Care among participating survivors?

Clinical outcomes are outcomes of symptoms and diseases and have been defined based on published or almost published guidelines of the IGHG and the PanCareFollowUp Recommendations. A total of 116 clinical outcomes were defined, which reflects the wide range of late effects that survivors may encounter affecting both physical health and psychosocial wellbeing (Figure 1). Clinical outcomes include past and current medical history, are collected through survivor self-report in the Survivor Questionnaire (with verification at the clinic visit), and physician-report in the Treatment Summary, after the clinic visit and after potential diagnostic tests (Figure 2). The number and range of pre-existing and newly detected health problems (symptomatic and asymptomatic) per survivor will be described, including the results of clinical examinations (e.g. echocardiogram or blood tests).

4) What are the short-term (six months) and projected long-term costs per unit change of empowerment and other outcomes after implementing PanCareFollowUp Care from the perspective of survivors and HCPs?

The costs associated with implementing the care model will be determined by using health economic outcomes (Figure 1). These reflect the time, time off work and monetary investments made by the survivor, accompanying relatives or friends, the HCP and other staff in relation to the clinic visit while receiving or providing PanCareFollowUp Care, and are collected using questionnaires (Figure 2). We do not take costs outside the clinic visit into account, i.e., costs related to possible (follow-up) primary care physician visits, mental health services, or referrals to other specialists outside the clinical setting. Costs related to the clinic visit, as associated with PanCareFollowUp Care, are compared to potential benefits measured in terms of PREMs and PROMs.

An overall evaluation of implementing the PanCareFollowUp Care Intervention will be performed throughout the project according to the RE-AIM (Reach, Effectiveness, Adoption, Implementation and Maintenance) framework to assess the impact (www.re-aim.org) (37) (Table 1).

Table 1. RE-AIM framework applied to the PanCareFollowUp Care Intervention.

**Components**	**Related outcomes/actions in the Care Study**
Reach	• No. and proportion of participants vs. non-responders

For peer review only

	• Representativeness of participating survivors[a] (comparison of distribution: gender, current age, age at diagnosis and type of cancer)<br>• Reasons for (non-)participation
Effectiveness/efficacy	• Main outcome empowerment[a]<br>• Patient-reported outcome and experience measures, and clinical, feasibility and health economic outcomes[a]
Adoption[b]	• Multidisciplinarity of HCPs involved<br>• Recruitment rate<br>• Barriers and facilitators for recruitment
Implementation[b]	• Use of SCP and reasons for non-use<br>• Adaptations made to the PanCareFollowUp Care Intervention or implementation strategy<br>• Time and costs of PanCareFollowUp Care for survivors and HCPs<br>• Barriers and facilitators for implementation
Maintenance	• Replication Manual including updated implementation and recruitment strategy, publicly available for current and new centres<br>• Overview of requirements for study sites to make the PanCareFollowUp Care Intervention routine care

Abbreviations: HCPs = health care providers, SCP = Survivorship Care Plan. [a] Comparisons will be made according to subgroups of gender, current age, age at diagnosis and type of cancer. [b] This information will be collected at each study site separately.

Patient and public involvement

Survivor representatives from Childhood Cancer International-Europe are included in the project as members of the PanCareFollowUp Consortium (28). They are involved throughout the project and reach out to their respective national and international networks when needed. Survivors were involved in setting the research agenda by writing the grant application and the study protocol, developing and reviewing the PanCareFollowUp Care Intervention materials, evaluating the study questionnaires, monitoring the progress of the PanCareFollowUp Care Study and creating awareness on social media (29). They helped consider ways to mitigate the burden of completing the study questionnaires or remembering the childhood cancer history for participants. After the end of data

For peer review only - http://bmjopen.bmj.com/site/about/guidelines.xhtml

collection, survivor representatives will be involved in the interpretation of the study results and dissemination to participants, survivor networks and the general public.

Power calculation

We aim to include 200 participants at each of the four study sites (total n=800). The primary outcome measure is change in empowerment between T1 and T5 as measured by the HEIQ (34). We use six constructs (cancer version including five constructs plus one additional construct, namely self-monitoring and insight) with mean scores ranging from 2.9 (standard deviation (SD): 0.64) to 3.2 (SD: 0.48). Taking the construct with the largest SD (thus needing the highest number of participants to demonstrate a statistically significant change), limiting it to a single study site, with a 2-sided α of 0.05, a power of 80%, we will need 200 participants to identify an effect size of 0.2 given a mean score of 2.9 (SD: 0.64). That is enough power to demonstrate a small to medium effect. The actual power is larger since we ignored measuring empowerment repeatedly, having four centres (800 patients instead of 200) and using constructs with smaller SDs.

Data collection

Data will be collected from participating survivors as well as from their HCPs at five time points (T1-T5) during a follow-up period of six to eight months (Figure 2). We will use data collected in the context of care delivery, and combine it with additional data collected specifically for research purposes. For the latter, there are three data collection moments for survivors and four for HCPs. These time points are linked to the structure of the PanCareFollowUp Care Intervention, which consists of three steps: 1) Preparation of the clinic visit by survivor and HCP (corresponding with T1), 2) Clinic visit (corresponding with T2), and 3) Follow-up call (two to four weeks after T2, corresponding with T3). Thereafter, there is data collection at 1 week after the follow-up call (T4) and 6 months after the clinic visit (T5).

The main data collection instruments consist of the PanCareFollowUp Survivor Questionnaire (care), the Treatment Summary (care), medical history, physical examinations and diagnostic tests during and after the clinic visit (care), and additional online study questionnaires for survivors and HCPs (research). The Survivor Questionnaire and Treatment Summary are available through open access (29). The English versions of the study questionnaires for survivors have been pretested by three survivors, whereas the English questionnaires for HCPs have been pretested with at least two HCPs in each centre before the start of the data collection. The questionnaires for survivors have subsequently been translated to the local languages of the study sites, i.e. Czech, Dutch, Italian and Swedish.

For peer review only - http://bmjopen.bmj.com/site/about/guidelines.xhtml

Statistical analysis

For analysing outcomes measured multiple times, like the primary outcome, we will analyse multilevel models for repeated measures applying a fixed effect to control for study site. Next, we will perform subgroup analyses for relevant groups by including interaction terms. These subgroups will be identified based on the literature combined with knowledge from professionals. The final selection will be determined during the study. However, possible subgroups may be distinguished according to study site, sex, time since cancer diagnosis, treatment type, or distance to late effects clinic. The models will be adjusted for confounders, which will be identified during the study based on the literature and expert opinion. Clinical findings will be described at each time point, like the number of prevalent conditions as well as new diseases detected, diagnoses of sub-clinical diseases, relapse of the original tumour, late effects and diagnostic measurements. The results will be adjusted for multiple testing.

For the health economic evaluation, we will calculate the costs associated with the implementation of the PanCareFollowUp Care Intervention in order to achieve change in different outcomes. The analysis of costs and benefits will be based on within-subject changes until six months of follow-up, and on model-based evaluations for longer-term predictions. The estimated benefits of the intervention are measured in terms of empowerment (HEIQ) and quality of life (Short-Form 36 (SF-36), EQ-5D-5L, ICEpop CAPability measure for Adults (ICECAP-A)). Costs include resources incurred at the level of the hospital and the survivor. At the hospital level, we measure the time of physicians and other hospital staff for tasks related to the clinic visit and the follow-up call, costs for diagnostic and screening tests and other consumables for the clinic visit. At the survivor level, we measure the time investment and travel costs of survivors and relatives or friends, and loss of productive time at the workplace or in education. These costs are investigated separately on each level, hospital and survivor, as well as on an aggregated level.

The calculation of cost per unit change of outcomes needs to be interpreted in light of the relatively short follow-up period of six months within the study. This implies that the cost evaluation mainly focuses on short-run effects, while longer-run effects of PanCareFollowUp Care on outcomes such as survival cannot be measured within the study. Moreover, effects on other outcomes such as quality of life may be small. In order to provide information about the potential medium- to long-run effects, we will complement our analysis with a model-based economic evaluation approach using data from this study as well as information from the literature on longer-term effects of follow-up interventions and patient pathways, as well as related cost estimations. This will allow us to gain a

For peer review only - http://bmjopen.bmj.com/site/about/guidelines.xhtml

more comprehensive picture on the costs associated with the implementation of PanCareFollowUp Care.

Handling missing data

Automated reminders and phone calls by the clinics are used to ensure that all patients and HCPs complete all questionnaires to minimise the number of missing data. In case of missing data for certain PROMs and PREMs, we will replace missing values with the mean of the remaining items of the scale as recommended by the manuals. In case of other missing data, we will perform sensitivity analyses, i.e. perform the analyses with the complete cases and repeat the analyses with imputed values.

Data management

A cloud-based Electronic Data Capture platform has been developed by the Danish Cancer Society using Castor EDC (www.castoredc.com). This platform can be accessed by each of the four study sites for data entry. Castor EDC is compliant with all the important regulations regarding research: GDPR, ISO 27001 & ISO 9001 with servers located in the Netherlands including several measures to ensure security, adequacy and veracity of the collected data: regular back-ups (four times per day); personal accounts with individual user rights; audit, data and edit trail of all entered and changed data; and real-time edit checks to identify discrepancies in entered data.

Participating survivors complete their questionnaires directly in Castor EDC through a personalised link they receive by e-mail. Clinical data will be provided by HCPs or retrieved from survivors' medical records and entered into Castor EDC by local data managers according to a data entry instruction manual. All personal and sensitive data collected in the PanCareFollowUp project will be pseudonymised.

After the end of the data collection period, data will be exported from Castor to servers at the Danish Cancer Society. Experienced data managers will perform quality checks, data cleaning, and validation of data collected at the four sites and will set up data for the respective statistical analyses as subsets of the main database, governed by Data Transfer Agreements. The investigators will properly address all the ethical, legal, and safety aspects of the study and comply fully with EU Regulation 2016/679 on the protection of natural persons with regard to the processing of personal data and on the free movement of such data, and repealing Directive 95/46/EC (General Data Protection Regulation).

Ethics and dissemination

For peer review only - http://bmjopen.bmj.com/site/about/guidelines.xhtml

This study will be conducted in accordance with the guidelines of Good Clinical Practice by the International Council for Harmonisation of Technical Requirements for Pharmaceuticals for Human Use and the Declaration of Helsinki, written to protect those involved in clinical studies. The study protocol has been reviewed and approved by all relevant ethics committees: Brno, Ethics Committee of St. Anne's University Hospital (13 August 2019); Leuven, Ethics Committee Research University Hospitals Leuven (16 December 2020); Stockholm, Ethics Review Authority Stockholm (26 October 2020); Genoa, N. Liguria Regional Ethics Committee (13 July 2020).

Written informed consent will be obtained from all study participants before enrolment and data collection. An independent ethics advisor from Denmark is available to provide feedback and advice on ethics issues that may arise. An external study steering committee has been appointed to act as an advisory capacity with study oversight and external advice. The committee includes a survivor representative, a clinical oncologist, a late effects specialist, an ethicist and a statistician.

Incidental findings based on participants' completion of the questionnaires are unlikely given the nature of the questions, except for one question of the Brief Symptom Inventory-18 on suicidal thoughts. The central data centre and the four study sites will regularly check for any positive answers on this specific question, and inform the HCP as soon as possible, but within a maximum of two weeks. Worrisome answers at the pre-visit questionnaire will be discussed at the clinic visit. In the post-visit questionnaires, the survivor is informed that he or she can contact their general physician or late effects clinic in case of worrisome symptoms or complaints.

After the project, a Replication Manual will be developed for anyone interested in implementing the PanCareFollowUp Care for adult survivors of childhood cancer. It will include an updated Intervention Manual based on the Care Study results and additional focus groups with project stakeholders after the study closes. The Replication Manual will include all materials required for implementation in different languages and will become freely available through PanCare. PanCareFollowUp is aligned with EC Open Science Initiative, providing open access to all publications, and participates in the H2020 Open Research Data Pilot. The PanCareFollowUp Consortium will ensure that the collected data is findable, accessible, interoperable and reusable (FAIR). A dissemination plan including policy and press releases has been created warranting publications and lay language summaries on the different outcomes collected, to be distributed through the networks of PanCare and several (inter)national childhood cancer organisations. In addition, results will be published in peer-reviewed journals and presented on the project website.

Disclaimer

For peer review only - http://bmjopen.bmj.com/site/about/guidelines.xhtml

The material presented and views expressed here are the responsibility of the author(s) only. The EU Commission takes no responsibility for any use made of the information set out (Figure 3).

[Insert Figure 3 – EU Emblem]

Declarations

Protocol date and identifier

March 9th 2020, first version.

May 19th 2020, second version (adjustment in the paragraph about local data storage and transfer to central database).

January 21st 2021, third version (adjustment in the paragraph about data controllership and data processorship).

Protocol amendments

Protocol amendments, if any, will need to be approved by all investigators and are available upon request.

Consent for publication

Not applicable.

Availability of data and materials

Not applicable.

Competing interests

The authors declare that they have no competing interests.

Funding

This work was supported by the European Union's Horizon 2020 research and innovation programme [grant number 824982], and the Swedish Childhood Cancer Fund [grant number EU 2018-0002], and the Italian Ministry of Health [grant number not applicable]. The funding bodies and primary sponsor had no role in the design of the study; in the collection, management, analysis and interpretation of data; in writing of the report; or in the decision to submit the report for publication.

Primary sponsor

For peer review only - http://bmjopen.bmj.com/site/about/guidelines.xhtml

Princess Máxima Center for Pediatric Oncology

Heidelberglaan 25

3584 CS Utrecht, the Netherlands

Coordinator and contact for public and scientific queries

Prof Leontien C.M. Kremer (L.C.M.Kremer@prinsesmaximacentrum.nl)

Princess Máxima Center for Pediatric Oncology

Heidelberglaan 25

3584 CS Utrecht, the Netherlands

+31 88 9 72 72 72

For peer review only

Data monitoring committee

Not applicable, since this intervention is care as usual.

Auditing

Not applicable, since this intervention is care as usual.

Access to data

During the conduct of the Care Study, the study sponsor (Princess Máxima Center for Pediatric Oncology) will act as data controller, whereas the study sites are each joint controllers of the data collected at their own study site, and the Danish Cancer Society will act as data processor. Access to the data is regulated by a Data Processing Agreement between the Princess Máxima Center for Pediatric Oncology and the Danish Cancer Society, and by Study Site Agreements between the Princess Máxima Center for Pediatric Oncology and each of the four study sites. A Data Transfer Agreement between the Princess Máxima Center and specific project partners will govern the transfer of data for purposes of analysis after data collection has been completed.

Individual participant-level data (IPD) sharing

Public access to the full protocol, participant-level dataset and statistical code will be granted upon request, provided that their use is in agreement with the individual informed consent forms and contractual project agreements.

Author contributions

For peer review only - http://bmjopen.bmj.com/site/about/guidelines.xhtml

RK, JK, MR and LK contributed to the conception and design of the work and drafted and substantially revised the manuscript. RH, MM, TK, KK, AB, SB, LEF, SE, JFW, RH, AK, JL, GM, RM, KO, HP, SP, KR, RS, MR, AU, CF and LH contributed to the conception and design of the work and critically revised the manuscript. All authors read and approved of the final manuscript.

Acknowledgements

The authors gratefully acknowledge Carina Schneider and Patricia McColgan for organizing pilot tests of the study questionnaires with survivors, and Aoife Moggan, Rory McGrath and Daniel Owens for their participation in the pilot test. We acknowledge Anja Krøyer, Thomas Tjørnelund Nielsen and Agnethe Kirstine Møldrup Poulsen from the Childhood Cancer Research Group at the Danish Cancer Society Research Centre, Copenhagen, Denmark for setting up the PanCareFollowUp Care Study Castor database.

For peer review only

For peer review only - http://bmjopen.bmj.com/site/about/guidelines.xhtml

References

1. Gatta G, Botta L, Rossi S, Aareleid T, Bielska-Lasota M, Clavel J, et al. Childhood cancer survival in Europe 1999-2007: results of EUROCARE-5--a population-based study. Lancet Oncol. 2014;15(1):35-47.

2. Vassal G, Schrappe M, Pritchard-Jones K, Arnold F, Bassete L, Biondi A, et al. The SIOPE strategic plan: A European cancer plan for children and adolescents. J Cancer Policy. 2016;8:17-32.

3. Geenen MM, Cardous-Ubbink MC, Kremer LC, van den Bos C, van der Pal HJ, Heinen RC, et al. Medical assessment of adverse health outcomes in long-term survivors of childhood cancer. JAMA. 2007;297(24):2705-15.

4. Bhakta N, Liu Q, Ness KK, Baassiri M, Eissa H, Yeo F, et al. The cumulative burden of surviving childhood cancer: an initial report from the St Jude Lifetime Cohort Study (SJLIFE). Lancet. 2017;390(10112):2569-82.

5. Oeffinger KC, Mertens AC, Sklar CA, Kawashima T, Hudson MM, Meadows AT, et al. Chronic health conditions in adult survivors of childhood cancer. N Engl J Med. 2006;355(15):1572-82.

6. Daniel LC, Wang M, Mulrooney DA, Srivastava DK, Schwartz LA, Edelstein K, et al. Sleep, emotional distress, and physical health in survivors of childhood cancer: A report from the Childhood Cancer Survivor Study. Psychooncology. 2019;28(4):903-12.

7. Zeltzer LK, Lu Q, Leisenring W, Tsao JC, Recklitis C, Armstrong G, et al. Psychosocial outcomes and health-related quality of life in adult childhood cancer survivors: a report from the childhood cancer survivor study. Cancer Epidemiol Biomarkers Prev. 2008;17(2):435-46.

8. Font-Gonzalez A, Feijen E, Geskus RB, Dijkgraaf MGW, van der Pal HJH, Heinen RC, et al. Risk and associated risk factors of hospitalization for specific health problems over time in childhood cancer survivors: a medical record linkage study. Cancer Med. 2017;6(5):1123-34.

9. Steingart RM, Liu JE, Oeffinger KC. Cost-effectiveness of screening for asymptomatic left ventricular dysfunction in childhood cancer survivors. Ann Intern Med. 2014;160(10):731-2.

10. Wong FL, Bhatia S, Landier W, Francisco L, Leisenring W, Hudson MM, et al. Cost-effectiveness of the children's oncology group long-term follow-up screening guidelines for childhood cancer survivors at risk for treatment-related heart failure. Ann Intern Med. 2014;160(10):672-83.

11. Skinner R, Wallace WH, Levitt GA, Group UKCsCSGLE. Long-term follow-up of people who have survived cancer during childhood. Lancet Oncol. 2006;7(6):489-98.

12. Essig S, Skinner R, von der Weid NX, Kuehni CE, Michel G. Follow-up programs for childhood cancer survivors in Europe: a questionnaire survey. PLoS One. 2012;7(12):e53201.

13. Haupt R, Spinetta JJ, Ban I, Barr RD, Beck JD, Byrne J, et al. Long term survivors of childhood cancer: cure and care. The Erice statement. Eur J Cancer. 2007;43(12):1778-80.

For peer review only - http://bmjopen.bmj.com/site/about/guidelines.xhtml

14. Jankovic M, Haupt R, Spinetta JJ, Beck JD, Byrne J, Calaminus G, et al. Long-term survivors of childhood cancer: cure and care-the Erice Statement (2006) revised after 10 years (2016). J Cancer Surviv. 2018;12(5):647-50.

15. Kremer LC, Mulder RL, Oeffinger KC, Bhatia S, Landier W, Levitt G, et al. A worldwide collaboration to harmonize guidelines for the long-term follow-up of childhood and young adult cancer survivors: a report from the International Late Effects of Childhood Cancer Guideline Harmonization Group. Pediatr Blood Cancer. 2013;60(4):543-9.

16. Armenian SH, Hudson MM, Mulder RL, Chen MH, Constine LS, Dwyer M, et al. Recommendations for cardiomyopathy surveillance for survivors of childhood cancer: a report from the International Late Effects of Childhood Cancer Guideline Harmonization Group. Lancet Oncol. 2015;16(3):e123-36.

17. Clemens E, van den Heuvel-Eibrink MM, Mulder RL, Kremer LCM, Hudson MM, Skinner R, et al. Recommendations for ototoxicity surveillance for childhood, adolescent, and young adult cancer survivors: a report from the International Late Effects of Childhood Cancer Guideline Harmonization Group in collaboration with the PanCare Consortium. Lancet Oncol. 2019;20(1):e29-e41.

18. Clement SC, Kremer LCM, Verburg FA, Simmons JH, Goldfarb M, Peeters RP, et al. Balancing the benefits and harms of thyroid cancer surveillance in survivors of Childhood, adolescent and young adult cancer: Recommendations from the international Late Effects of Childhood Cancer Guideline Harmonization Group in collaboration with the PanCareSurFup Consortium. Cancer Treat Rev. 2018;63:28-39.

19. Skinner R, Mulder RL, Kremer LC, Hudson MM, Constine LS, Bardi E, et al. Recommendations for gonadotoxicity surveillance in male childhood, adolescent, and young adult cancer survivors: a report from the International Late Effects of Childhood Cancer Guideline Harmonization Group in collaboration with the PanCareSurFup Consortium. Lancet Oncol. 2017;18(2):e75-e90.

20. van Dorp W, Mulder RL, Kremer LC, Hudson MM, van den Heuvel-Eibrink MM, van den Berg MH, et al. Recommendations for Premature Ovarian Insufficiency Surveillance for Female Survivors of Childhood, Adolescent, and Young Adult Cancer: A Report From the International Late Effects of Childhood Cancer Guideline Harmonization Group in Collaboration With the PanCareSurFup Consortium. J Clin Oncol. 2016;34(28):3440-50.

21. van der Kooi ALF, Mulder RL, Hudson MM, Kremer LCM, Skinner R, Constine LS, et al. Counseling and surveillance of obstetrical risks for female childhood, adolescent, and young adult cancer survivors: recommendations from the International Late Effects of Childhood Cancer Guideline Harmonization Group. Am J Obstet Gynecol. 2020.

For peer review only - http://bmjopen.bmj.com/site/about/guidelines.xhtml

22. Christen S, Roser K, Mulder RL, Ilic A, Lie HC, Loonen JJ, et al. Recommendations for the surveillance of cancer-related fatigue in childhood, adolescent, and young adult cancer survivors: a report from the International Late Effects of Childhood Cancer Guideline Harmonization Group. J Cancer Surviv. 2020.

23. Mulder RL, Hudson MM, Bhatia S, Landier W, Levitt G, Constine LS, et al. Updated Breast Cancer Surveillance Recommendations for Female Survivors of Childhood, Adolescent, and Young Adult Cancer From the International Guideline Harmonization Group. J Clin Oncol. 2020:JCO2000562.

24. Michel G, Mulder RL, van der Pal HJH, Skinner R, Bardi E, Brown MC, et al. Evidence-based recommendations for the organization of long-term follow-up care for childhood and adolescent cancer survivors: a report from the PanCareSurFup Guidelines Working Group. J Cancer Surviv. 2019;13(5):759-72.

25. Mulder RL, van der Pal HJH, Levitt GA, Skinner R, Kremer LCM, Brown MC, et al. Transition guidelines: An important step in the future care for childhood cancer survivors. A comprehensive definition as groundwork. Eur J Cancer. 2016;54:64-8.

26. Loonen JJ, Blijlevens NM, Prins J, Dona DJ, Den Hartogh J, Senden T, et al. Cancer Survivorship Care: Person Centered Care in a Multidisciplinary Shared Care Model. Int J Integr Care. 2018;18(1):4.

27. Dutch Childhood Oncology Group. Guidelines for follow-up in survivors of childhood cancer 5 years after diagnosis. 2010. Available at: https://www.skion.nl/workspace/uploads/richtlijn_follow-up_na_kinderkanker_deel_1_1.pdf. Accessed 17 Dec 2021.

28. van Kalsbeek RJ, van der Pal HJH, Hjorth L, Winther JF, Michel G, Haupt R, et al. The European multistakeholder PanCareFollowUp project: novel, person-centred survivorship care to improve care quality, effectiveness, cost-effectiveness and accessibility for cancer survivors and caregivers. Eur J Cancer. 2021;153:74-85.

29. van Kalsbeek RJ, Mulder RL, Haupt R, Muraca M, Hjorth L, Follin C, et al. The PanCareFollowUp Care Intervention: A European harmonised approach to person-centred guideline-based survivorship care after childhood, adolescent and young adult cancer. Eur J Cancer. 2022;162:34-44.

30. Haupt R, Essiaf S, Dellacasa C, Ronckers CM, Caruso S, Sugden E, et al. The 'Survivorship Passport' for childhood cancer survivors. Eur J Cancer. 2018;102:69-81.

31. Osborne RH, Elsworth GR, Whitfield K. The Health Education Impact Questionnaire (heiQ): an outcomes and evaluation measure for patient education and self-management interventions for people with chronic conditions. Patient Educ Couns. 2007;66(2):192-201.

32. Luttrell C, Quiroz S, Scrutton C, Bird K. Working Paper 308: Understanding and operationalising empowerment. 2009. Available at: https://cdn.odi.org/media/documents/5500.pdf. Accessed 17 Dec 2021.

For peer review only - http://bmjopen.bmj.com/site/about/guidelines.xhtml

33. European Patients' Forum. Background Brief: Patient Empowerment. 2015. Available at: https://www.eu-patient.eu/globalassets/campaign-patient-empowerment/epf_briefing_patientempowerment_2015.pdf. Accessed 17 Dec 2021.

34. Maunsell E, Lauzier S, Brunet J, Pelletier S, Osborne RH, Campbell HS. Health-related empowerment in cancer: validity of scales from the Health Education Impact Questionnaire. Cancer. 2014;120(20):3228-36.

35. Brunet J, Lauzier S, Campbell HS, Fillion L, Osborne RH, Maunsell E. Measurement invariance of English and French Health Education Impact Questionnaire (heiQ) empowerment scales validated for cancer. Qual Life Res. 2015;24(10):2375-84.

36. Osborne RH, Batterham R, Livingston J. The evaluation of chronic disease self-management support across settings: the international experience of the health education impact questionnaire quality monitoring system. Nurs Clin North Am. 2011;46(3):255-70.

37. Glasgow RE, Harden SM, Gaglio B, Rabin B, Smith ML, Porter GC, et al. RE-AIM Planning and Evaluation Framework: Adapting to New Science and Practice With a 20-Year Review. Front Public Health. 2019;7:64.

For peer review only

For peer review only - http://bmjopen.bmj.com/site/about/guidelines.xhtml

Figure legends

Figure 1. Overview of all patient-reported outcome measures (PROMs) and experience measures (PREMs), clinical outcomes, feasibility outcomes and health economic outcomes used in the Care Study. Outcomes that are specific for males or females are indicated as such between brackets. For the clinical outcomes, it is indicated whether they are assessed through a diagnostic test according to the guidelines (d), Survivor Questionnaire (q), or both (d+q). Other clinical outcomes are assessed through medical history and/or physical examination. Abbreviations: BPI = Brief Pain Inventory, BSI-18 = Brief Symptom Inventory-18, CD-RISC 25 = Connor-Davidson Resilience Scale (25 items), ET = Emotion Thermometer, HCP = health care provider, HEIQ = health education impact questionnaire, HRQoL = health-related quality of life, ICECAP-A = ICEpop CAPability measure for Adults, LH/FSH = luteinising hormone/follicle-stimulating hormone, PROMIS = Patient-Reported Outcomes Measurement Information System, PCL-5 = PTSD Checklist for DSM-5, QoL = quality of life, Satisfaction Qx = Satisfaction questionnaire by Blaauwbroek et al, SCP = Survivorship Care Plan, SDM-Q-9 = 9-item shared decision-making questionnaire (patient perspective), SF-36 = Short Form-36 (36 items, version 1), SQx = Survivor Questionnaire (part of the PanCareFollowUp Care Intervention), TSH = thyroid-stimulating hormone, SDM-Q-Doc = 9-item Shared Decision-Making Questionnaire (HCP perspective).

References: [a] Brunet J., Lauzier S., Campbell H.S., Fillion L., Osborne R.H., Maunsell E., Measurement invariance of English and French Health Education Impact Questionnaire (heiQ) empowerment scales validated for cancer. Qual Life Res, 2015. 24(10): p. 2375-84.; Osborne R.H., Elsworth G.R., Whitfield K., The Health Education Impact Questionnaire (heiQ): an outcomes and evaluation measure for patient education and self-management interventions for people with chronic conditions. Patient Educ Couns, 2007. 66(2): p. 192-201.

[b] Blaauwbroek, R., Tuinier, W., Jong, B. M., Kamps, W. A., & Postma, A. (2008). Shared care by paediatric oncologists and family doctors for long-term follow-up of adult childhood cancer survivors: a pilot study. Lancet Oncology, 9(3), 232-238. DOI: 10.1016/S1470-2045(08)70034-2.

[c] Kriston L., Scholl I., Holzel L., Simon D., Loh A., Harter M., The 9-item Shared Decision Making Questionnaire (SDM-Q-9). Development and psychometric properties in a primary care sample. Patient Educ Couns, 2010. 80(1): p. 94-9.; Rodenburg-Vandenbussche S., Pieterse A.H., Kroonenberg P.M., Scholl I., van der Weijden T., Luyten G.P., et al., Dutch Translation and Psychometric Testing of the 9-Item Shared Decision Making Questionnaire (SDM-Q-9) and Shared Decision Making Questionnaire-Physician Version (SDM-Q-Doc) in Primary and Secondary Care. PLoS One, 2015. 10(7): p. e0132158.

For peer review only - http://bmjopen.bmj.com/site/about/guidelines.xhtml

[d] Connor K.M., Davidson J.R., Development of a new resilience scale: the Connor-Davidson Resilience Scale (CD-RISC). Depress Anxiety, 2003. 18(2): p. 76-82.

[e] EQ-5D-5L: Herdman, M., et al., Development and preliminary testing of the new five-level version of EQ-5D (EQ-5D-5L). Qual Life Res, 2011. 20(10): p. 1727-36.; SF-36: Ware J.E., Jr., Gandek B., Overview of the SF-36 Health Survey and the International Quality of Life Assessment (IQOLA) Project. J Clin Epidemiol, 1998. 51(11): p. 903-12.; ICECAP-A: Al-Janabi, H., T.N. Flynn, and J. Coast, Development of a self-report measure of capability wellbeing for adults: the ICECAP-A. Qual Life Res, 2012. 21(1): p. 167-76.

[f] Derogatis, L.R., BSI 18 - Brief Symptom Inventory 18 - Administration, Scoring, and Procedures Manual. 2000: NCS Pearson Inc.

[g] Blevins, C.A., et al., The Posttraumatic Stress Disorder Checklist for DSM-5 (PCL-5): Development and Initial Psychometric Evaluation. J Trauma Stress, 2015. 28(6): p. 489-98.

[h] Mitchell, A.J., et al., Can the Distress Thermometer be improved by additional mood domains? Part I. Initial validation of the Emotion Thermometers tool. Psychooncology, 2010. 19(2): p. 125-33., Mitchell, A.J., et al., Can the Distress Thermometer be improved by additional mood domains? Part II. What is the optimal combination of Emotion Thermometers? Psychooncology, 2010. 19(2): p. 134-40.

[i] Christen, S. et al., Recommendations for the Surveillance of Cancer-Related Fatigue in Childhood, Adolescent and Young Adult Cancer Survivors: A Report from the International Late Effects of Childhood Cancer Guideline Harmonization Group and the PanCare Guidelines Group. Journal of Cancer Survivorship. 2020;14(6):923-938.

[j] Bingham Iii, C.O., et al., PROMIS Fatigue short forms are reliable and valid in adults with rheumatoid arthritis. J Patient Rep Outcomes, 2019. 3(1): p. 14.

[k] Cleeland, C.S. and K.M. Ryan, Pain assessment: global use of the Brief Pain Inventory. Ann Acad Med Singapore, 1994. 23(2): p. 129-38.

Figure 2. Flowchart of data collection after inclusion of an eligible survivor. Abbreviations: HCP = health care provider, PREMS = patient-reported experience measures, PROMS = patient-reported outcome measures, T1 = time point 1, T2 = time point 2, T3 = time point 3, T4 = time point 4, T5 = time point 5. The boxes describe for each time point the timing of data collection, the person providing data (survivor, HCP or both), the data collection instruments (Survivor Questionnaire, Treatment Summary or T1-T5 study questionnaire) and the types of outcomes collected. Depicted in blue is data collected for care, and in purple for research purposes.

Figure 3: EU Emblem

For peer review only - http://bmjopen.bmj.com/site/about/guidelines.xhtml

For peer review only

**PROMs or PREMs: survivors**
Empowerment (HEIQ)[a] (primary outcome)
Patient satisfaction (Satisfaction Qx)[b]
Shared decision-making (SDM-Q-9)[c]
Resilience (CD-RISC 25)[d]
HRQoL (EQ-5D-5L, SF-36, ICECAP-A)[e]
Psychological distress (BSI-18)[f]
Post-traumatic stress symptoms (PCL-5)[g]
Distress (ET)[h]
Fatigue (SQx + PROMIS Fatigue – Short Form 8a +)[i,j]
Pain (BPI)[k]
Lifestyle (SQx)
Social functioning (SQx)
**Clinical outcomes**
Auditory
Hearing loss (d + q)
Tinnitus (q)
Cardiac
Arrhythmia (d + q)
Cardiomyopathy (d)
Pericardial disease (d)
Valvular heart disease (d)
Dental
Dental caries
Dental developmental problems
Xerostomia (q)
Dermatologic
Alopecia
Endocrine
ACTH deficiency (d)
Amenorrhea (females) (q)
Central precocious puberty (d)
Diabetes mellitus (d)
Failure in pubertal progression
Growth hormone deficiency (d)
Hyperthyroidism (d)
Hypothyroidism (peripheral) (d)
Impaired glucose metabolism (d)
LH/FSH deficiency (d)
Obesity
Overweight
Premature menopause (females) (d)
Premature ovarian insufficiency (females) (d)
Testosterone deficiency (males) (d)
TSH deficiency (d)
Gastro-intestinal
Bowel obstruction
Chronic enterocolitis
Gastro-intestinal strictures or fistula
Hepato-biliary
Cholelithiasis
Hepatobiliary dysfunction (d)
Hepatocellular liver injury (stage 1) (d)
Iron overload (d)
Liver cirrhosis
Liver fibrosis
Liver synthetic dysfunction (d)
Immunological
Spleen problems (overwhelming infections)
Musculoskeletal
Craniofacial growth problems
Osteonecrosis
Reduced bone mineral density (d)
Spine kyphosis
Spine scoliosis
Neurological
Cavernomas
Cerebrovascular accidents
Neurogenic bladder
Neurogenic bowel
Optic chiasm neuropathy
Pain (q)
Peripheral motor neuropathy (q)
Peripheral sensory neuropathy (q)
Psychosocial and neurocognitive
Adjustment difficulties
Anxiety (q)
Behavioural problems
Fatigue (q)
Low educational status (q)
Neurocognitive problems: academics
Neurocognitive problems: attention
Neurocognitive problems: executive function
Neurocognitive problems: intelligence
Neurocognitive problems: language
Neurocognitive problems: memory
Neurocognitive problems: motor integration
Neurocognitive problems: processing speed
Psychological distress (q)
Stress-related mental disorder
Suicidal ideation (q)
Unemployment (q)
Renal and urinary tract
Bladder fibrosis
Dysfunctional voiding (q)
Glomerular kidney dysfunction (d)
Haemorrhagic cystitis
Hydronephrosis
Tubular kidney dysfunction (d)
Vesicoureteral reflux
Reproductive
Impaired fertility (q)
Impaired spermatogenesis (males) (d + q)
Low birth weight of offspring (females) (q)
Miscarriage (females) (q)
Physical sexual dysfunction (males) (q)
Premature birth of offspring (females) (q)
Respiratory
Pulmonary dysfunction (d + q)
Subsequent neoplasm
Subsequent neoplasm (benign or malignant) (d + q)
Vascular
Aneurysms
Asymptomatic coronary artery disease
Carotid artery disease
Dyslipidaemia (d)
Hypertension
Visual
Cataract
Chronic painful eye
Glaucoma
Keratitis
Lacrimal duct atrophy
Maculopathy
Papillopathy
Retinopathy
Telangiectasias of the eye
Xerophthalmia
**Feasibility outcomes: survivor**
Received care according to SCP
Success of communication
Missing information
Italian study site only: Use of and satisfaction with SurPass
**Feasibility outcomes: HCP (per clinic)**
No. of eligible survivors invited
No. of participating survivors per time point
No. of non-responders
Reasons for non-response
No. of drop-outs per time point
Reasons for drop-outs per time point
Composition of multidisciplinary team
Use of the SCP
Reasons for non-use of SCP, if applicable
Shared decision making (HCP perspective; SDM-Q-Doc)[c]
Extent to which SCP of participating survivors has been implemented and reasons for deviating
Italian study site only: no. of SurPasses delivered, recommendation brochures given and SurPasses shared with physicians, SurPass user statistics
**Health economic outcomes: survivor**
Time investment of survivor (preparation for clinic visit, travel, total time in clinic, follow-up appointments)
Time investment of relatives (travel, total time in clinic, follow-up appointments)
Travel costs of survivor and relatives
Other extra costs for survivor and relatives
Loss of time for survivor and relatives at paid work or in education
**Health economic outcomes: HCP**
Time investment of HCP and other staff tasks related to clinic visit (preparation, clinic visit, tasks following clinic visit, follow-up call)
Costs for diagnostic and screening tests
Costs for other consumables for clinic visit

For peer review only

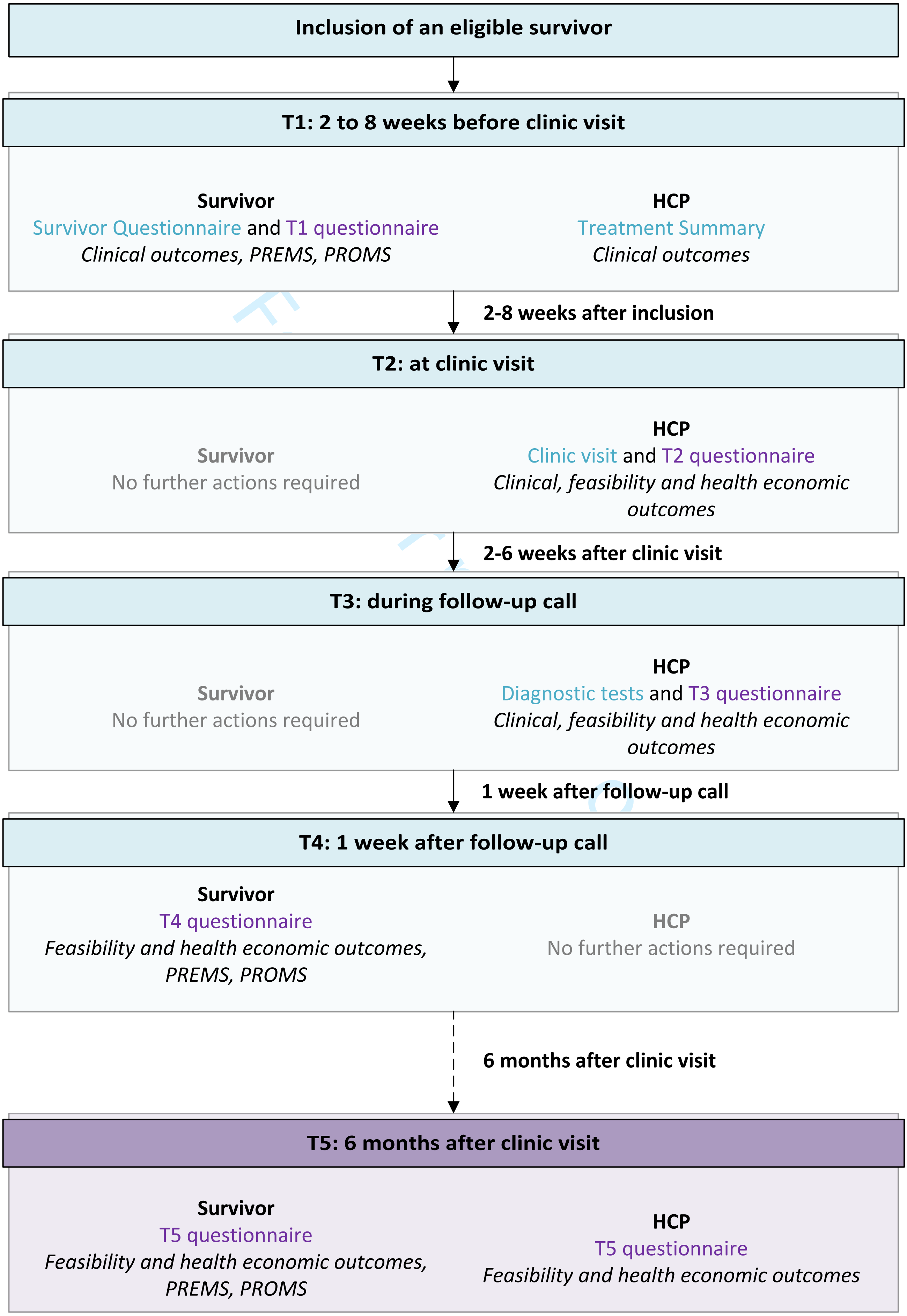

Inclusion of an eligible survivor
T1: 2 to 8 weeks before clinic visit
Survivor
Survivor Questionnaire and T1 questionnaire
Clinical outcomes, PREMS, PROMS
HCP
Treatment Summary
Clinical outcomes
2-8 weeks after inclusion
T2: at clinic visit
Survivor
No further actions required
HCP
Clinic visit and T2 questionnaire
Clinical, feasibility and health economic outcomes
2-6 weeks after clinic visit
T3: during follow-up call
Survivor
No further actions required
HCP
Diagnostic tests and T3 questionnaire
Clinical, feasibility and health economic outcomes
1 week after follow-up call
T4: 1 week after follow-up call
Survivor
T4 questionnaire
Feasibility and health economic outcomes, PREMS, PROMS
HCP
No further actions required
6 months after clinic visit
T5: 6 months after clinic visit
Survivor
T5 questionnaire
Feasibility and health economic outcomes, PREMS, PROMS
HCP
T5 questionnaire
Feasibility and health economic outcomes

For peer review only

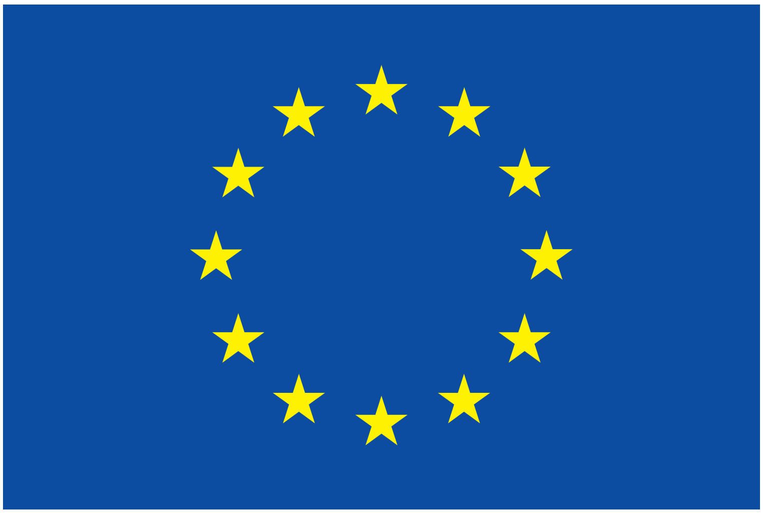

Appendix A: Recruitment strategy of each study site

Sweden starts with inviting a random sample, prioritising survivors who are lost to follow-up or have not visited the study site in the past five years, and might invite survivors who received care more recently depending on the recruitment rate among the initial population.

Italy starts with inviting survivors who already have a scheduled appointment at their clinic, but who had not already received the Survivorship Passport, and are resident in the Liguria region. They will invite 350 to 400 survivors to be able to include 200 survivors. They will subsequently recruit scheduled survivors resident in other regions, and if the number is still insufficient, they will actively invite other survivors to the clinic.

The Czech Republic starts with selecting a random sample of 250 survivors from the clinic's database whom they will gradually invite over the recruitment period. If more survivors need to be invited to reach the inclusion aim within the recruitment period, they will invite survivors who have a scheduled appointment at their clinic and who meet the study inclusion criteria

Belgium starts to invite, in alphabetical order the survivors of 18 year and older with a primary cancer diagnosis with a date of diagnosis in or before 1990, regardless of whether or not they already received some long-term follow-up. Simultaneously, 20 survivors who were scheduled for a clinic visit in March and April 2021 have also been invited to participate in this study. In the second wave, they will invite the survivors with a diagnosis in 1990-2000 in alphabetic order. And, if needed, the survivors diagnosed in 2001-2020, again in alphabetic order.

For peer review only

STANDARD PROTOCOL ITEMS: RECOMMENDATIONS FOR INTERVENTIONAL TRIALS

SPIRIT 2013 Checklist: Recommended items to address in a clinical trial protocol and related documents*

Section/item	Item No	Description	Addressed on page number
**Administrative information**			
Title	1	Descriptive title identifying the study design, population, interventions, and, if applicable, trial acronym	1
Trial registration	2a	Trial identifier and registry name. If not yet registered, name of intended registry	6
	2b	All items from the World Health Organization Trial Registration Data Set	1-25
Protocol version	3	Date and version identifier	8, 17, 18
Funding	4	Sources and types of financial, material, and other support	18
Roles and responsibilities	5a	Names, affiliations, and roles of protocol contributors	1,2
	5b	Name and contact information for the trial sponsor	18
	5c	Role of study sponsor and funders, if any, in study design; collection, management, analysis, and interpretation of data; writing of the report; and the decision to submit the report for publication, including whether they will have ultimate authority over any of these activities	18
	5d	Composition, roles, and responsibilities of the coordinating centre, steering committee, endpoint adjudication committee, data management team, and other individuals or groups overseeing the trial, if applicable (see Item 21a for data monitoring committee)	18, 19

For peer review only

**Introduction**			
Background and rationale	6a	Description of research question and justification for undertaking the trial, including summary of relevant studies (published and unpublished) examining benefits and harms for each intervention	7, 8
	6b	Explanation for choice of comparators	8, 11, 12
Objectives	7	Specific objectives or hypotheses	8
Trial design	8	Description of trial design including type of trial (eg, parallel group, crossover, factorial, single group), allocation ratio, and framework (eg, superiority, equivalence, noninferiority, exploratory)	9
**Methods: Participants, interventions, and outcomes**			
Study setting	9	Description of study settings (eg, community clinic, academic hospital) and list of countries where data will be collected. Reference to where list of study sites can be obtained	9
Eligibility criteria	10	Inclusion and exclusion criteria for participants. If applicable, eligibility criteria for study centres and individuals who will perform the interventions (eg, surgeons, psychotherapists)	9
Interventions	11a	Interventions for each group with sufficient detail to allow replication, including how and when they will be administered	8, 9, 10, ref 29
	11b	Criteria for discontinuing or modifying allocated interventions for a given trial participant (eg, drug dose change in response to harms, participant request, or improving/worsening disease)	10
	11c	Strategies to improve adherence to intervention protocols, and any procedures for monitoring adherence (eg, drug tablet return, laboratory tests)	N/A
	11d	Relevant concomitant care and interventions that are permitted or prohibited during the trial	N/A
Outcomes	12	Primary, secondary, and other outcomes, including the specific measurement variable (eg, systolic blood pressure), analysis metric (eg, change from baseline, final value, time to event), method of aggregation (eg, median, proportion), and time point for each outcome. Explanation of the clinical relevance of chosen efficacy and harm outcomes is strongly recommended	11, 12
Participant timeline	13	Time schedule of enrolment, interventions (including any run-ins and washouts), assessments, and visits for participants. A schematic diagram is highly recommended (see Figure)	10, Fig 2

For peer review only - http://bmjopen.bmj.com/site/about/guidelines.xhtml

Sample size	14	Estimated number of participants needed to achieve study objectives and how it was determined, including clinical and statistical assumptions supporting any sample size calculations	9, 10, 13, 14
Recruitment	15	Strategies for achieving adequate participant enrolment to reach target sample size	10, 15, App A

Methods: Assignment of interventions (for controlled trials)

Allocation:

Sequence generation	16a	Method of generating the allocation sequence (eg, computer-generated random numbers), and list of any factors for stratification. To reduce predictability of a random sequence, details of any planned restriction (eg, blocking) should be provided in a separate document that is unavailable to those who enrol participants or assign interventions	N/A
Allocation concealment mechanism	16b	Mechanism of implementing the allocation sequence (eg, central telephone; sequentially numbered, opaque, sealed envelopes), describing any steps to conceal the sequence until interventions are assigned	N/A
Implementation	16c	Who will generate the allocation sequence, who will enrol participants, and who will assign participants to interventions	N/A
Blinding (masking)	17a	Who will be blinded after assignment to interventions (eg, trial participants, care providers, outcome assessors, data analysts), and how	N/A
	17b	If blinded, circumstances under which unblinding is permissible, and procedure for revealing a participant's allocated intervention during the trial	N/A

Methods: Data collection, management, and analysis

Data collection methods	18a	Plans for assessment and collection of outcome, baseline, and other trial data, including any related processes to promote data quality (eg, duplicate measurements, training of assessors) and a description of study instruments (eg, questionnaires, laboratory tests) along with their reliability and validity, if known. Reference to where data collection forms can be found, if not in the protocol	14
	18b	Plans to promote participant retention and complete follow-up, including list of any outcome data to be collected for participants who discontinue or deviate from intervention protocols	10, 15, App A

Data management	19	Plans for data entry, coding, security, and storage, including any related processes to promote data quality (eg, double data entry; range checks for data values). Reference to where details of data management procedures can be found, if not in the protocol	15, 16
Statistical methods	20a	Statistical methods for analysing primary and secondary outcomes. Reference to where other details of the statistical analysis plan can be found, if not in the protocol	14, 15
	20b	Methods for any additional analyses (eg, subgroup and adjusted analyses)	14
	20c	Definition of analysis population relating to protocol non-adherence (eg, as randomised analysis), and any statistical methods to handle missing data (eg, multiple imputation)	14
**Methods: Monitoring**			
Data monitoring	21a	Composition of data monitoring committee (DMC); summary of its role and reporting structure; statement of whether it is independent from the sponsor and competing interests; and reference to where further details about its charter can be found, if not in the protocol. Alternatively, an explanation of why a DMC is not needed	19
	21b	Description of any interim analyses and stopping guidelines, including who will have access to these interim results and make the final decision to terminate the trial	N/A
Harms	22	Plans for collecting, assessing, reporting, and managing solicited and spontaneously reported adverse events and other unintended effects of trial interventions or trial conduct	17
Auditing	23	Frequency and procedures for auditing trial conduct, if any, and whether the process will be independent from investigators and the sponsor	19
**Ethics and dissemination**			
Research ethics approval	24	Plans for seeking research ethics committee/institutional review board (REC/IRB) approval	16
Protocol amendments	25	Plans for communicating important protocol modifications (eg, changes to eligibility criteria, outcomes, analyses) to relevant parties (eg, investigators, REC/IRBs, trial participants, trial registries, journals, regulators)	18

Consent or assent	26a	Who will obtain informed consent or assent from potential trial participants or authorised surrogates, and how (see Item 32)	10, 16
	26b	Additional consent provisions for collection and use of participant data and biological specimens in ancillary studies, if applicable	N/A
Confidentiality	27	How personal information about potential and enrolled participants will be collected, shared, and maintained in order to protect confidentiality before, during, and after the trial	15, 16, 19
Declaration of interests	28	Financial and other competing interests for principal investigators for the overall trial and each study site	18
Access to data	29	Statement of who will have access to the final trial dataset, and disclosure of contractual agreements that limit such access for investigators	19
Ancillary and post-trial care	30	Provisions, if any, for ancillary and post-trial care, and for compensation to those who suffer harm from trial participation	17
Dissemination policy	31a	Plans for investigators and sponsor to communicate trial results to participants, healthcare professionals, the public, and other relevant groups (eg, via publication, reporting in results databases, or other data sharing arrangements), including any publication restrictions	17
	31b	Authorship eligibility guidelines and any intended use of professional writers	19
	31c	Plans, if any, for granting public access to the full protocol, participant-level dataset, and statistical code	19
**Appendices**			
Informed consent materials	32	Model consent form and other related documentation given to participants and authorised surrogates	Submitted separately
Biological specimens	33	Plans for collection, laboratory evaluation, and storage of biological specimens for genetic or molecular analysis in the current trial and for future use in ancillary studies, if applicable	N/A

*It is strongly recommended that this checklist be read in conjunction with the SPIRIT 2013 Explanation & Elaboration for important clarification on the items. Amendments to the protocol should be tracked and dated. The SPIRIT checklist is copyrighted by the SPIRIT Group under the Creative Commons "Attribution-NonCommercial-NoDerivs 3.0 Unported" license.
